# Exploiting *Neosartorya* (*Aspergillus*) *fischeri* antifungal protein 2-defined fungal targets for drug repurposing against *Candida albicans*

**DOI:** 10.1128/spectrum.01310-26

**Published:** 2026-10-09

**Authors:** Kinga Dán, Rebeka Papp, Liliána Tóth, Lizett Miszlai, Gábor Bende, László Galgóczy

**Affiliations:** 1 Department of Biotechnology and Microbiology, Faculty of Science and Informatics, University of Szeged 37442, Szeged, Hungary; 2 Center for Reproductive Medicine, Department of Obstetrics and Gynecology, Albert Szent-Györgyi Health Centre, University of Szeged 37442, Szeged, Hungary; 3 Doctoral School of Biology, Faculty of Science and Informatics, University of Szeged 37442, Szeged, Hungary; 4 Department of Theoretical Health Sciences and Health Management, Faculty of Health Sciences and Social Studies, University of Szeged 37442, Szeged, Hungary; Seton Hall University, South Orange, New Jersey, USA; Tenth People's Hospital of Tongji University, Shanghai, China

**Keywords:** *Candida albicans*, antifungal protein, antifungal drug, drug repurposing, drug combination

## Abstract

Invasive fungal infections caused by *Candida albicans* remain major clinical challenges because of the limited therapeutic options, toxicity, and emerging resistance. Drug repurposing offers a promising strategy to accelerate antifungal discovery by leveraging existing compounds with known safety profiles. In this study, we applied a structure-based drug repurposing approach guided by the antifungal protein *Neosartorya* (currently *Aspergillus*) *fischeri* antifungal protein 2 (NFAP2), which targets fungus-specific intracellular proteins, including glutamate decarboxylase, mitochondrial ATP synthase subunit alpha, and enolase. NFAP2-associated binding pockets were identified and used to virtually screen approved clinically investigated compounds. Selected candidates were further evaluated by molecular docking, followed by *in vitro* antifungal susceptibility testing and interaction analysis with conventional antifungal agents. *In vivo* toxicity and therapeutic efficacy were assessed using a *Galleria mellonella* infection model. Despite predicted binding affinities, most repurposed compounds exhibited limited antifungal activity *in vitro*, highlighting a disconnect between *in silico* predictions and biological efficacy. Among the tested compounds, dihydroergocristine mesylate (DHEC) and meclizine dihydrochloride exhibited measurable antifungal effects. Notably, DHEC demonstrated pronounced synergistic interactions with amphotericin B, resulting in near-complete growth inhibition *in vitro. In vivo*, DHEC + amphotericin B induced no detectable toxicity and significantly improved host survival compared with either drug alone. These findings indicate that NFAP2-guided structure-based drug repurposing can identify compounds targeting fungus-specific vulnerabilities. However, antifungal activity depends on factors beyond binding affinity. Our results highlight integrating computational screening with experimental validation and support repurposed compounds as adjuvants in combination therapies to enhance antifungal efficacy and translational potential.

Invasive fungal infections caused by *Candida albicans* remain a major clinical challenge due to limited therapeutic options, toxicity, and emerging resistance. Drug repurposing offers a promising strategy to accelerate antifungal discovery by leveraging compounds with known safety profiles. A structure-based repurposing framework guided by the antifungal protein NFAP2, which targets fungus-specific intracellular proteins, is presented. Using these biologically informed targets, clinically relevant compounds with antifungal and adjuvant potential were identified. Predicted binding affinity alone did not reliably translate into antifungal activity, highlighting the need for integrated computational and experimental approaches. Notably, dihydroergocristine mesylate (DHEC) showed strong synergistic effects with amphotericin B, resulting in near-complete growth inhibition and improved host survival *in vivo*. These findings highlight the potential of repurposed compounds as adjuvants and support combination-based strategies targeting fungal-specific vulnerabilities. Furthermore, this work provides a framework for the future development of DHEC-inspired antifungal adjuvant strategies.

## INTRODUCTION

Invasive fungal infections represent a rapidly growing global health burden, causing particularly high mortality rates among immunocompromised patients, including those undergoing chemotherapy, organ transplantation, intensive care, or advanced immunomodulatory therapies. The World Health Organization (WHO) issued its first Fungal Priority Pathogens List in 2022, formally recognizing fungal pathogens as a neglected but critical threat to public health and calling for accelerated antifungal drug discovery and innovation (1). *Candida albicans* remains among the most clinically relevant fungal pathogens globally, being responsible for both mucosal and life-threatening systemic infections. Despite decades of clinical use, the current antifungal arsenal is extremely limited, comprising only a few drug classes [azoles, echinocandins, polyenes, and flucytosine (5-FC)], all of which exhibit significant drawbacks, including toxicity, drug–drug interactions, limited tissue penetration, and/or increasing resistance (2, 3). Notably, widespread fluconazole (FLC) use has driven the emergence of resistant *Candida* strains, leading to treatment failure even in standard-of-care settings (4).

The development of novel antifungal drugs through conventional *de novo* discovery pipelines is both expensive and time-consuming, and antifungal agent development is further hampered by biological complexity and limited market incentives (5, 6). Antifungal drug development is uniquely challenging because fungi are eukaryotic organisms closely related to human hosts, limiting potential selective targets and contributing to toxicity concerns. Alongside relatively small patient populations and short treatment courses, these factors severely limit the commercial attractiveness of antifungal drug development, resulting in a strikingly sparse clinical pipeline for agents targeting fungal pathogens prioritized by WHO (6–8). Drug repurposing offers a powerful alternative strategy to overcome these barriers by identifying new therapeutic applications for existing approved or clinically characterized drugs. Because repurposed compounds already have known pharmacokinetic and safety profiles, this approach can reduce development timelines by several years and lower costs by an order of magnitude vs *de novo* discovery (5, 9, 10). Repurposed drugs can be deployed either as standalone antifungals or as adjuvants that enhance the efficacy of existing therapies, including against resistant strains (11).

Ascomycetous antifungal proteins (AFPs) provide a biologically validated blueprint of fungus-specific vulnerability that can be exploited for drug repurposing strategies. These small, cysteine-rich peptides display potent and selective antifungal activity by targeting fungal-specific membrane components and intracellular pathways. Although their direct systemic use is constrained by unfavorable pharmacokinetic properties, including poor *in vivo* stability and limited bioavailability (12, 13), their well-defined interaction sites on essential fungal targets provide structurally tractable templates for identifying small-molecule drugs that can recapitulate their fungus-specific mechanisms of action. Among AFPs, *Neosartorya* (currently *Aspergillus*) *fischeri* antifungal protein 2 (NFAP2) has emerged as an especially promising molecule because of its strong fungal-specific activity against *Candida* species including drug-resistant clinical isolates (14–16), low intrinsic resistance potential (17), and synergistic interactions with conventional antifungal drugs (16). NFAP2 exhibits a dual mode of action, combining selective disruption of fungal cell membranes with binding to intracellular fungal-specific targets in *C. albicans*, thereby triggering cell death through fundamentally distinct mechanisms from those of current antifungal drug classes (18).

Crucially, our recent work demonstrated that NFAP2 recognizes and binds defined intracellular proteins in *C. albicans* (including glutamate decarboxylase [Gad1p], which is involved in amino acid metabolism and stress adaptation, mitochondrial ATP synthase subunit alpha [Atp1p], a central component of cellular energy production, and enolase [Eno1p], a key glycolytic enzyme] in a fungus-specific manner by engaging pre-existing binding pockets, with structurally distinct interaction sites identified for Gad1p and Eno1p compared with their human homologs (18). This property both underlies its favorable safety profile and provides a unique opportunity for structure-guided drug repurposing. Using NFAP2-associated fungal targets as molecular templates, it is possible to identify small-molecule drugs that mimic the binding behavior of NFAP2, recapitulating its antifungal effects in a pharmacologically tractable format. This approach leverages the biological selectivity of AFPs while circumventing their inherent limitations as peptide therapeutics, enabling the rational identification of systemically applicable antifungal agents from existing drug libraries.

In this study, we applied an NFAP2-guided drug repurposing strategy to identify clinically available compounds targeting NFAP2-associated intracellular proteins in *C. albicans*, such as Gad1p, Atp1p, and Eno1p (18). We evaluated the antifungal and adjuvant potential of prioritized candidates *in vitro* and assessed their *in vivo* efficacy and toxicity in a proof-of-concept infection model. This integrative approach established a translational framework for exploiting AFP-defined fungal targets to discover systemically applicable antifungal therapies.

## MATERIALS AND METHODS

### Structure-based *in silico* drug repurposing

Amino acid residues involved in the binding between NFAP2 and its intracellular targets in *C. albicans* (i.e., Atp1p, Eno1p, Gad1p) were identified using the binding analysis function of UCSF Chimera v1.16 (Tools → Binding Analysis → Find Clashes/Contacts; 18, 19). Based on the identified residues, the NFAP2-binding pockets located of the interacting partner proteins were mapped. The three-dimensional structure of NFAP2 was obtained from the RCS Protein Data Bank (ID: 8RP9; 19). Structural models of Atp1p, Eno1p, and Gad1p were obtained as AlphaFold2-predicted structures downloaded from the UniProt database and used as receptors for subsequent analyzes (20). *In silico* drug screening was subsequently performed on the selected binding pockets using the receptor-based screening module of the DrugRep server (21) against an approved drug database to identify compounds predicted to interact with NFAP2-associated binding pockets and potentially recapitulate their antifungal activity.

For structure-based validation of the selected compounds, molecular docking was performed using AutoDock Vina, as implemented in UCSF Chimera (22). Receptor structures (*C. albicans* Atp1p, Eno1p, and Gad1p) were prepared by removing solvent molecules, adding hydrogen atoms, and assigning Gasteiger charges. Ligand structures (repurposed compounds) were prepared in a compatible format prior to docking. Docking grids were centered on NFAP2-defined binding pockets identified by residue-level interaction analysis, and grid dimensions were adjusted to encompass the entire interaction region. Docking simulations were performed using default Vina parameters. Docking poses were evaluated according to the predicted binding affinity and spatial overlap with NFAP2 interaction sites. For visualization and figure preparation, the docked complexes and NFAP2 were displayed simultaneously using UCSF Chimera (22), allowing direct comparison of ligand-binding positions relative to NFAP2-defined interfaces.

### *In vitro* antifungal susceptibility testing

The antifungal activity of the repurposed compounds was evaluated using *in vitro* microdilution assays. From the stock solutions of the drug molecules, an 11-step dilution series was prepared. In the first step, drug molecules dissolved in DMSO were subjected to serial twofold dilutions in DMSO. In the second step, the DMSO-diluted compounds were further diluted 50-fold in low-cationic medium (LCM: 0.5% glucose, 0.25% yeast extract, 0.0125% peptone [wt/vol]) or RPMI-1640 (Sigma-Aldrich, St. Louis, MO, USA), resulting in a final concentration of 1,024 μg/mL. All dilution steps were performed following the Clinical and Laboratory Standards Institute (CLSI) M27-A4 guideline (23). Mid-log phase *C. albicans* SC5314 cells from an overnight culture (10 mL of LCM, 30°C, 160 rpm) were diluted to 2 × 10^5^ cells/mL in LCM and 2 × 10^3^ cells/mL in RPMI-1640. The *in vitro* antifungal activity of the antifungal drug candidates was assessed in 96-well flat-bottom microtiter plates (TC Plate 96 Well, Suspension, F; Sarstedt, Nümbrecht, Germany). Each well contained 100 μL of the *C. albicans* suspension and 100 μL of the drug molecule at the appropriate concentration. Growth control wells contained 100 μL of the fungal suspension and 100 μL of LCM or RPMI-1640. Microtiter plates were statically incubated at 30°C for 48 h. Optical density at 620 nm (OD_620_) was measured at 0 and 48 h using a microplate reader (SPECTOstar Nano, BMG Labtech, Offenburg, Germany). Fresh LCM or RPMI-1640 was used for background calibration. Prior to measurement, *C. albicans* cells were resuspended. The minimum inhibitory concentration (MIC) of drug molecules was defined as the lowest concentration that reduced fungal growth to ≤5% of the untreated control level (OD_620_ set to 100%). This endpoint differs from conventional CLSI MIC definitions and was intentionally selected to provide a conservative estimate of near-complete growth inhibition. Residual growth below this threshold was considered to fall within the range of experimental background variation associated with OD-based measurements and biological replicate-to-replicate variability. The stringent MIC endpoint was selected to identify concentrations producing near-complete and sustained growth inhibition under prolonged exposure conditions and to provide a consistent basis for the subsequent interaction analyses. The half-maximal inhibitory concentration (IC_50_) was defined as the concentration that reduced fungal growth by at least 50% relative to the untreated control after 48 h of incubation. Microdilution assays were performed at least twice, with each experiment including two technical replicates. Although the assay was based on the CLSI M27-A4 methodology (23), several parameters were intentionally modified, including incubation at 30°C, a 48-h incubation period, and the stringent MIC endpoint definition described above. These conditions were selected to maintain consistency with the NFAP2-related experiments and the subsequent interaction studies performed throughout this work. Consequently, the inhibitory concentrations reported in this study should be interpreted within the context of this experimental system rather than as CLSI-reference MIC values. The modified CLSI-based microdilution assay described above was also applied to determine the MICs of conventional antifungal drugs (all from MedChemExpress, Monmouth Junction, NJ, USA) in RPMI-1640, including amphotericin B (AMB), FLC, 5-FC, and micafungin (MCF), against *C. albicans* SC5314. The tested concentration range was 0.03125–8 µg/mL for AMB, FLC, MCF, and 5-FC.

### Additional validation of micafungin susceptibility

To verify that the elevated MCF concentrations observed under the modified assay conditions used in this study were not attributable to strain contamination or altered strain susceptibility, an additional validation experiment was performed. Susceptibility testing was independently repeated under standard CLSI M27-A4 conditions using two independent *C. albicans* SC5314 stocks, one maintained in our laboratory and one obtained from the Host–Pathogen Interaction Centre (University of Szeged) (23). For each strain, two independent biological experiments were performed, each including three technical replicates. MIC values were determined after 24 h of incubation at 35°C using the conventional ≥50% growth inhibition endpoint (23). The same cultures were subsequently incubated for an additional 24 h and re-evaluated after a total of 48 h using the stringent near-complete growth inhibition criterion employed throughout this study. Representative photographs of the microtiter plates, microscopic observations, and the corresponding susceptibility data are provided in the Table S1.

### *In vitro* interactions between repurposed compounds and antifungal agents

The checkerboard titration method was employed to investigate the interactions between repurposed compounds and conventional antifungal drugs against *C. albicans* SC5314. RPMI-1640 was used for compound dilution and cell suspension preparation. Dilutions were performed as previously described, except that a nine-step twofold serial dilution series was employed. Final concentration ranges in 96-well flat-bottom microtiter plates were established as follows: dihydroergocristine mesylate (DHEC), 128–0.5 μg/mL; meclizine dihydrochloride (MECD), 256–1 μg/mL; AMB, 0.5–0.0075 μg/mL; FLC, 0.5–0.0075 μg/mL; 5-FC, 0.125–0.00375 μg/mL; and MCF, 1–0.015 μg/mL. Experiments were conducted in a total volume of 200 μL per well, consisting of 50 μL of the repurposed compound solution, 50 μL of the antifungal agent solution, and 100 μL of a *C. albicans* SC5314 cell suspension adjusted to 2 × 10^3^ cells/mL. This approach permitted the evaluation of different concentration combinations below the individually determined MICs, facilitating the investigation of potential combinatorial effects on *C. albicans* growth. Plates were incubated statically at 30°C for 48 h. After incubation and resuspension, the percent growth in treated cultures was calculated from OD_620_ as follows: percent growth = (absorbance of treated culture × 100)/absorbance of untreated culture. The untreated control was defined as 100% growth, and 200 µL of fresh medium was used as the spectrophotometric blank. Drug–drug interactions were evaluated using the interaction ratio (IR) according to the Abbott formula (24), where IR = Io/Ie, and Ie = *X* + *Y* − (*XY*/100). In this context, *X* and *Y* correspond to the inhibitory effects of the individual agents, and Io represents the experimentally observed inhibition in percentages. This approach was selected instead of the fractional inhibitory concentration index (FICI), as the determination of FICI requires the MIC for each compound, which could not be reliably established for the repurposed compounds because of their limited antifungal activity under the tested conditions. Interactions were interpreted as additive (1.5 > IR > 0.5), synergistic (IR > 1.5), or antagonistic (IR < 0.5) according to previously published Abbott formula-based interaction analyses (24). Because IR values quantify deviations from the expected additive effect rather than the absolute magnitude of growth inhibition, interaction ratios were interpreted together with the corresponding inhibitory activity values. No interaction was assumed when the combined effect was similar to that of the stronger individual compound. Each experiment was independently repeated twice with two technical replicates.

### *Galleria mellonella* toxicity assay

The potential *in vivo* toxicity of DHEC and AMB, both alone and in combination, was investigated in *G. mellonella* larvae for 512 μg/mL DHEC, 1 μg/mL AMB, and 2 μg/mL DHEC + 0.5 µg/mL AMB. Solutions were prepared in insect physiological saline (IPS; 50 mM NaCl, 5 mM KCl, 10 mM EDTA, and 30 mM sodium citrate dissolved in 0.1 M Tris-HCl, pH 6.9), and 20 µL were injected intrahemocoelically into the last right proleg in 20 larvae per group using 29-gage insulin needles (BD Micro-Fine, Becton, Dickenson and Company, Franklin Lakes, NJ, USA). Prior to injection, larvae were maintained overnight at 37°C. Post-injection, larvae were incubated at 37°C, and survival was monitored every 24 h for 6 days. IPS-treated larvae served as negative controls for toxicity, 20% (vol/vol) Triton X-100-treated larvae served as positive toxicity controls, and larvae without any treatment served as untreated controls. The toxicity experiment was independently performed twice.

### *In vivo* therapeutic efficacy of repurposed compounds and antifungal drug combinations

The *in vivo* therapeutic potential of DHEC and AMB, both as monotherapies and in combination, was evaluated in *G. mellonella* larvae following the protocol established for the toxicity assay. The tested concentrations were selected based on the *in vitro* susceptibility and checkerboard interaction analyses. Monotherapy treatments were performed using the MIC determined for AMB in RPMI-1640 (1 µg/mL). Because DHEC did not reach the predefined MIC endpoint under the applied assay conditions (MIC > 512 µg/mL), the highest concentration tested during susceptibility testing (512 µg/mL) was used for DHEC monotherapy. The combination treatment was selected from the most effective DHEC (2 µg/mL) + AMB (0.5 µg/mL) interaction identified *in vitro*, which exhibited strong synergy and near-complete growth inhibition under the experimental conditions employed in this study (Table 2). In addition to the previously described procedures, larvae were infected via an intrahemocoelic injection of 20 µL of a fungal suspension containing 2.5 × 10^7^ *C. albicans* SC5314 cells/mL into the last left proleg. In the nontoxic and positive toxicity control groups, 20 µL of IPS was injected instead of the fungal suspension. The experiment was independently performed twice with 20 larvae per experiment. Statistical analysis of *G. mellonella* survival was conducted using the log-rank (Mantel–Cox) and Gehan–Breslow–Wilcoxon tests in GraphPad Prism 8.00 (GraphPad Software, Boston, MA, USA). *P* ≤ 0.05 indicated statistically significant differences in both analyses.

## RESULTS

### Structure-guided repurposing identified compounds targeting NFAP2-associated intracellular binding sites

Based on our previous results, the NFAP2-interacting intracellular proteins Gad1p, Atp1p, and Eno1p of *C. albicans*, along with their corresponding NFAP2-associated binding pockets, were identified (18). These binding pockets were used as target sites for the structure-based screening of approved or clinically investigated compounds from established drug databases, aiming to identify molecules that retain antifungal activity and a fungus-specific mechanism of action while exhibiting favorable pharmacokinetic and pharmacodynamic properties.

Structure-based virtual screening was performed using the DrugRep server (21). For each of the potential NFAP2 target proteins, NFAP2-associated binding pockets defined by prior *in silico* docking analyses were selected as the target regions (18). Screening yielded multiple candidate compounds (Table 1; Fig. S1) exhibiting varying predicted binding affinities toward one or more of the identified binding pockets (Table 1).

**TABLE 1:** Predicted binding and binding energies of repurposed compounds for *Neosartorya* (currently *Aspergillus*) *fischeri* antifungal protein 2-binding pockets in *Candida albicans*, their indication, and experimentally determined antifungal activities (minimum inhibitory concentration, MIC) in low-cationic medium (LCM) and RPMI-1640

Drug molecule (abbreviation)	Binding energy^*a*^			Primary indication	MIC (µg/mL)^*b*^	
	Gad1p	Atp1p	Eno1p		LCM	RPMI-1640
Bictegravir	–6.1	–^*c*^	–	HIV infection	>512	n.t.
Digitoxin	–7.2	–8.8	–	Heart failure/arrhythmia	>512	n.t.
Dihydroergocristine mesylate	–7.3	–8.4	–	Cognitive impairment/vascular disorders	256	>512
Droperidol	–	–	–6.2	Antiemetic/sedation	>512	n.t.
Glecaprevir	–6.5	–8.4	–	Hepatitis C infection	>512	n.t.
Lurasidone	–6.5	–7.3	–6.3	Schizophrenia/bipolar depression	>512	n.t.
Meclizine dihydrochloride	–	–	–4.6	Motion sickness/vertigo	256	256 (IC_50_)
Midostaurin	–6.4	–7.5	–6.5	Acute myeloid leukemia	>512	n.t.
Simeprevir	–7.5	–8	–	Hepatitis C infection	512 (IC_50_)	n.t.
Talazoparib	–	–7.1	–7.5	Breast cancer (BRCA-mutated)	>512	n.t.
Tucatinib	–6.5	–7.6	–6.7	HER2-positive breast cancer	>512	n.t.
Venetoclax	–7.7	–9.3	–7.9	Chronic lymphocytic leukemia	>512	n.t.

^*a*^ Binding energies were calculated by the DrugRep server as part of the structure-based virtual screening workflow (21).

^*b*^ MIC and IC_50_ values were determined using the modified CLSI-based assay conditions described in the Materials and Methods section. Reported MIC values should not be interpreted as CLSI-reference MICs. For compounds that did not reach the predefined MIC endpoint, IC_50_ values are reported where applicable. n.t., not tested.

^*c*^ –, no predicted binding to the indicated NFAP2-associated binding pocket was identified in the structure-based virtual screening.

Based on DrugRep-calculated binding energies, ≤−10 kcal/mol indicated very strong binding, −9 to −8 kcal/mol indicated strong binding, −7 to −6 kcal/mol indicated moderate binding, and >−5 kcal/mol corresponded to weak interactions. Among the screened compounds, venetoclax, digitoxin, DHEC, glecaprevir, and simeprevir (SIM) exhibited the strongest predicted interactions with NFAP2-associated binding pockets with binding energies <−7 kcal/mol for Gad1p, <−8 kcal/mol for Atp1p, and <−7.5 kcal/mol for Eno1p (Table 1; Fig. S2). Conversely, MECD displayed weak binding affinity and target selectivity, interacting only with Eno1p (Table 1). Similarly, droperidol also exhibited selective interaction with Eno1p, albeit with slightly stronger predicted binding. Overall, Eno1p-associated interactions were generally weaker compared to those observed for Gad1p and Atp1p (Table 1). These docking results provide a structural rationale for potential interactions with the identified NFAP2 target proteins. However, predicted binding affinity alone does not necessarily translate into biological activity. Therefore, experimental validation is required to determine whether these interactions result in measurable antifungal effects and assess whether the compounds can recapitulate NFAP2-like, fungus-specific mechanisms of action under physiological conditions. To address this, subsequent *in vitro* susceptibility assays were performed to evaluate antifungal efficacy and examine the relationship between predicted binding affinity and biological activity.

### *In vitro* validation revealed limited antifungal activity of computationally prioritized repurposed compounds

To validate the *in silico* predictions, the antifungal activities of the repurposed compounds identified through structure-based screening were assessed *in vitro* against *C. albicans* SC5314 in LCM. Antifungal susceptibility testing enabled the determination of both MIC and IC_50_. Although several compounds exhibited strong predicted binding affinities in the docking analysis, most did not display significant antifungal activity, even at the highest tested concentration (512 μg/mL). These results indicate that high predicted binding affinity does not necessarily translate into measurable biological efficacy under the applied experimental conditions. Consistent with this observation, no apparent relationship was detected between predicted binding energies (Table 1) and experimentally determined antifungal activity. For example, venetoclax, digitoxin, and glecaprevir exhibited some of the strongest predicted interactions with NFAP2-associated targets, yet failed to demonstrate measurable antifungal activity, whereas MECD displayed only weak predicted binding but retained detectable biological activity.

Among the tested compounds, SIM inhibited growth by approximately 50% at the highest tested concentration (512 μg/mL), consistent with partial antifungal activity. Contrarily, DHEC and MECD induced complete growth inhibition, each at 256 μg/mL, corresponding to the MIC endpoint defined in this study. These findings indicate that only a subset of the computationally prioritized compounds exerted biologically relevant antifungal effects *in vitro*.

The antifungal efficacy of the highly effective compounds (DHEC and MECD) was further evaluated in RPMI-1640 using a modified CLSI M27-A4-based broth microdilution assay (23). This additional testing was performed because RPMI-1640 is a clinically standardized medium that better reflects physiological conditions and potentially improves the prediction of *in vivo* efficacy. Compared with the low ionic strength of LCM, RPMI 1640 has higher ionic strength and a composition more similar to human serum. Under these conditions, DHEC did not exhibit detectable antifungal activity (MIC > 512 µg/mL), whereas MECD inhibited growth by approximately 50% at 256 μg/mL.

These findings indicated that the investigated repurposed compounds are unlikely to be suitable as standalone antifungal agents, as the high concentrations required to achieve the desired inhibitory effect would likely be associated with considerable adverse effects. However, their potential application as adjuvant agents in combination therapy remained promising (11).

### Repurposed compounds exhibited synergistic and antagonistic interactions with major antifungal drug classes

To investigate the potential adjuvant properties of compounds exhibiting moderate antifungal activity in RPMI-1640 (i.e., DHEC, MECD), their interactions with clinically relevant antifungal agents were systematically evaluated. Drug–drug interactions were analyzed using the checkerboard microdilution method to determine whether combinatorial effects resulted in synergistic, additive, or antagonistic outcomes. DHEC and MECD were tested in combination with AMB (polyene), FLC (azole), 5-FC (pyrimidine analog), and MCF (echinocandin), representing the four major antifungal drug classes used to treat *C. albicans* infections (3).

Prior to interaction analysis, the inhibitory activities of these agents were determined against *C. albicans* in RPMI-1640 using the modified CLSI M27-A4-based broth microdilution assay described above (23). Under the applied experimental conditions, the MIC was 1 μg/mL for AMB, 0.125 μg/mL for 5-FC, and 1 μg/mL for MCF. The IC_50_ for FLC was 0.5 μg/mL, whereas the predefined MIC endpoint (≤5% residual growth relative to the untreated control) was not reached. This observation is consistent with the well-described phenomenon of azole trailing growth, in which residual growth persists above the apparent MIC despite increasing fluconazole concentrations, particularly during prolonged incubation periods (25, 26).

Interactions between repurposed compounds and antifungal drugs were evaluated using the IR calculated according to the Abbott formula. Importantly, if the inhibition observed in combination did not exceed that produced by the more active single component, the result was not considered a true interaction irrespective of the calculated IR.

In the case of DHEC, synergistic interactions were observed with AMB (Table 1), FLC, and MCF, whereas an antagonistic interaction was detected with 5-FC (Table S2). Near-complete growth inhibition was achieved with combinations of DHEC and AMB (Table 1) and DHEC and MCF within the following concentration ranges: 2–128 μg/mL DHEC + 0.5 µg/mL AMB, 32–64 μg/mL DHEC + 0.25 µg/mL AMB (Table 2), and 32–128 μg/mL DHEC + 0.25 µg/mL MCF (Table S2).

**TABLE 2:** Inhibitory activity and interaction analysis of dihydroergocristine mesylate (DHEC) and amphotericin B (AMB) combinations against *Candida albicans* SC5314^*a*^

DHEC→/AMB↓ (μg/mL)	128	64	32	16	8	4	2	1	0.5	0
2	100% ± 0.62%	99% ± 0.91%	99% ± 1.34%	99% ± 0.94%	100% ± 0.48%	100 ± 0.85%	100 ± 0.21%	100 ± 0.59%	100±0.59%	100±0.40%
	**NI: 1 ± 0**	**NI: 0.99 ± 0.01**	**NI: 0.99 ± 0.02**	**NI: 0.99 ± 0.01**	**NI: 1 ± 0.01**	**NI: 1 ± 0.01**	**NI: 1 ± 0**	**NI: 1 ± 0**	**NI: 1 ± 0.01**	
1	99% ± 0.86%	99% ± 0.92%	99% ± 1.13%	99% ± 1.31%	99% ± 0.66%	99 ± 0.84%	100 ± 0.23%	100 ± 0.28%	99±0.30%	100±0.46%
	**NI: 0.99 ± 0.01**	**NI: 0.99 ± 0.01**	**NI: 0.99 ± 0.01**	**NI: 0.99 ± 0.01**	**NI: 0.99 ± 0.01**	**NI: 0.99 ± 0.01**	**NI: 1 ± 0**	**NI: 1 ± 0**	**NI: 0.99 ± 0**	
0.5	100% ± 0.71%	100% ± 0.61%	98% ± 1.40%	98% ± 0.92%	99% ± 1.04%	99 ± 0.30%	99 ± 0.98%	96 ± 5.77%	65±35.46%	34±24.53%
	**S: 1.70 ± 1.20**	**S: 1.71 ± 1.21**	**S: 1.69 ± 1.21**	**S: 1.69 ± 1.20**	**S: 1.70 ± 1.21**	**S: 1.69 ± 1.19**	**S: 1.69 ± 1.20**	**S: 1.62 ± 1.13**	**NI: 0.82 ± 0.66**	
0.25	98% ± 3.89%	99% ± 0.84%	99% ± 1.01%	99% ± 1.06%	99% ± 0.88%	98 ± 1.01%	45 ± 12.23%	32 ± 17.91%	33±19.79%	35±18.19%
	**S: 4.61 ± 4.76**	**S: 4.64 ± 4.71**	**S: 4.60 ± 4.64**	**S: 4.61 ± 4.67**	**S: 4.60 ± 4.64**	**S: 4.56 ± 4.60**	**S: 2.23 ± 2.49**	**NI: 1.65 ± 2.06**	**NI: 1.97 ± 2.77**	
0.125	90% ± 11.68%	96% ± 1.32%	98 ± 1.11%	85 ± 15.42%	56 ± 34.02%	35 ± 19.74%	14 ± 19.47%	23 ± 22.41%	9±10.88%	8±9.64%
	**S: 2.80 ± 3.54**	**S: 3.24 ± 3.93**	**S: 3.27 ± 4**	**S: 2.44 ± 3.07**	**S: 1.87 ± 2.85**	**S: 1.68 ± 2.42**	**A: 0.52 ± 1.04**	**A: 0.63 ± 1.25**	**NI: 0.22 ± 0.44**	
0.0625	31% ± 14.05%	16% ± 14.04%	41 ± 29.56%	37 ± 17.98%	23 ± 19.36%	17 ± 13.98%	17 ± 14.22%	20 ± 15.42%	3 ± 3.70	4±4.85%
	**S: 2.58 ± 3.27**	**A: 1.11 ± 1.49**	**S: 3.70 ± 5.42**	**S: 2.64 ± 3.28**	**A: 1.30 ± 2.29**	**A: 1.21 ± 1.50**	**A: 1.53 ± 2.33**	**A: 0.99 ± 1.99**	**NI: 0.11 ± 0.21**	
0.03,125	7% ± 13.06%	11% ± 15.38%	40 ± 37.52%	28 ± 23.74%	31 ± 26.26%	5 ± 9.70%	4 ± 7.59%	4 ± 3.87%	0±0.37%	0±0%
	**NI: 0 ± 0**	**NI: 0 ± 0**	**NI: 0 ± 0**	**NI: 0 ± 0**	**NI: 0 ± 0**	**NI: 0 ± 0**	**NI: 0 ± 0**	**NI: 0 ± 0**	**NI: 0 ± 0**	
0	0% ± 0%	0% ± 0%	0 ± 0%	0 ± 0.25%	0 ± 0.25%	0 ± 0%	0 ± 0.12%	0 ± 0%	1±2.61%	0±0%

^*a*^ DHEC concentrations are displayed in the columns and AMB concentrations in the rows. Inhibitory activity (IP, %) was determined following combination treatment with DHEC and AMB and represents percentage growth inhibition rather than residual growth. Interaction ratios (IRs), which were calculated using the Abbott formula, are indicated in bold below the corresponding IPs values. Grey cells and “S”, indicate synergistic interactions (IR > 1.5), “A”, additive effects (IR = 0.5–1.5), and “NI”, no interactions. Synergistic interactions indicate greater-than-expected inhibitory effects relative to the individual treatments and do not necessarily correspond to complete growth inhibition. Cells outlined in black represent approximately 100% growth inhibition.

Meanwhile, MECD exhibited a synergistic interaction only with AMB; however, none of the tested concentration ranges resulted in complete growth inhibition. An antagonistic interaction was also observed between MECD and 5-FC. Combinations featuring FLC or MCF predominantly exhibited no interaction, although synergistic or additive effects were detected at specific concentrations based on IR (Table S3).

### *In vivo* evaluation revealed no detectable toxicity but demonstrated preservation of the therapeutic activity of DHEC + AMB

**Fig 1:**
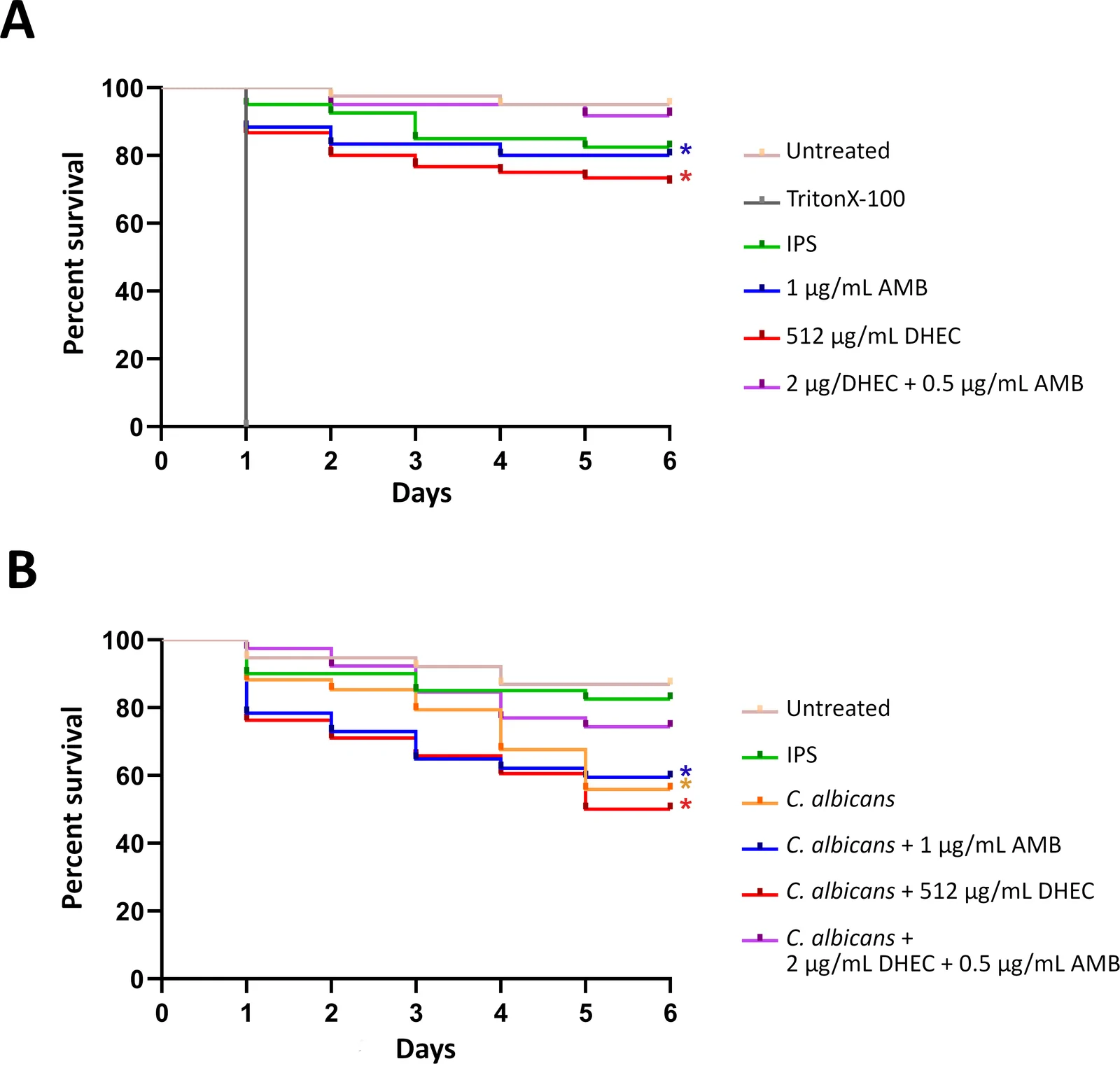
(**A**) *In vivo* toxicity of amphotericin B (AMB) and dihydroergocristine mesylate (DHEC), administered individually or in combination, in the *Galleria mellonella* Larval model. Larval survival (%) was recorded following treatment with 20 μL of insect physiological saline (IPS) containing 1 μg/mL AMB, 512 μg/mL DHEC, or 0.5 μg/mL AMB + 2 µg/mL DHEC. A survival rate of 100% corresponded to 40 individuals. Asterisks indicate statistically significant differences relative to the untreated control (*P* ≤ 0.05), as determined by both the log-rank (Mantel–Cox) and Gehan–Breslow–Wilcoxon tests. (**B**) The *in vivo* efficacy of AMB, DHEC, and their combination (AMB + DHEC) was evaluated in a *G. mellonella* infection model. Following infection with *C. albicans*, larvae were treated with 20 μL of 1 μg/mL AMB, 512 μg/mL DHEC, or their combination (0.5 μg/mL AMB + 2 µg/mL DHEC). A survival rate of 100% corresponds to 40 individuals. Asterisks indicate statistically significant differences relative to the untreated control (*P* ≤ 0.05), as determined by both the log-rank (Mantel–Cox) and Gehan–Breslow–Wilcoxon tests.

To evaluate the safety of combination therapy, the toxicity of DHEC and AMB, the most promising combination, was assessed using a *G. mellonella* larval model. The applied combination (2 μg/mL DHEC + 0.5 µg/mL AMB) did not significantly affect larval survival during the observation period compared with the findings in the untreated control (*P* = 0.5299). By contrast, treatment with DHEC (512 μg/mL) or AMB (1 μg/mL) alone resulted in a statistically significant reduction in larval survival vs the untreated control (*P* = 0.0037 and *P* = 0.0334, respectively; Fig. 1A; Table S4). However, these concentrations were higher than those used in the combination treatment. These findings indicate that the combination treatment did not exert detectable toxic effects under the applied experimental conditions.

The *in vivo* therapeutic potential of DHEC, AMB, and their combination was evaluated in *C. albicans*-infected *G. mellonella* larvae. Infection with *C. albicans* significantly reduced larval survival compared with that in the IPS-treated control (*P* = 0.0183). A similar reduction in survival was observed in larvae treated with DHEC or AMB alone (*P* = 0.0027, and *P* = 0.0273, respectively). Conversely, combined administration of DHEC and AMB did not result in a statistically significant reduction in survival relative to that in the IPS-treated control (*P* = 0.4311; Fig. 1B; Table S5). These results indicate that, unlike monotherapies, the combination of DHEC and AMB preserves host survival in the infected model, suggesting an improved therapeutic profile.

## DISCUSSION

The present study demonstrated that structure-based drug repurposing guided by NFAP2-defined fungal targets can identify compounds with measurable antifungal and adjuvant potential. However, a key observation was that the majority of computationally prioritized compounds failed to exhibit antifungal activity *in vitro* despite displaying strong predicted binding affinities (Table 1). This discrepancy highlights a well-recognized limitation of docking-based approaches, as predicted binding does not necessarily translate into biological efficacy because of factors such as limited cellular uptake, active efflux, metabolic instability, or insufficient target engagement under physiological conditions (27–29). In fungal systems, this issue is further exacerbated by the presence of a rigid cell wall and a membrane composition that can significantly restrict the intracellular access of small molecules (30). Thus, although NFAP2-associated binding pockets provide structurally meaningful targets, successful antifungal activity requires effective intracellular accumulation and functional interference with essential pathways alongside effective binding. In addition, the docking workflow relied on static protein structures and, therefore, did not account for protein dynamics, conformational flexibility, intracellular localization, or other cellular factors that may influence target accessibility and ligand binding. Consequently, docking scores should be interpreted primarily as indicators of potential target engagement rather than direct predictors of antifungal efficacy. Physicochemical properties may also contribute substantially to this discrepancy. Several highly ranked compounds, including venetoclax, glecaprevir, and simeprevir, possess high molecular weights and considerable structural complexity, characteristics that may impair penetration through the fungal cell wall and plasma membrane and thereby limit intracellular target engagement. By contrast, the biologically active compounds DHEC and MECD are comparatively smaller molecules with physicochemical properties potentially more favorable for cellular uptake.

Among the repurposed compounds, DHEC and MECD emerged as biologically active molecules with distinct activity profiles. Their antifungal effects can be interpreted in the context of their predicted interactions with NFAP2-associated targets. DHEC exhibited strong predicted binding to Gad1p and Atp1p, whereas MECD displayed a weaker and more selective interaction with Eno1p (Table 1). Gad1p is involved in amino acid metabolism and stress adaptation, whereas Atp1p plays a central role in energy production. Disruption of these processes can lead to rapid metabolic collapse and cell death, consistent with the central roles of mitochondrial function, metabolic homeostasis, and stress adaptation in determining antifungal susceptibility (31–33). By contrast, Eno1p, although essential for glycolysis, is often functionally redundant or buffered by metabolic plasticity, which might explain the weaker antifungal activity observed for MECD (34).

**Fig 2:**
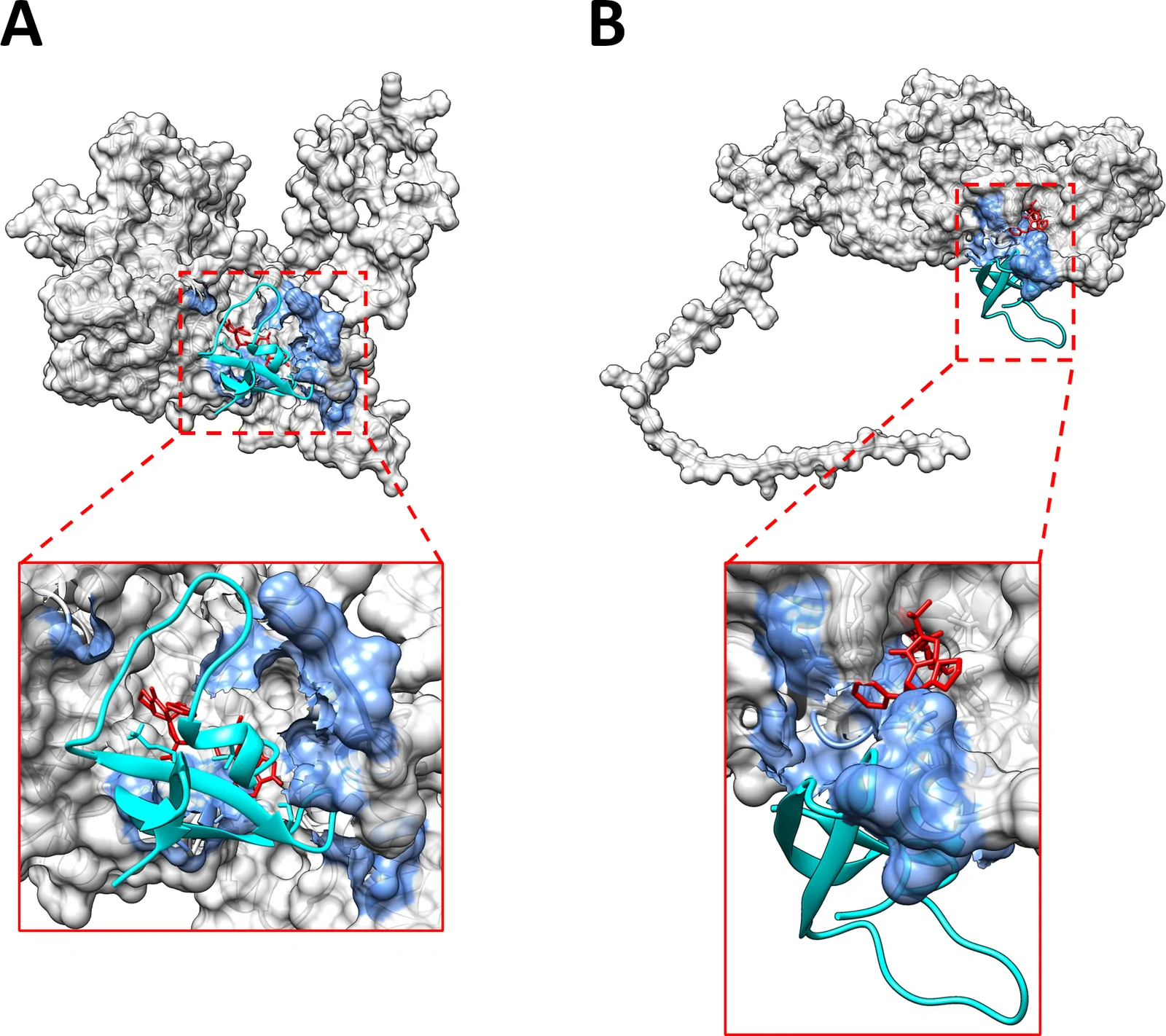
Structure-based docking of dihydroergocristine mesylate (DHEC) within the *Neosartorya* (currently *Aspergillus*) *fischeri* antifungal protein 2 (NFAP2)-defined binding regions of fungal targets. (**A**) Docking of DHEC into the NFAP2-defined binding site of *Candida albicans* glutamate decarboxylase (Gad1p). (**B**) Docking of DHEC into the corresponding NFAP2-associated binding region of mitochondrial *C. albicans* ATP synthase subunit alpha (Atp1p). In both panels, the target protein (Gad1p and Atp1p) structures are presented as gray surfaces. NFAP2 is depicted in cyan, with residues involved in binding highlighted in cornflower blue. The docked DHEC molecule is presented in red. The models were generated using the AutoDock Vina plugin implemented in UCSF Chimera and visualized in the same environment (22). For Gad1p, the best-ranked docking solution exhibited a predicted binding affinity of −9.4 kcal/mol, suggesting strong interaction with the target site, whereas for Atp1p, a representative docking pose (RMSD = 0.0 Å) was selected for structural interpretation. In both cases, docking results were interpreted in conjunction with structural complementarity rather than as absolute measures of binding strength.

Importantly, the strong predicted binding of DHEC to NFAP2-associated pockets in Gad1p and Atp1p, combined with the structural complementarity observed in docking models (Fig. 2), supports the hypothesis that DHEC might partially recapitulate the intracellular targeting component of NFAP2 activity. This is particularly relevant because NFAP2 exhibits a dual mode of action involving both membrane disruption and intracellular target engagement, a feature shared with several AFPs (18, 35). The ability of small molecules to mimic this intracellular targeting, even in the absence of membrane-disruptive activity, represents a promising strategy for antifungal drug development. In addition to their predicted interactions with NFAP2-associated intracellular targets, the known pharmacological modes of action of DHEC and MECD might further contribute to their antifungal effects through mechanisms that are independent of, yet potentially complementary to, NFAP2-mediated targeting.

DHEC is an ergot alkaloid derivative with well-characterized vasoactive and neuroactive properties, primarily acting by modulating dopaminergic and adrenergic receptors and targeting calcium homeostasis (36, 37). Ergot alkaloids have been reported to modulate intracellular Ca^2+^ homeostasis and redox processes, which are closely linked to mitochondrial function and cellular energy metabolism (38). These effects are consistent with the strong predicted binding of DHEC to Atp1p, a core component of the mitochondrial ATP synthase complex, suggesting that DHEC can impair fungal energy production through direct target engagement and intrinsic bioactivity. In parallel, interaction with Gad1p might disrupt amino acid metabolism and stress adaptation pathways, further amplifying metabolic imbalance. Together, these effects could contribute to the collapse of cellular homeostasis, resembling the intracellular component of NFAP2-mediated antifungal activity.

Contrarily, MECD is a first-generation antihistamine with known anticholinergic and membrane-active properties, and it has been reported to influence membrane fluidity and mitochondrial respiration in nonfungal systems (39, 40). Although MECD displayed only weak predicted binding to Eno1p, its antifungal activity under the modified assay conditions used in this study suggests the involvement of additional target-independent mechanisms. In particular, alterations in membrane properties could facilitate intracellular access or indirectly affect glycolytic flux, thereby sensitizing fungal cells to metabolic perturbations. This could explain the apparent discrepancy between its low predicted binding affinity and measurable biological activity.

Taken together, these observations indicate that the antifungal effects of DHEC and MECD likely arise from a combination of NFAP2-mimetic target engagement and intrinsic pharmacological activities. This dual contribution could be particularly relevant in the context of combination therapy, in which membrane perturbation, metabolic interference, and intracellular target inhibition can act synergistically to enhance antifungal efficacy.

Despite these mechanistic insights, the antifungal potency of both DHEC and MECD in susceptibility assays was limited, requiring relatively high concentrations to achieve growth inhibition. However, because susceptibility testing was performed using a modified CLSI-based protocol incorporating prolonged incubation and a stringent near-complete-growth-inhibition endpoint, the reported inhibitory concentrations should not be interpreted as CLSI-reference MIC values. For MECD, which exhibited the most consistent activity under the modified CLSI M27-A4-based assay conditions used in this study, the observed 50% growth inhibition at 256 μg/mL raises important pharmacokinetic considerations. Reported plasma concentrations of meclizine following oral administration (typically in the low ng/mL range) are several orders of magnitude lower than the concentrations required for *in vitro* antifungal activity, suggesting that its use as a standalone antifungal agent in invasive infections is unlikely (41). However, this limitation does not preclude its use as an adjuvant compound. Indeed, our results demonstrate that MECD exhibits a synergistic interaction with AMB, indicating that it could enhance antifungal efficacy at subtherapeutic concentrations (Table S3).

The observed synergistic interactions were strongly compound-dependent, with the most pronounced effects observed for DHEC (Tables 2; Table S1). It should be noted that mathematical synergy, as determined by the Abbott-based interaction ratio, does not necessarily imply complete growth inhibition. Accordingly, both the interaction ratio and the absolute inhibitory activity achieved by the combination were considered when evaluating the biological relevance of the observed interactions. Notably, DHEC was the only repurposed compound that, in combination with conventional antifungal agents, achieved near-complete growth inhibition under the tested *in vitro* conditions. This effect was particularly evident in combinations with AMB, indicating a highly effective cooperative interaction (Table 2). Therefore, DHEC should primarily be regarded as a proof-of-concept hit compound identified through the NFAP2-guided target-based repurposing workflow rather than as an immediately clinically applicable antifungal drug candidate.

Mechanistically, the pronounced synergy observed for DHEC likely arises from the combination of membrane-targeting and intracellular effects. AMB disrupts membrane integrity through ergosterol binding, thereby increasing membrane permeability (42), which might facilitate the intracellular accumulation of DHEC. In parallel, the predicted interactions of DHEC with Atp1p and Gad1p suggest interference with mitochondrial energy production and amino acid metabolism, respectively. Such simultaneous impairment of membrane integrity and core metabolic functions could lead to a rapid and irreversible loss of cellular homeostasis, explaining the near-complete growth inhibition observed *in vitro*. More broadly, these findings support a model in which the full antifungal activity of NFAP2 depends on both membrane perturbation and intracellular target inhibition. Notably, NFAP2 engages defined intracellular binding pockets, including structurally distinct interaction sites in Gad1p and Eno1p relative to their human homologs, which might contribute to its fungus-specific targeting (18). Within this framework, DHEC might reproduce mainly the intracellular component of NFAP2 activity, whereas cotreatment with AMB functionally restores the membrane-active component, thereby reconstituting an NFAP2-like dual targeting mechanism. A similar explanation might explain the strong interaction observed between DHEC and MCF, as weakening of the fungal cell wall could increase the susceptibility of fungal cells to intracellularly acting compounds. Consistent with this, disruption of mitochondrial function (Atp1p) and metabolic pathways (Gad1p) might sensitize fungal cells to membrane- or cell wall-targeting agents by reducing their capacity to maintain homeostasis and repair damage (31, 32, 43). This combined targeting of the fungal surface barrier and intracellular metabolism provides a plausible mechanistic basis for the strong antifungal synergy observed between these repurposed compounds and AMB or MCF (Tables 2; Tables S1 and S2).

Contrarily, although MECD displayed measurable antifungal activity and partial interactions, it did not achieve comparable levels of inhibition in combination regimens (Table S3). These findings indicate that pronounced *in vitro* synergy and near-complete growth suppression are specific to DHEC under the tested conditions, likely reflecting its ability to engage multiple intracellular vulnerability points.

The *in vivo* experiments further supported the translational relevance of the findings for DHEC. Notably, the DHEC + AMB combination exhibited no detectable toxicity in the *G. mellonella* model even though the individual compounds significantly reduced larval survival at higher concentrations. This suggests that the combination retained antifungal efficacy while reducing treatment-associated toxicity under the experimental conditions of the *G. mellonella* model (Fig. 1A). The *G. mellonella* experiments were designed to assess whether the synergistic interaction observed *in vitro* could be reproduced in a living host without detectable toxicity, rather than to establish clinical feasibility. Accordingly, the treatment concentrations were selected based on the most effective inhibitory and synergistic DHEC + AMB combinations identified during the *in vitro* susceptibility and checkerboard interaction analyses rather than through dose-optimization studies. Importantly, the concentrations used in the combination regimen (2 μg/mL DHEC + 0.5 µg/mL AMB) approach achievable ranges for AMB (42, 44). By contrast, the reported plasma levels of ergot derivatives, including DHEC, are typically in the low ng/mL range, suggesting that systemic availability is unlikely at the applied DHEC concentrations (45). Nevertheless, the absence of toxicity and preservation of host survival in the infected model indicate a favorable therapeutic profile. The improved survival observed in infected larvae treated with the DHEC + AMB combination compared with the effects of the monotherapies (Fig. 1B) suggests that synergistic targeting can mitigate both pathogen burden and treatment-associated toxicity. This is particularly significant, as AMB, although highly effective, is associated with dose-limiting toxicity in clinical settings (42). The ability to reduce the required dose through combination therapy could have substantial clinical benefits, including reduced adverse effects and potentially slower emergence of resistance (46, 47).

Taken together, our findings highlight the potential of NFAP2-guided drug repurposing as a strategy to identify compounds that exploit fungus-specific vulnerabilities. Although most repurposed compounds failed to demonstrate direct antifungal activity, select molecules, namely DHEC and MECD, produced important mechanistic insights, and synergistic potential. Therefore, DHEC should primarily be regarded as a proof-of-concept hit compound identified through the NFAP2-guided target-based repurposing workflow rather than as an immediately clinically applicable antifungal drug candidate. These results support the concept that AFP-defined intracellular targets can provide a foundation for the development of novel antifungal strategies, particularly in combination therapies. An additional limitation of the present study is that all experiments were performed using a single *C. albicans* reference strain (SC5314). Although the investigated NFAP2-associated targets (Gad1p, Atp1p, and Eno1p) are involved in essential cellular processes, including energy production, amino acid metabolism, stress adaptation, and glycolysis (31–34), natural genetic variation, differences in expression levels, or strain-specific physiological adaptations among clinical isolates may influence susceptibility to repurposed compounds targeting these proteins. Therefore, future studies should evaluate the reproducibility and generalizability of the observed interactions in genetically and phenotypically diverse clinical isolates, including antifungal-resistant strains.

In addition, future studies should focus on validating the identified NFAP2-associated targets *in vivo*, identifying pharmacokinetically more favorable compounds targeting the same fungal vulnerabilities, and developing optimized formulations or structural derivatives of promising hit compounds such as DHEC. Furthermore, the efficacy of optimized candidates should be evaluated in mammalian infection models.

Our results demonstrate that structure-based drug repurposing against pathogen-specific targets can identify candidate molecules, but predicted binding affinity alone does not reliably translate into antifungal activity. The observed discrepancies between *in silico* predictions and *in vitro* efficacy suggest that additional factors influence biological activity. Notably, the strongest effects were observed for combination treatments, indicating that repurposed compounds might be more effective as adjuvants rather than standalone agents. Overall, these findings emphasize the importance of integrating structure-based screening with experimental validation and early combination-based evaluation. Prioritizing multitarget engagement and combination strategies might improve the translational success of structure-based antifungal drug repurposing.

### Conclusion

This study demonstrates that NFAP2-guided, structure-based drug repurposing can identify compounds targeting fungus-specific intracellular vulnerabilities in *Candida albicans*. However, strong predicted binding does not necessarily translate into antifungal activity, underscoring the limitations of docking-based approaches when used in isolation. Instead, our results show that the most promising effects emerge in combination therapies, where repurposed compounds function as adjuvants rather than standalone agents. In particular, the combination of DHEC with AMB produced pronounced synergistic antifungal activity *in vitro* and improved survival without detectable toxicity *in vivo*. These findings support a model in which effective antifungal strategies arise from combined targeting of fungal membranes and intracellular pathways. Overall, integrating structure-based screening with experimental validation and early evaluation of combination effects may improve the translational success of antifungal drug repurposing.

## Supplementary Material

Reviewer comments

## Data Availability

All data supporting the findings of this study are included in the article and its supplemental material. Additional data are available from the corresponding author upon reasonable request.

## SUPPLEMENTAL MATERIAL

Tables S1 to S5; Fig. S1 and S2 spectrum.01310-26-s0001.pdf Table S1: Validation of the CLSI micafungin susceptibility of two independent *Candida albicans* SC5314 stocks determined under standard CLSI M27-A4 conditions (RPMI-1640 + MOPS, 35°C, 24 h). Table S2: Inhibitory activity and interaction analysis of dihydroergocristine mesylate (DHEC) and fluconazole (FLC), flucytosine (5-FC), and micafungin (MCF) combinations against *Candida albicans* SC5314. Table S3: Inhibitory activity and interaction analysis of meclizine dihydrochloride (MECD and amphotericin B (AMB), fluconazole (FLC), flucytosine (5-FC), and micafungin (MCF) combinations against *Candida albicans* SC5314. Table S4: Statistical analysis of toxicity data for amphotericin B (AMB), dihydroergocristine mesylate (DHEC), and their combination using GraphPad Prism 8. Table S5: Statistical analysis of the survival of *Galleria mellonella* larvae infected with *Candida albicans* SC5314 following treatment with amphotericin B (AMB), dihydroergocristine mesylate (DHEC), and their combination, performed using GraphPad Prism 8. Fig. S1: Chemical structures of candidate compounds with predicted affinity for the binding pockets of NFAP2. Fig. S2: Overview of compounds exhibiting strong predicted interactions with NFAP2-associated binding pockets in *Candida albicans*.

OPEN PEER REVIEW reviewer-comments.pdf An accounting of the reviewer comments and feedback.

ASM does not own the copyrights to Supplemental Material that may be linked to, or accessed through, an article. The authors have granted ASM a non-exclusive, world-wide license to publish the Supplemental Material files. Please contact the corresponding author directly for reuse.
