# Supplementary material for: Exploiting Neosartorya (Aspergillus) fischeri antifungal protein 2-defined fungal targets for drug repurposing against Candida albicans
Source: Microbiol Spectr. 2026 Oct 9;14(11):e01310-26. doi: 10.1128/spectrum.01310-26 (PMC13729217; doi:10.1128/spectrum.01310-26)
Supplement: Supplementary file 1 [file reviewer-comments.pdf]

ASM | Microbiology Spectrum

Exploiting Neosartorya (Aspergillus) fischeri antifungal protein 2-defined fungal targets for drug repurposing against Candida albicans

Kinga Dán, Rebeka Papp, Liliána Tóth, Lizett Miszlai, Gábor Bende, and László Galgóczy

Corresponding Author(s): László Galgóczy, Szegedi Tudomanyegyetem Termeszettudomanyi es Informatikai Kar

Review Timeline:

Submission Date:	April 15, 2026
Editorial Decision:	June 8, 2026
Revision Received:	June 23, 2026
Editorial Decision:	July 2, 2026
Revision Received:	July 22, 2026
Accepted:	August 3, 2026

Editor: Gregory Wiedman

Reviewer(s): Disclosure of reviewer identity is with reference to reviewer comments included in decision letter(s). The following individuals involved in review of your submission have agreed to reveal their identity: Feng Yang (Reviewer #1)

Transaction Report:

(Note: With the exception of the correction of typographical or spelling errors that could be a source of ambiguity, letters and reports are not edited. The original formatting of letters and referee reports may not be reflected in this compilation.)

DOI: https://doi.org/10.1128/spectrum.01310-26

Re: Spectrum01310-26 (Exploiting Neosartorya (Aspergillus) fischeri antifungal protein 2-defined fungal targets for drug repurposing against Candida albicans)

Dear Dr. László Galgóczy:

Thank you for the privilege of reviewing your work. Below you will find my comments, instructions from the Spectrum editorial office, and the reviewer comments.

Please return the manuscript within 60 days; if you cannot complete the modification within this time period, please contact me. If you do not wish to modify the manuscript and prefer to submit it to another journal, notify me immediately so that the manuscript may be formally withdrawn from consideration by Spectrum.

Revision Guidelines
To submit your modified manuscript, log into the submission site at https://spectrum.msubmit.net/cgi-bin/main.plex. Go to Author Tasks and click the appropriate manuscript title to begin. The information you entered when you first submitted the paper will be displayed; update this as necessary. Note the following requirements:

• Upload point-by-point responses to the issues raised by the reviewers in a file named "Response to Reviewers," NOT in your cover letter.
• Upload a compare copy of the manuscript (without figures) as a "Marked-Up Manuscript" file.
• Upload a clean .DOC/.DOCX version of the revised manuscript and remove the previous version.
• Each figure must be uploaded as a separate, editable, high-resolution file (TIFF or EPS preferred), and any multipanel figures must be assembled into one file.
• Any supplemental material intended for posting by ASM should be uploaded with their legends separate from the main manuscript. You can combine all supplemental material into one file (preferred) or split it into a maximum of 10 files with all associated legends included.

For complete guidelines on revision requirements, see our Submission and Review Process webpage. Submission of a paper that does not conform to guidelines may delay acceptance of your manuscript.

Data availability: ASM policy requires that data be available to the public upon online posting of the article, so please verify all links to sequence records, if present, and make sure that each number retrieves the full record of the data. If a new accession number is not linked or a link is broken, provide Spectrum production staff with the correct URL for the record. If the accession numbers for new data are not publicly accessible before the expected online posting of the article, publication may be delayed; please contact production staff (Spectrum@asmusa.org) immediately with the expected release date.

Publication Fees: For information on publication fees and which article types are subject to charges, visit our website. If your manuscript is accepted for publication and any fees apply, you will be contacted separately about payment during the production process; please follow the instructions in that e-mail. Arrangements for payment must be made before your article is published.

ASM Membership: Corresponding authors may join or renew ASM membership to obtain discounts on publication fees. Need to upgrade your membership level? Please contact Customer Service at Service@asmusa.org.

The ASM Journals program strives for constant improvement in our submission and publication process. Please tell us how we can improve your experience by taking this quick Author Survey.

Thank you for submitting your paper to Spectrum.

Sincerely,
Gregory Wiedman
Editor
Microbiology Spectrum

Reviewer #1 (Comments for the Author):

The development of novel antifungal drugs is notoriously slow and expensive, and the existing arsenal against invasive Candida albicans infections is limited by toxicity, drug interactions and emerging resistance. Drug repurposing offers an attractive shortcut, but its success depends on the rational selection of candidate molecules. In this study, the authors propose a structure-based repurposing strategy guided by the antifungal protein NFAP2, which previously was shown to bind to three

intracellular C. albicans proteins: glutamate decarboxylase (Gad1p), mitochondrial ATP synthase subunit alpha (Atp1p) and enolase (Eno1p). The authors first identified NFAP2-associated binding pockets on these targets and then performed virtual screening against a database of approved or clinically investigated compounds. Selected candidates were evaluated by molecular docking and subsequently tested in vitro for antifungal activity alone and in combination with conventional drugs. Finally, the most promising combination (dihydroergocristine mesylate, DHEC, with amphotericin B, AMB) was assessed for toxicity and therapeutic efficacy in a Galleria mellonella infection model. The main findings are that most computationally prioritised compounds lacked measurable antifungal activity, highlighting a disconnect between predicted binding and biological efficacy. Only DHEC and meclizine dihydrochloride showed clear antifungal effects, albeit at high concentrations. Importantly, DHEC exhibited strong synergistic interactions with AMB, fluconazole and micafungin in vitro, and the DHEC+AMB combination was well tolerated and significantly improved host survival in infected larvae compared with either drug alone.

The study addresses a genuinely important problem - the need for faster, cheaper antifungal drug discovery - and proposes an original approach by using a fungal protein (NFAP2) as a "blueprint" to identify druggable pockets on essential fungal targets. The integration of in silico screening, molecular docking, in vitro susceptibility testing, checkerboard synergy assays and an in vivo invertebrate model is commendable and provides a multi-level validation pipeline. The authors honestly report the disappointing observation that most predicted hits were inactive, which is a valuable cautionary message for the field. The discovery that DHEC synergises strongly with AMB (and to a lesser extent with other drug classes) is novel and potentially useful, especially because the combination allowed the use of lower, less toxic concentrations of AMB. The in vivo experiments are appropriately designed and the survival curves clearly support the benefit of the combination over monotherapies. The discussion is thoughtful, offering mechanistic explanations that link the predicted target engagement (Gad1p, Atp1p) to the observed synergy and to the known pharmacology of ergot alkaloids. Overall, the work is well written, logically structured and represents a solid contribution to the drug repurposing literature for fungal infections.

Despite the strengths mentioned above, I have several substantial concerns that need to be addressed before the manuscript can be accepted for publication.

1. The most serious issue relates to the determination of MICs for the conventional antifungal agents used in the synergy experiments. The authors report in the Results section that under CLSI M27-A4 conditions, the MIC of amphotericin B was 1 µg/mL, the MIC of flucytosine (5-FC) was 0.125 µg/mL, the MIC of micafungin (MCF) was 1 µg/mL, and the IC50 of fluconazole (FLC) was 0.5 µg/mL but its MIC was not obtained. These values are highly questionable. For a wild-type C. albicans reference strain such as SC5314, the expected MIC of flucytosine is typically {less than or equal to}0.5 µg/mL, so 0.125 µg/mL is not impossible, but the value for micafungin is problematic: most C. albicans isolates, including SC5314, have very low echinocandin MICs, usually {less than or equal to}0.03 µg/mL, and a MIC of 1 µg/mL would suggest either a technical error or possible contamination/resistance. The authors should verify their MCF stock solution, the dilution scheme and the incubation conditions. More strikingly, the failure to obtain a clear MIC for fluconazole (only an IC50 of 0.5 µg/mL) is highly unusual for C. albicans SC5314, which is fluconazole-susceptible with expected MICs of 0.25-1 µg/mL. That the authors could not determine a full MIC suggests that the growth conditions, inoculum preparation, medium composition or reading criteria were not properly controlled. Because these conventional drugs are used as anchors for the synergy analysis (Tables 2, S1, S2), unreliable MICs undermine the interpretation of the interaction ratios. The authors must repeat the susceptibility testing for AMB, FLC, 5-FC and MCF under strictly standardised CLSI conditions and report the actual MICs (not just IC50) before any conclusions about synergy can be trusted.

2. A second concern is the use of the Abbott formula-based interaction ratio (IR) instead of the standard fractional inhibitory concentration index (FICI). The authors justify this choice by stating that MICs for the repurposed compounds could not be reliably determined because of their limited activity. While this is a reasonable practical decision, the IR method is less commonly used in antifungal combination studies, and its interpretation (synergy >1.5, antagonism <0.5) is not as universally accepted as the FICI breakpoints. The authors should provide a reference for the IR cut-offs and discuss the potential limitations of this approach, especially when the individual inhibition values are low. In addition, many of the IR values reported in Table 2 for DHEC+AMB are extremely high (e.g., 4.64, 4.56) but correspond to only partial growth inhibition (e.g., 45% growth, 55% inhibition). The definition of synergy should not be based solely on a mathematical IR >1.5; it should also require that the combination effect is meaningfully greater than the effect of the most active single drug. The authors partially address this by stating that if the observed inhibition does not exceed that of the more active single component, the result is not considered a true interaction. However, this qualitative filter is not systematically applied in the tables, and some green-coded "synergistic" cells actually show modest inhibition (e.g., Table 2, DHEC 32 µg/mL + AMB 0.0625 µg/mL gives 41% growth, i.e. 59% inhibition, which is not "near-complete"). The presentation of synergy would benefit from a more conservative interpretation.

3. The in vitro antifungal activity of DHEC and meclizine was tested in two media (LCM and RPMI-1640). The authors correctly note that in RPMI-1640 DHEC showed no detectable activity (MIC >1024 µg/mL) and meclizine only 50% inhibition at 256 µg/mL. This means that under standardized conditions, the standalone activity of these repurposed compounds is essentially negligible. The synergy experiments for DHEC were also performed in RPMI-1640, but the concentrations of DHEC used in the checkerboard (128 µg/mL down to 0.5 µg/mL) are still orders of magnitude above any achievable plasma level for an ergot derivative (reported to be in the low ng/mL range). The authors themselves acknowledge that the systemic availability of DHEC at these concentrations is unlikely. This raises a serious question about the translational relevance of the in vitro synergy, even if it is statistically significant. The in vivo combination used 2 µg/mL DHEC (plus 0.5 µg/mL AMB) - still far above the ng/mL

range. Therefore, the claim that the combination allows "nontoxic concentrations" of AMB to be used is not supported by realistic pharmacokinetic data for DHEC. The authors should either provide evidence that DHEC can reach such concentrations in mammalian tissues (which they do not) or substantially moderate their translational claims.

4. The study is limited to a single strain of C. albicans (SC5314). Given that the whole approach relies on specific protein-ligand interactions, it is possible that natural genetic variation in Gad1p, Atp1p or Eno1p among clinical isolates could affect the binding and efficacy of DHEC or meclizine. The authors should at least discuss this limitation and ideally test a small panel of wild-type and fluconazole-resistant isolates to support the generalisability of their findings.

5. Some technical details are missing or unclear. For instance, the authors state that the MIC was defined as the lowest concentration that reduced fungal growth to {less than or equal to}5% of the untreated control. This is an unusually strict criterion (CLSI defines MIC as {greater than or equal to}50% or {greater than or equal to}90% inhibition for different drugs). The justification for using the 5% cut-off should be explained.

Minor comment:

Line 67. The year in which WHO issued the priority list should be mentioned.

Reviewer #2 (Comments for the Author):

Due to the limited number of antifungal classes and rising resistance rates, development of new antifungals or synergistic compounds that can be used in combination with existing antifungals is an urgent clinical need. The authors employ a promising and rigorous workflow to identify novel antifungals through the identification of pre-approved or pre-characterized drugs that can bind to NFAP2 targets. Interestingly, the authors' report synergistic killing of C. albicans with DHEC + amphotericin B both in vitro and within the Galleria larval model. However, the manuscript requires improved integration between computational and experimental findings and stronger justification of methodological choices. See specific comments below.

Comments:

Importantly, the authors point out the uncertain transition of computationally identified drugs to biological relevancy; although, this could be further explored. Can the authors provide a quantitative comparison between predicted binding energies and observed IC50/MIC values? Do any of the identified compounds exhibit physicochemical properties (e.g., polarity, molecular weight) that may limit fungal cell penetration? Does NFAP2 demonstrate conformational changes not captured by docking?

It would be helpful to see the actual MICs of the repurposed compounds against C. albicans. This could potentially be added to Table 1.

There are some procedural questions regarding the antifungal susceptibility testing. Why were the C. albicans MIC assays incubated at 30C instead of CLSI-recommended 35C or 37C? In addition, CLSI recommends reading MICs for amphotericin B, the echinocandins, and fluconazole at 24 hours if fungal growth is adequate. Echinocandin MICs should only be read at 24 hours according to CLSI recommendations. Was there limited growth at 24 hours?

Related to the above, the reported standalone micafungin MIC for C. albicans SC5314 is higher than anticipated. Based on clinical breakpoints established by CLSI and others, a micafungin MIC of 1 ug/ml is considered resistant. C. albicans SC5314 should exhibit susceptible MICs. This may be reflective of the authors' 48-hour reading whereas 24-hour readings are more appropriate and recommended. Do the authors have the 24-hour readings with micafungin and does this change any of the synergistic interpretations?

At which temperature did you incubate/store the Galleria before and after injections? Please add this information to the methods.

The authors should indicate statistical significance, when achieved, on relevant death curves in Figure 1. It is difficult to determine if the asterisks placed next to each treatment (text) are indicating statistical significance; they would be more noticeable if added to the actual curves at the relevant timepoint on the graph. Lastly, in the figure legends, please define or include the asterisk symbol in defining p value(s).

In the text, the authors state that the combo treatment did not significantly affect larval survival yet there is an asterisk next to "AMB + DHE" in figure 1a. Likewise, it is stated in the text that the combo treatment led to increased survival (no statistically significant killing) of infected larvae, yet there is an asterisk next to "C. albicans + AMB + DHEC" in figure 1b. Please address this.

The in vivo dosing rationale should be included. Why were those specific concentrations of AMP and DHEC chosen? The authors should run the Galleria infection models with varying concentrations of AMB, DHEC, and AMB + DHEC to observe expected dose responses, particularly with AMB and AMB + DHEC. This would better inform which concentrations exhibit in vivo efficacy (both as monotherapies and the combo therapy).

Minor comments:

Add some mention of future work or next step(s) related to DHEC and/or meclizine dihydrochloride and their promise as synergistic antifungals in the abstract or importance sections.

Line 75: use "and/or" instead of "and" when listing drawbacks of current antifungal classes as not all of those drawbacks listed are relevant to all antifungals.

Table 2: clarify on the table or in the description which range of drug corresponds to AmpB and which corresponds to DHEC.

Line 346: the abbreviation "DHE" is used instead of DHEC

Figure 1 also uses "DHE" instead of DHEC. The authors should be consistent with that abbreviation.

Lines 371-376: The functional descriptions of the NFAP2-associated targets may be better served in the introduction, even if briefly mentioned.

University of Szeged
Faculty of Science and Informatics
Department of Biotechnology and Microbiology
Szeged, Közép fasor 52
H-6726, HUNGARY
www2.bio.u-szeged.hu/galgoczylab

Mail: Szeged, Közép fasor 52
H-6726, HUNGARY
Phone: +36 62 546 936
Fax: +36 62 544 352
galgoczi@bio.u-szeged.hu

June 22, 2026

Dear Prof. Wiedman,

We sincerely thank the Editor and the Reviewers for their careful evaluation of our manuscript and for their constructive and insightful comments. We greatly appreciate the time and expertise invested in reviewing our work. The suggestions provided have significantly improved the quality, clarity, and scientific rigor of the manuscript.

In response to the reviewers' comments, we have thoroughly revised the manuscript. The revisions include clarification of the experimental methodology, improved presentation and interpretation of the interaction analyses, moderation of statements regarding translational applicability, expansion of the discussion of study limitations and future directions, and enhanced contextualization of the computational and biological findings. We have also revised the figures, tables, and supplementary materials where appropriate to improve clarity and consistency.

Below, we provide a detailed, point-by-point response to each comment and indicate the corresponding changes made in the revised manuscript.

Reviewer #1:

We sincerely thank the Reviewer for the thorough, constructive, and insightful evaluation of our manuscript. We greatly appreciate the positive assessment of the novelty and significance of our study. We are grateful for the Reviewer's detailed comments and suggestions, which have helped us to improve the clarity, rigor, and overall quality of the manuscript. All comments have been carefully considered, and the manuscript has been revised accordingly. Detailed point-by-point responses are provided below.

1. The most serious issue relates to the determination of MICs for the conventional antifungal agents used in the synergy experiments. The authors report in the Results section that under CLSI M27-A4 conditions, the MIC of amphotericin B was 1 µg/mL, the MIC of flucytosine (5-FC) was 0.125 µg/mL, the MIC of micafungin (MCF) was 1 µg/mL, and the IC50 of fluconazole (FLC) was 0.5 µg/mL but its MIC was not obtained. These values are highly questionable. For a wild-type C. albicans reference strain such as SC5314, the expected MIC of flucytosine is typically {less than or equal to}0.5 µg/mL, so 0.125 µg/mL is not impossible, but the value for micafungin is problematic: most C. albicans isolates, including SC5314, have very low echinocandin MICs, usually {less than or equal to}0.03 µg/mL, and a MIC of 1 µg/mL would suggest either a technical error or possible contamination/resistance. The authors should verify their MCF stock solution, the dilution scheme and the incubation conditions. More strikingly, the failure to obtain a clear MIC for fluconazole (only an IC50 of 0.5 µg/mL) is highly unusual for C. albicans SC5314, which is fluconazole-susceptible with expected MICs of 0.25-1 µg/mL. That the authors could not determine a full MIC suggests that the growth conditions, inoculum preparation,

medium composition or reading criteria were not properly controlled. Because these conventional drugs are used as anchors for the synergy analysis (Tables 2, S1, S2), unreliable MICs undermine the interpretation of the interaction ratios. The authors must repeat the susceptibility testing for AMB, FLC, 5-FC and MCF under strictly standardised CLSI conditions and report the actual MICs (not just IC50) before any conclusions about synergy can be trusted.

We thank the Reviewer for this important comment and agree that the susceptibility values reported in our study differ from the MIC values typically reported for *C. albicans* SC5314 under strictly standardized CLSI conditions.

Our objective, however, was not to establish clinical susceptibility breakpoints or generate CLSI-reference MIC values, but rather to evaluate the antifungal and adjuvant potential of repurposed compounds under a uniform experimental framework optimized for the subsequent interaction analyses. Therefore, although the assays were based on the CLSI M27-A4 methodology, several experimental parameters were intentionally modified, including incubation at 30°C, a 48-h incubation period, and a stringent endpoint definition requiring near-complete growth inhibition (≤5% residual growth relative to the untreated control). These conditions were selected to provide a more stringent assessment of sustained antifungal efficacy and long-term growth suppression, with the aim of identifying treatments capable of preventing delayed regrowth and minimizing the contribution of tolerant or persistent subpopulations during prolonged exposure, a feature that may be particularly relevant for reducing the risk of treatment failure and the emergence of adaptive tolerance.

We agree that these modifications can influence the apparent susceptibility values, particularly for echinocandins such as micafungin. Lower incubation temperature, prolonged incubation time, and OD-based endpoint determination are all known to increase apparent MIC values compared with standard CLSI measurements. Similarly, the absence of a clearly defined fluconazole MIC under our experimental conditions likely reflects the well-described phenomenon of azole trailing growth rather than intrinsic resistance of the SC5314 strain (Drug Resist Updat. 2015;23:12-19; doi: 10.1016/j.drup.2015.10.001). Trailing growth is characterized by persistent residual growth above the apparent MIC despite increasing fluconazole concentrations and becomes particularly evident during prolonged incubation periods (Drug Resist Updat. 2015;23:12-19; doi: 10.1016/j.drup.2015.10.001). Indeed, fluconazole-tolerant subpopulations have been reported to maintain growth above the MIC over a broad concentration range, resulting in residual growth that is largely insensitive to further increases in drug concentration (Nat Commun. 2018;9(1):2470; doi: 10.1038/s41467-018-04926-x). Consistent with this phenomenon, growth inhibition in our experiments did not reach the stringent MIC endpoint defined for this study, whereas IC_{50} values could still be reliably determined. Therefore, the absence of a fluconazole MIC should not be interpreted as evidence of reduced fluconazole susceptibility of the strain.

Importantly, all susceptibility and checkerboard interaction experiments were performed under identical conditions using the same inoculum preparation, medium composition, incubation parameters, and endpoint criteria. Therefore, although the obtained values should not be interpreted as CLSI-reference MICs, they provide an internally consistent framework for comparing the activities of the tested compounds and evaluating their combinatorial effects.

We also acknowledge that some sections of the original manuscript referred to the susceptibility assays as being performed under “standardized CLSI M27-A4 conditions.” In retrospect, this wording was imprecise and may have been misleading, as several assay parameters were intentionally modified relative to the standard CLSI protocol. To avoid confusion, the manuscript has been revised throughout

to consistently refer to these experiments as being conducted under modified CLSI-based conditions, and the corresponding methodological differences are now explicitly described.
To avoid potential confusion, we have revised the manuscript to clarify that the susceptibility assays were performed under modified CLSI-based conditions and that the reported inhibitory concentrations should be interpreted within the context of this experimental system rather than as clinical reference MIC values.

2. A second concern is the use of the Abbott formula-based interaction ratio (IR) instead of the standard fractional inhibitory concentration index (FICI). The authors justify this choice by stating that MICs for the repurposed compounds could not be reliably determined because of their limited activity. While this is a reasonable practical decision, the IR method is less commonly used in antifungal combination studies, and its interpretation (synergy >1.5, antagonism <0.5) is not as universally accepted as the FICI breakpoints. The authors should provide a reference for the IR cut-offs and discuss the potential limitations of this approach, especially when the individual inhibition values are low. In addition, many of the IR values reported in Table 2 for DHEC+AMB are extremely high (e.g., 4.64, 4.56) but correspond to only partial growth inhibition (e.g., 45% growth, 55% inhibition). The definition of synergy should not be based solely on a mathematical IR >1.5; it should also require that the combination effect is meaningfully greater than the effect of the most active single drug. The authors partially address this by stating that if the observed inhibition does not exceed that of the more active single component, the result is not considered a true interaction. However, this qualitative filter is not systematically applied in the tables, and some green-coded "synergistic" cells actually show modest inhibition (e.g., Table 2, DHEC 32 µg/mL + AMB 0.0625 µg/mL gives 41% growth, i.e. 59% inhibition, which is not "near-complete"). The presentation of synergy would benefit from a more conservative interpretation.

We thank the Reviewer for this thoughtful comment. We agree that the fractional inhibitory concentration index (FICI) is the most commonly applied method for antifungal interaction studies. However, FICI calculations require reliable MIC values for both compounds included in the combination. Since several repurposed compounds investigated in this study did not achieve the predefined MIC endpoint (≤5% residual growth relative to the untreated control) under the modified assay conditions used throughout the study, only IC50 values, but not MIC values, could be determined in a biologically meaningful manner. Consequently, FICI values could not be reliably calculated. For this reason, an effect-based interaction analysis was employed using the Abbott formula, which has previously been applied for the evaluation of interactions between antimicrobial compounds. The corresponding reference for the IR cut-offs has been added to the revised manuscript, and the limitations of this approach have been discussed more explicitly.
We agree that interaction ratios should not be interpreted independently of the absolute inhibitory effect achieved by the combination. Accordingly, the revised manuscript now emphasizes that mathematical synergy and biological relevance are not necessarily equivalent and places greater emphasis on combinations exhibiting both synergistic interaction ratios and high absolute inhibitory activity.
We would also like to clarify a potential misunderstanding regarding Table 2. The values reported in the table correspond to inhibitory activity percentages (IP, %) rather than residual growth percentages. Thus, the DHEC (32 µg/mL) + AMB (0.0625 µg/mL) combination cited by the Reviewer produced 41% growth inhibition rather than 41% growth. We apologize if the original table layout contributed to this misunderstanding. To avoid further confusion, the table footnote and the corresponding Results section have been revised to more explicitly indicate that the reported values represent inhibitory activity. In

addition, Tables 2, S1, and S2 have been revised to more clearly indicate the identities and concentration ranges of the compounds presented in the rows and columns.
Importantly, the DHEC (32 µg/mL) + AMB (0.0625 µg/mL) combination was not presented as producing near-complete growth inhibition. In Table 2, combinations resulting in approximately 100% growth inhibition were specifically highlighted by black outlines, whereas green shading was used solely to indicate synergistic interactions according to the predefined Abbott-based criteria. The primary objective of the interaction analysis was not restricted to identifying combinations that completely inhibited growth, but rather to determine whether synergistic interactions could be detected at submaximal concentrations where neither compound alone produced complete inhibition. In fact, interaction analyses are often most informative under such conditions, since complete growth inhibition can mask the contribution of the individual compounds and thereby limit the interpretation of pharmacological interactions. Therefore, combinations displaying moderate but significantly greater-than-expected inhibitory effects relative to the individual treatments were considered biologically relevant indicators of synergy, even when complete growth inhibition was not achieved.
Nevertheless, we agree that combinations producing both strong synergy and near-complete growth inhibition are of greatest therapeutic interest. Accordingly, the revised Discussion places particular emphasis on these interactions, especially the DHEC–AMB combinations that resulted in approximately complete growth inhibition.

3. The in vitro antifungal activity of DHEC and meclizine was tested in two media (LCM and RPMI-1640). The authors correctly note that in RPMI-1640 DHEC showed no detectable activity (MIC >1024 µg/mL) and meclizine only 50% inhibition at 256 µg/mL. This means that under standardized conditions, the standalone activity of these repurposed compounds is essentially negligible. The synergy experiments for DHEC were also performed in RPMI-1640, but the concentrations of DHEC used in the checkerboard (128 µg/mL down to 0.5 µg/mL) are still orders of magnitude above any achievable plasma level for an ergot derivative (reported to be in the low ng/mL range). The authors themselves acknowledge that the systemic availability of DHEC at these concentrations is unlikely. This raises a serious question about the translational relevance of the in vitro synergy, even if it is statistically significant. The in vivo combination used 2 µg/mL DHEC (plus 0.5 µg/mL AMB) - still far above the ng/mL range. Therefore, the claim that the combination allows "nontoxic concentrations" of AMB to be used is not supported by realistic pharmacokinetic data for DHEC. The authors should either provide evidence that DHEC can reach such concentrations in mammalian tissues (which they do not) or substantially moderate their translational claims.

We thank the Reviewer for this important comment and agree that the pharmacokinetic properties of DHEC represent a significant limitation with respect to its direct systemic application as an antifungal agent. As correctly noted by the Reviewer, reported plasma concentrations of ergot alkaloid derivatives are substantially lower than the concentrations at which DHEC exhibited antifungal or synergistic activity in our *in vitro* experiments. We therefore agree that the translational implications of these findings should be interpreted with caution.
Our primary objective was not to propose DHEC as an immediately clinically applicable antifungal drug, but rather to evaluate whether an NFAP2-guided target-based repurposing strategy could identify compounds capable of reproducing aspects of NFAP2-associated antifungal activity. From this perspective, DHEC should be regarded primarily as a proof-of-concept hit compound and a mechanistically informative lead identified through the target-guided screening workflow.

Importantly, the central conclusion of the study does not depend on the direct clinical applicability of DHEC. Rather, the findings demonstrate that NFAP2-associated fungal targets can be exploited for structure-based repurposing and that compounds identified through this approach can display measurable antifungal activity and synergistic interactions with conventional antifungal agents. We therefore agree that the pharmacokinetic limitations of DHEC restrict the immediate translational relevance of the molecule itself.

To address this concern, the Discussion has been revised to more clearly distinguish between the proof-of-concept validation of the repurposing strategy and the potential therapeutic applicability of the identified compounds. Statements suggesting direct clinical translation have been moderated, and additional text has been included emphasizing that further optimization, alternative formulations, structural derivatives, or identification of pharmacokinetically more favorable compounds targeting the same fungal vulnerabilities will be required before clinical application can be considered.

Regarding the *Galleria mellonella* experiments, our intention was not to demonstrate clinical feasibility, but rather to assess whether the observed combination effect could be reproduced in a living host without detectable toxicity. The corresponding sections of the manuscript have been revised to clarify this distinction.

4. The study is limited to a single strain of C. albicans (SC5314). Given that the whole approach relies on specific protein-ligand interactions, it is possible that natural genetic variation in Gad1p, Atp1p or Eno1p among clinical isolates could affect the binding and efficacy of DHEC or meclizine. The authors should at least discuss this limitation and ideally test a small panel of wild-type and fluconazole-resistant isolates to support the generalisability of their findings.

We agree that the use of a single *C. albicans* strain represents a limitation of the present study.

The primary objective of this work was to establish a proof-of-concept framework for NFAP2-guided target-based drug repurposing and to determine whether compounds identified through NFAP2-associated binding pockets could exhibit antifungal activity and interact with conventional antifungal agents. For this purpose, the well-characterized reference strain SC5314 was selected as a representative model system.

We agree that natural genetic variation among clinical isolates could potentially influence the efficacy of compounds targeting specific protein binding pockets. However, it should be noted that the three investigated NFAP2-associated targets (Gad1p, Atp1p, and Eno1p) are highly conserved proteins involved in essential cellular processes. Indeed, the identification of highly conserved intracellular targets was one of the reasons for selecting NFAP2-associated proteins as candidates for the repurposing workflow. Consequently, substantial variation affecting ligand binding is expected to be less frequent than for many classical resistance determinants. Nevertheless, we fully acknowledge that sequence polymorphisms, differences in protein expression, or strain-specific physiological adaptations may influence susceptibility to repurposed compounds identified through this approach.

We agree that evaluating a broader collection of clinical isolates, including fluconazole-resistant strains, would be valuable for assessing the generalizability of the findings. However, such experiments were beyond the scope of the present proof-of-concept study. To address this limitation, we have expanded the Discussion to explicitly acknowledge the potential impact of strain-to-strain variation and to emphasize the need for future validation using genetically and phenotypically diverse clinical isolates.

We thank the Reviewer for highlighting this important aspect and have revised the manuscript accordingly.

5. Some technical details are missing or unclear. For instance, the authors state that the MIC was defined as the lowest concentration that reduced fungal growth to {less than or equal to}5% of the untreated control. This is an unusually strict criterion (CLSI defines MIC as {greater than or equal to}50% or {greater than or equal to}90% inhibition for different drugs). The justification for using the 5% cut-off should be explained.

We agree that the MIC endpoint used in this study differs from the conventional CLSI MIC definitions. As noted in our response to comment 1, the primary objective of the susceptibility assays was not to establish clinical susceptibility breakpoints or determine CLSI-reference MIC values. Rather, the assays were designed to evaluate the antifungal and adjuvant potential of repurposed compounds under a uniform experimental framework that was subsequently used for the interaction analyses.
For this reason, a stringent endpoint was intentionally applied, defining the MIC as the lowest concentration resulting in ≤5% residual growth relative to the untreated control. This threshold was selected to ensure that residual growth remained within the range of experimental background variation associated with OD-based measurements and biological replicate-to-replicate variability, thereby providing a conservative estimate of near-complete growth inhibition. This criterion was selected to identify concentrations producing near-complete and sustained growth inhibition under the modified assay conditions used throughout the study. In contrast to conventional susceptibility testing, which is primarily intended for clinical categorization of isolates, our approach was designed to distinguish compounds capable of maintaining strong antifungal activity during prolonged exposure and to provide a consistent basis for subsequent combination studies.
We agree that this endpoint differs from standard CLSI definitions and may lead to susceptibility values that are not directly comparable to published CLSI MIC data. To avoid potential confusion, the revised manuscript now explicitly states that the reported MIC values were determined using a modified assay protocol and a more stringent endpoint definition than those used in standard CLSI susceptibility testing. We thank the Reviewer for highlighting this point and have clarified the rationale for the selected MIC endpoint in the Materials and Methods section.

Minor comment:
Line 67. The year in which WHO issued the priority list should be mentioned.

The text has been revised to refer explicitly to the WHO Fungal Priority Pathogens List published in 2022.

Reviewer #2:

We sincerely thank the Reviewer for the careful evaluation of our manuscript and for the constructive comments and suggestions. We greatly appreciate the positive assessment of the study and the recognition of the novelty of the NFAP2-guided drug repurposing approach. We are particularly grateful for the Reviewer's insightful remarks regarding the interpretation of the docking results, the presentation of the susceptibility and interaction data, and the discussion of the biological relevance of the identified compounds.
All comments have been carefully considered, and the manuscript has been revised accordingly. We believe that these revisions have improved the clarity, rigor, and overall quality of the manuscript. Detailed point-by-point responses are provided below.

1. Importantly, the authors point out the uncertain transition of computationally identified drugs to biological relevancy; although, this could be further explored. Can the authors provide a quantitative comparison between predicted binding energies and observed IC50/MIC values? Do any of the identified compounds exhibit physicochemical properties (e.g., polarity, molecular weight) that may limit fungal cell penetration? Does NFAP2 demonstrate conformational changes not captured by docking?

We thank the Reviewer for this insightful comment and agree that the relationship between predicted binding affinity and biological activity warrants further discussion.

While several compounds exhibited strong predicted binding to NFAP2-associated target pockets, only a subset demonstrated measurable antifungal activity *in vitro*. Accordingly, no apparent relationship was observed between predicted binding energies and experimentally determined antifungal activity in the present study. For example, several compounds exhibiting strong predicted interactions with NFAP2-associated targets failed to demonstrate measurable antifungal activity, whereas MECD displayed only weak predicted binding but retained detectable biological activity. These observations suggest that target binding alone is insufficient to predict antifungal efficacy.

As suggested by the Reviewer, physicochemical properties may contribute substantially to this discrepancy. Several of the computationally prioritized compounds, including venetoclax, glecaprevir, and simeprevir, possess high molecular weights and considerable structural complexity, which may limit penetration through the fungal cell wall and plasma membrane and thereby reduce intracellular target engagement. In contrast, the biologically active compounds DHEC and meclizine are comparatively smaller molecules with physicochemical properties potentially more favorable for cellular uptake.

We also agree that molecular docking represents a simplified model of protein–ligand interactions. The docking calculations were performed using static protein structures and therefore do not account for protein dynamics, conformational flexibility, intracellular localization, or other cellular factors that may influence target accessibility. Furthermore, the NFAP2-associated binding pockets used for screening were derived from previously identified NFAP2–target interaction sites and thus represent structural approximations of biologically relevant interaction regions. Consequently, docking scores were interpreted as indicators of potential target engagement rather than direct predictors of antifungal activity.

To address these important points, the Discussion has been expanded to more explicitly discuss the limitations of docking-based predictions, the potential role of physicochemical properties in fungal cell penetration, and the challenges associated with translating predicted binding affinity into biological efficacy.

2. It would be helpful to see the actual MICs of the repurposed compounds against C. albicans. This could potentially be added to Table 1.

We thank the Reviewer for this helpful suggestion. The experimentally determined MIC and/or IC_{50} values of the repurposed compounds have now been added to Table 1 to facilitate direct comparison between predicted binding affinities and observed antifungal activities.

3. There are some procedural questions regarding the antifungal susceptibility testing. Why were the C. albicans MIC assays incubated at 30C instead of CLSI-recommended 35C or 37C? In addition, CLSI recommends reading MICs for amphotericin B, the echinocandins, and fluconazole at 24 hours if fungal

growth is adequate. Echinocandin MICs should only be read at 24 hours according to CLSI recommendations. Was there limited growth at 24 hours?

We thank the Reviewer for this important question. As also noted in our response to Reviewer #1, the susceptibility assays performed in this study were based on, but not intended to strictly replicate, the CLSI M27-A4 protocol. Our objective was not to establish CLSI-reference MIC values, but rather to evaluate the antifungal and adjuvant potential of repurposed compounds under a uniform experimental framework used throughout the study.
Accordingly, susceptibility assays were performed at 30°C to maintain consistency with the NFAP2-related experiments and the subsequent interaction studies. In addition, all susceptibility measurements were evaluated after 48 h of incubation using a stringent endpoint definition (≤5% residual growth relative to the untreated control). No 24-h MIC readings were performed. The extended incubation period was intentionally selected to assess sustained antifungal activity and long-term growth suppression under the experimental conditions employed in this study.
To avoid confusion with standard CLSI susceptibility testing, the revised manuscript now more explicitly states that the reported inhibitory concentrations were determined using a modified CLSI-based assay protocol and should not be interpreted as CLSI-reference MIC values.

4. Related to the above, the reported standalone micafungin MIC for C. albicans SC5314 is higher than anticipated. Based on clinical breakpoints established by CLSI and others, a micafungin MIC of 1 ug/ml is considered resistant. C. albicans SC5314 should exhibit susceptible MICs. This may be reflective of the authors' 48-hour reading whereas 24-hour readings are more appropriate and recommended. Do the authors have the 24-hour readings with micafungin and does this change any of the synergistic interpretations?

We thank the Reviewer for this important observation and agree that the reported micafungin inhibitory concentration is higher than would be expected for *C. albicans* SC5314 under standard CLSI susceptibility testing conditions.
As discussed in our response to Reviewer #1, the susceptibility assays in this study were performed using a modified CLSI-based protocol that differed from the standard methodology in several aspects, including incubation at 30°C, a 48-h incubation period, and a stringent endpoint definition requiring ≤5% residual growth relative to the untreated control. We agree that these modifications, particularly the prolonged incubation period, may have contributed to the elevated inhibitory concentration observed for micafungin compared with values typically reported from 24-h CLSI measurements.
No 24-h readings were collected during the study; therefore, direct comparison with standard CLSI micafungin MIC values is not possible. However, all susceptibility and interaction experiments were performed under identical experimental conditions. Consequently, while the reported inhibitory concentrations should not be interpreted as CLSI-reference MIC values, the relative comparisons and interaction analyses remain internally consistent within the experimental framework used throughout the study.
To clarify this issue, the revised manuscript now more explicitly states that the reported inhibitory concentrations were determined under modified assay conditions and are not directly comparable to standard CLSI susceptibility values.

5. At which temperature did you incubate/store the Galleria before and after injections? Please add this information to the methods.

We thank the Reviewer for pointing out this missing detail. The manuscript already states that larvae were incubated at 37°C following injection. To further clarify the experimental conditions, we have now added that the larvae were also maintained overnight at 37°C prior to injection. The Materials and Methods section has been revised accordingly.

6. The authors should indicate statistical significance, when achieved, on relevant death curves in Figure 1. It is difficult to determine if the asterisks placed next to each treatment (text) are indicating statistical significance; they would be more noticeable if added to the actual curves at the relevant timepoint on the graph. Lastly, in the figure legends, please define or include the asterisk symbol in defining p value(s).

We thank the Reviewer for this helpful suggestion. The survival curves in Figure 1 have been revised to more clearly indicate statistically significant differences by placing the corresponding significance markers at the relevant time points on the curves. In addition, the figure legend has been updated to explicitly define the asterisk notation and the associated p-value thresholds.

7. In the text, the authors state that the combo treatment did not significantly affect larval survival yet there is an asterisk next to "AMB + DHE" in figure 1a. Likewise, it is stated in the text that the combo treatment led to increased survival (no statistically significant killing) of infected larvae, yet there is an asterisk next to "C. albicans + AMB + DHEC" in figure 1b. Please address this.

We thank the Reviewer for identifying this issue, and for bringing this error to our attention. Upon re-examination of the original data and figure files, we found that the asterisks next to the AMB + DHEC treatment in Fig. 1A and the *C. albicans* + AMB + DHEC treatment in Fig. 1B were introduced inadvertently during figure preparation. As correctly noted by the Reviewer, these treatments did not show statistically significant differences corresponding to the displayed significance markers, and the statements in the text are correct.
The figure has therefore been corrected by removing the erroneous asterisks. In addition, the figure legend has been revised to more clearly describe the statistical comparisons and significance criteria.

8. The in vivo dosing rationale should be included. Why were those specific concentrations of AMP and DHEC chosen? The authors should run the Galleria infection models with varying concentrations of AMB, DHEC, and AMB + DHEC to observe expected dose responses, particularly with AMB and AMB + DHEC. This would better inform which concentrations exhibit in vivo efficacy (both as monotherapies and the combo therapy).

We thank the Reviewer for this valuable suggestion and agree that dose–response studies would provide additional information regarding the *in vivo* efficacy and therapeutic window of the tested compounds.
The concentrations selected for the *Galleria mellonella* experiments were based on the *in vitro* susceptibility and interaction analyses. For monotherapy treatments, concentrations corresponding to the experimentally determined inhibitory concentrations of the individual compounds were used. For combination therapy, the concentrations were selected from the most effective DHEC–AMB combination identified in the checkerboard assays, which exhibited strong synergistic interaction and near-complete growth inhibition under the experimental conditions employed in this study.

The primary objective of the *in vivo* experiments was to provide proof-of-concept validation of the biological relevance of the observed *in vitro* interactions rather than to establish dose–response relationships or optimize therapeutic regimens. We agree that systematic dose–response analyses of AMB, DHEC, and their combinations would be valuable and represent an important direction for future studies. However, such experiments were beyond the scope of the present study.
To clarify the rationale for dose selection, additional information has been added to the Materials and Methods section and the Discussion.

Minor comments:
Add some mention of future work or next step(s) related to DHEC and/or meclizine dihydrochloride and their promise as synergistic antifungals in the abstract or importance sections.

The Abstract and Importance sections have been revised to briefly highlight future directions, including the optimization of pharmacokinetic properties, validation of the identified targets *in vivo*, and the evaluation of DHEC-, meclizine-, and related compounds as potential antifungal adjuvants in combination therapies.

Line 75: use "and/or" instead of "and" when listing drawbacks of current antifungal classes as not all of those drawbacks listed are relevant to all antifungals.

The text has been revised accordingly, and "and" has been replaced with "and/or" to more accurately reflect that the listed limitations do not necessarily apply to all currently available antifungal agents.

Table 2: clarify on the table or in the description which range of drug corresponds to AmpB and which corresponds to DHEC.

To improve clarity, Table 2 has been revised to explicitly indicate that DHEC concentrations (μg/mL) are shown in the columns and AMB concentrations (μg/mL) are shown in the rows. The table heading and footnote have been updated accordingly. For consistency and to avoid potential ambiguity, the corresponding combination tables (Tables S1 and S2) have also been revised using the same annotation format.

Line 346: the abbreviation "DHE" is used instead of DHEC
Figure 1 also uses "DHE" instead of DHEC. The authors should be consistent with that abbreviation.

Te abbreviation has been corrected throughout the manuscript, and all occurrences of "DHE" have been replaced with the correct abbreviation "DHEC".

Lines 371-376: The functional descriptions of the NFAP2-associated targets may be better served in the introduction, even if briefly mentioned.

We agree that briefly introducing the biological functions of the NFAP2-associated target proteins earlier in the manuscript improves readability and provides better context for the subsequent docking and repurposing analyses. Accordingly, concise descriptions of Gad1p, Atp1p, and Eno1p have been added to the Introduction, indicating their roles in amino acid metabolism and stress adaptation,

mitochondrial ATP production, and glycolysis, respectively. These additions provide biological context for the target-selection strategy before the docking and repurposing analyses are presented.

Sincerely,

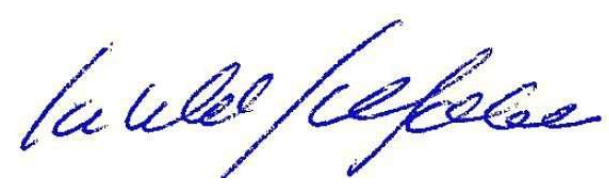

László Galgóczy, PhD, DSc
Associate Professor
Department of Biotechnology and Microbiology
Faculty of Science and Informatics, University of Szeged
Szeged, Hungary
Email: galgoczi@bio.u-szeged.hu

Re: Spectrum01310-26R1 (Exploiting Neosartorya (Aspergillus) fischeri antifungal protein 2-defined fungal targets for drug repurposing against Candida albicans)

Dear Dr. László Galgóczy:

Thank you for the privilege of reviewing your work. Below you will find my comments, instructions from the Spectrum editorial office, and the reviewer comments.

Please return the manuscript within 60 days; if you cannot complete the modification within this time period, please contact me. If you do not wish to modify the manuscript and prefer to submit it to another journal, notify me immediately so that the manuscript may be formally withdrawn from consideration by Spectrum.

Revision Guidelines
To submit your modified manuscript, log into the submission site at https://spectrum.msubmit.net/cgi-bin/main.plex. Go to Author Tasks and click the appropriate manuscript title to begin. The information you entered when you first submitted the paper will be displayed; update this as necessary. Note the following requirements:

• Upload point-by-point responses to the issues raised by the reviewers in a file named "Response to Reviewers," NOT in your cover letter.
• Upload a compare copy of the manuscript (without figures) as a "Marked-Up Manuscript" file.
• Upload a clean .DOC/.DOCX version of the revised manuscript and remove the previous version.
• Each figure must be uploaded as a separate, editable, high-resolution file (TIFF or EPS preferred), and any multipanel figures must be assembled into one file.
• Any supplemental material intended for posting by ASM should be uploaded with their legends separate from the main manuscript. You can combine all supplemental material into one file (preferred) or split it into a maximum of 10 files with all associated legends included.

For complete guidelines on revision requirements, see our Submission and Review Process webpage. Submission of a paper that does not conform to guidelines may delay acceptance of your manuscript.

Data availability: ASM policy requires that data be available to the public upon online posting of the article, so please verify all links to sequence records, if present, and make sure that each number retrieves the full record of the data. If a new accession number is not linked or a link is broken, provide Spectrum production staff with the correct URL for the record. If the accession numbers for new data are not publicly accessible before the expected online posting of the article, publication may be delayed; please contact production staff (Spectrum@asmusa.org) immediately with the expected release date.

Publication Fees: For information on publication fees and which article types are subject to charges, visit our website. If your manuscript is accepted for publication and any fees apply, you will be contacted separately about payment during the production process; please follow the instructions in that e-mail. Arrangements for payment must be made before your article is published.

ASM Membership: Corresponding authors may join or renew ASM membership to obtain discounts on publication fees. Need to upgrade your membership level? Please contact Customer Service at Service@asmusa.org.

The ASM Journals program strives for constant improvement in our submission and publication process. Please tell us how we can improve your experience by taking this quick Author Survey.

Thank you for submitting your paper to Spectrum.

Sincerely,
Gregory Wiedman
Editor
Microbiology Spectrum

Reviewer #1 (Comments for the Author):

All my concerns have been addressed.

Reviewer #2 (Comments for the Author):

The authors have addressed most points and made sufficient edits within the manuscript. However, I am still uncertain how the wild type exhibited a micafungin MIC of 1 ug/ml. Even at 48 hours and with slight adjustments to the CLSI protocol, the MIC should remain at least 8-fold lower than this. This elevated MIC likely invalidates, or at least skews, any data acquired with this drug by itself and in combination with the other compounds. Can the authors confirm this is in fact a wild-type strain (and lacks any fks1 mutation or strain contamination)? The drug stock can also be validated by obtaining 24 and 48h MICs of control strains of yeast.

Relatedly, I understand the authors' desire for "stringent" settings, but the results would be more comparable and reliable if MICs, both standalone and combinatorial, were measured at 24 hours. 48-hour MICs could be reported, if useful, in addition to the 24-hour measurements.

University of Szeged
Faculty of Science and Informatics
Department of Biotechnology and Microbiology
Szeged, Közép fasor 52
H-6726, HUNGARY
www2.bio.u-szeged.hu/galgoczylab

Mail: Szeged, Közép fasor 52
H-6726, HUNGARY
Phone: +36 62 546 936
Fax: +36 62 544 352
galgoczi@bio.u-szeged.hu

July 22, 2026

Dear Prof. Wiedman,

We sincerely thank you and the Reviewer for your careful evaluation of the revised version of our manuscript and for the constructive comments. We greatly appreciate the opportunity to further strengthen our study.
In response to the remaining comment, we performed additional validation experiments under standard CLSI M27-A4 conditions to verify the micafungin susceptibility of the *Candida albicans* SC5314 strain used throughout this study. Based on these new data, we have expanded the Materials and Methods section and included the corresponding validation results as Supplementary Table S1.
Below, we provide our detailed point-by-point response to the remaining comment and indicate the corresponding revisions made in the manuscript.

Reviewer #2:
The authors have addressed most points and made sufficient edits within the manuscript. However, I am still uncertain how the wild type exhibited a micafungin MIC of 1 ug/ml. Even at 48 hours and with slight adjustments to the CLSI protocol, the MIC should remain at least 8-fold lower than this. This elevated MIC likely invalidates, or at least skews, any data acquired with this drug by itself and in combination with the other compounds. Can the authors confirm this is in fact a wild-type strain (and lacks any fks1 mutation or strain contamination)? The drug stock can also be validated by obtaining 24 and 48h MICs of control strains of yeast.
Relatedly, I understand the authors' desire for "stringent" settings, but the results would be more comparable and reliable if MICs, both standalone and combinatorial, were measured at 24 hours. 48-hour MICs could be reported, if useful, in addition to the 24-hour measurements.

We sincerely thank the Reviewer for this important suggestion. To directly address this concern, we repeated the susceptibility testing under standard CLSI M27-A4 conditions using RPMI-1640 supplemented with MOPS, incubation at 35°C, a 24-h endpoint, and the conventional ≥50% growth inhibition criterion. In addition, to exclude the possibility of strain contamination or laboratory-specific adaptation, the experiments were independently performed using two *Candida albicans* SC5314 stocks: one maintained in our laboratory and a second obtained from the Host–Pathogen Interaction Centre (HPIC, University of Szeged).
Importantly, the validation experiments were performed using the same lot of commercially supplied micafungin as in the original study, which had been stored and handled according to the recommendations of the manufacturer. Under these standard CLSI conditions, both SC5314 stocks exhibited identical micafungin MIC values of 0.125 µg/mL (Table R1A, Table R2A, Figure R1A and Figure R2A), fully consistent with the CLSI susceptibility breakpoint for *C. albicans* (≤0.25 µg/mL).

These results strongly support that the strain used throughout this study corresponds to a wild-type SC5314 isolate with the expected micafungin susceptibility phenotype and exclude laboratory-specific strain contamination as an explanation for the previously observed results, and confirm that the micafungin preparation retained its expected biological activity. Furthermore, the CLSI susceptibility profile makes the presence of an FKS1-mediated resistance phenotype highly unlikely.

Following the Reviewer's recommendation, the same experiments were subsequently continued to 48 h at 35°C (Table R1B, Table R2B, Figure R1B and Figure R2B). In addition, microscopic examination was performed. Under these conditions, near-complete growth inhibition was observed only at micafungin concentrations of approximately 0.5–1 µg/mL (Fig. R3B and C), consistent with the stringent endpoint applied in our original study. Microscopic examination further revealed persistent filamentous growth at 0.25 µg/mL despite the reduced optical density signal, indicating that hyphal morphology and prolonged incubation substantially influence endpoint determination (Fig. R3A). Importantly, these validation experiments demonstrate that the elevated micafungin concentration reported under our modified assay conditions does not reflect intrinsic micafungin resistance, strain contamination, or loss of drug potency, but rather reflects the different experimental endpoint applied after prolonged incubation. These observations explain why inhibitory concentrations obtained under our modified assay conditions, which employed prolonged incubation and a stringent near-complete growth inhibition endpoint, are not directly comparable to standard CLSI MIC values determined after 24 h using the conventional 50% growth inhibition endpoint.

Accordingly, we have expanded the Materials and Methods section of the revised manuscript to describe the additional validation procedure, including the use of two independent SC5314 stocks and the experimental design. To ensure full transparency, the corresponding validation data have been included in the Supplemental Material (Table S1), while the main Results section remains focused on the primary objectives of the study.

Sincerely,

László Galgóczy, PhD, DSc
Associate Professor
Department of Biotechnology and Microbiology
Faculty of Science and Informatics, University of Szeged
Szeged, Hungary
Email: galgoczi@bio.u-szeged.hu

Tables for review

Table R1 Susceptibility of *Candida albicans* SC5314 stock from our laboratory against micafungin in RPMI-1640 + MOPS medium, after 24 h (A) and 48 h (B) incubation at 35°C following CLSI M27-A4 guidelines

A) **C. a. SC5314**

24h OD_{620}

µg/mL		8	4	2	1	0.5	0.25	0.125	0.063	0.031	0.016	0.008	GC
BR 1	TR1	0	0	0.001	0	-0.005	-0.001	0.018	0.294	0.276	0.272	0.296	0.301
	TR2	0	-0.001	-0.002	-0.002	-0.002	-0.001	0.015	0.26	0.247	0.257	0.238	0.236
	TR3	-0.001	-0.001	-0.002	0	-0.002	-0.003	0.022	0.289	0.309	0.324	0.314	0.281
BR 2	TR1	-0.001	0.007	-0.002	-0.004	0.005	0	0.013	0.259	0.241	0.24	0.254	0.206
	TR2	0	0.001	0	-0.002	0.008	-0.001	0.011	0.276	0.265	0.278	0.292	0.257
	TR3	-0.003	-0.002	0	0	0	-0.001	0.012	0.258	0.31	0.314	0.316	0.298

Growth rate %

µg/mL		8	4	2	1	0.5	0.25	0.125	0.063	0.031	0.016	0.008	GC
BR 1	TR1	0%	0%	0%	0%	-2%	0%	6%	98%	92%	90%	98%	100%
	TR2	0%	0%	-1%	-1%	-1%	0%	6%	110%	105%	109%	101%	100%
	TR3	0%	0%	-1%	0%	-1%	-1%	8%	103%	110%	115%	112%	100%
BR 2	TR1	0%	3%	-1%	-2%	2%	0%	6%	126%	117%	117%	123%	100%
	TR2	0%	0%	0%	-1%	3%	0%	4%	107%	103%	108%	114%	100%
	TR3	-1%	-1%	0%	0%	0%	0%	4%	87%	104%	105%	106%	100%
	Mean	0%	0%	0%	-1%	0%	0%	6%	105%	105%	107%	109%	100%
	Standard deviation	0%	1%	0%	1%	2%	0%	1%	12%	8%	9%	8%	0%

B) **C. a. SC5314**

48h OD_{620}

µg/mL		8	4	2	1	0.5	0.25	0.125	0.063	0.031	0.016	0.008	GC
BR 1	TR1	0	0	-0.002	-0.005	-0.008	-0.002	0.158	0.253	0.255	0.262	0.273	0.203
	TR2	0	0	-0.005	0.009	-0.007	-0.005	0.137	0.251	0.266	0.253	0.258	0.188
	TR3	-0.001	-0.001	-0.004	-0.002	-0.005	-0.005	0.137	0.279	0.288	0.298	0.29	0.218
BR 2	TR1	-0.005	-0.004	0.006	0.002	0.008	-0.001	0.017	0.237	0.23	0.238	0.225	0.151
	TR2	-0.004	-0.006	0.004	-0.001	0.005	0.001	0.024	0.249	0.26	0.275	0.268	0.207
	TR3	-0.006	-0.005	-0.003	-0.002	-0.004	0.001	0.027	0.238	0.273	0.274	0.3	0.199

Growth rate %

µg/mL		8	4	2	1	0.5	0.25	0.125	0.063	0.031	0.016	0.008	GC
BR 1	TR1	0%	0%	-1%	-2%	-4%	-1%	78%	125%	126%	129%	134%	100%
	TR2	0%	0%	-3%	5%	-4%	-3%	73%	134%	141%	135%	137%	100%
	TR3	0%	0%	-2%	-1%	-2%	-2%	63%	128%	132%	137%	133%	100%
BR 2	TR1	-3%	-3%	4%	1%	5%	-1%	11%	157%	152%	158%	149%	100%
	TR2	-2%	-3%	2%	0%	2%	0%	12%	120%	126%	133%	129%	100%
	TR3	-3%	-3%	-2%	-1%	-2%	1%	14%	120%	137%	138%	151%	100%
	Mean	-1%	-1%	0%	0%	-1%	-1%	42%	130%	136%	138%	139%	100%
	Standard deviation	1%	1%	2%	2%	3%	1%	30%	13%	9%	9%	8%	0%

BR: Biological replicate; *C. a.* SC5314: *Candida albicans* SC5314 stock from our laboratory; GC: growth control; Growth rate % = (Sample OD_{620} value / GC OD_{620} value) × 100; OD_{620}: optical density measured at 620 nanometer wavelength; TR: technical replicate.

Table R2 Susceptibility of *Candida albicans* SC5314 stock from the Host-Pathogen Interaction Centre against micafungin in RPMI-1640 + MOPS medium, after 24 h (A) and 48 h (B) incubation at 35°C following CLSI M27-A4 guidelines

A) C. a. SC5314 (HPIC)

24h OD_{620}

µg/mL		8	4	2	1	0.5	0.25	0.125	0.063	0.031	0.016	0.008	GC
BR 1	TR1	0.002	0.002	-0.001	0	0	-0.002	0.016	0.236	0.23	0.237	0.254	0.23
	TR2	-0.001	-0.001	-0.002	0.001	-0.001	-0.002	0.007	0.265	0.227	0.28	0.255	0.219
	TR3	-0.001	-0.001	-0.002	0.002	0.001	0.003	0.013	0.289	0.271	0.333	0.317	0.291
BR 2	TR1	-0.002	-0.003	-0.002	0.001	0.004	0.01	0.006	0.168	0.193	0.21	0.253	0.238
	TR2	-0.002	0.001	0.001	0	-0.001	0.001	0.005	0.208	0.226	0.21	0.204	0.234
	TR3	-0.001	0	0.001	0	0.001	-0.001	0.007	0.239	0.258	0.238	0.24	0.269

Growth rate %

µg/mL		8	4	2	1	0.5	0.25	0.125	0.063	0.031	0.016	0.008	GC
BR 1	TR1	1%	1%	0%	0%	0%	-1%	7%	103%	100%	103%	110%	100%
	TR2	0%	0%	-1%	0%	0%	-1%	3%	121%	104%	128%	116%	100%
	TR3	0%	0%	-1%	1%	0%	1%	4%	99%	93%	114%	109%	100%
BR 2	TR1	-1%	-1%	-1%	0%	2%	4%	3%	71%	81%	88%	106%	100%
	TR2	-1%	0%	0%	0%	0%	0%	2%	89%	97%	90%	87%	100%
	TR3	0%	0%	0%	0%	0%	0%	3%	89%	96%	88%	89%	100%
	Mean	0%	0%	0%	0%	0%	1%	4%	95%	95%	102%	103%	100%
	Standard deviation	1%	1%	1%	0%	1%	2%	2%	15%	7%	15%	11%	0%

B) C. a. SC5314 (HPIC)

48h OD_{620}

µg/mL		8	4	2	1	0.5	0.25	0.125	0.063	0.031	0.016	0.008	GC
BR 1	TR1	0	0.002	0.004	0.002	-0.007	0	0.057	0.217	0.207	0.214	0.282	0.162
	TR2	0.001	-0.002	-0.001	-0.001	-0.006	-0.003	0.034	0.252	0.196	0.233	0.242	0.177
	TR3	-0.008	-0.007	-0.005	-0.004	-0.006	-0.003	0.052	0.243	0.246	0.282	0.281	0.218
BR 2	TR1	-0.009	-0.004	-0.004	-0.005	-0.005	-0.003	0.01	0.163	0.134	0.18	0.241	0.182
	TR2	-0.002	-0.009	-0.007	-0.006	-0.004	-0.004	0.012	0.204	0.196	0.223	0.246	0.203
	TR3	-0.007	-0.008	-0.006	-0.005	-0.001	-0.003	0.024	0.222	0.223	0.261	0.248	0.249

Growth rate %

µg/mL		8	4	2	1	0.5	0.25	0.125	0.063	0.031	0.016	0.008	GC
BR 1	TR1	0%	1%	2%	1%	-4%	0%	35%	134%	128%	132%	174%	100%
	TR2	1%	-1%	-1%	-1%	-3%	-2%	19%	142%	111%	132%	137%	100%
	TR3	-4%	-3%	-2%	-2%	-3%	-1%	24%	111%	113%	129%	129%	100%
BR 2	TR1	-5%	-2%	-2%	-3%	-3%	-2%	5%	90%	74%	99%	132%	100%
	TR2	-1%	-4%	-3%	-3%	-2%	-2%	6%	100%	97%	110%	121%	100%
	TR3	-3%	-3%	-2%	-2%	0%	-1%	10%	89%	90%	105%	100%	100%
	Mean	-2%	-2%	-1%	-1%	-3%	-1%	17%	111%	102%	118%	132%	100%
	Standard deviation	2%	2%	2%	1%	1%	1%	11%	21%	18%	14%	22%	0%

BR: Biological replicate; *C. a.* SC5314 (HPIC): *Candida albicans* SC5314 stock from the Host-Pathogen Interaction Centre; GC: growth control; Growth rate % = (Sample OD_{620} value / GC OD_{620} value) × 100; OD_{620}: optical density measured at 620 nanometer wavelength; TR: technical replicate.

Figures for review

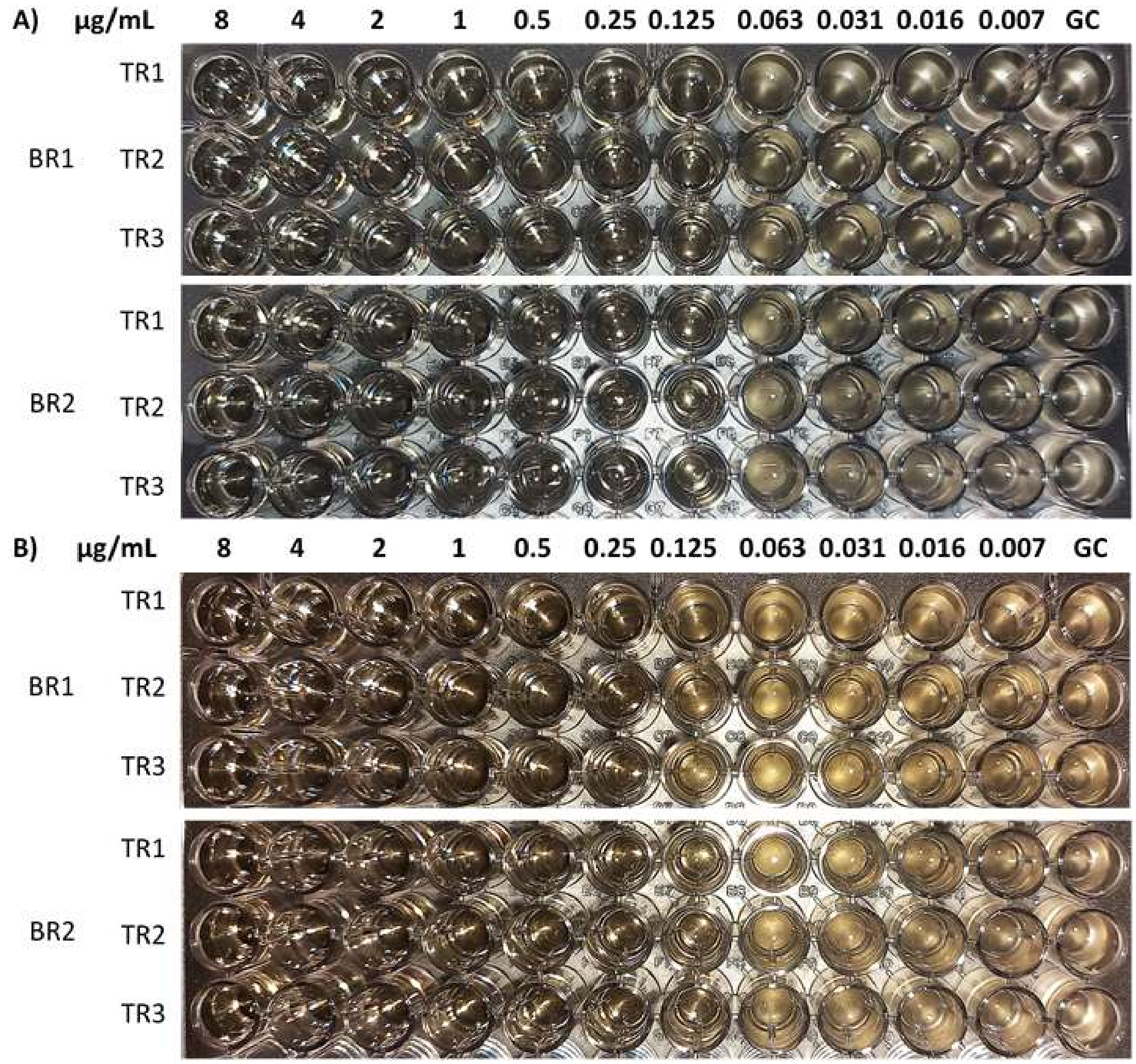

Fig R1 Photographic documentation of the microtiter plates from the micafungin susceptibility test of *Candida albicans* SC5314 stock from our laboratory. The cultures were in RPMI-1640 + MOPS medium, incubated for 24 h (A) and 48 h (B) at 35°C following CLSI M27-A4 guidelines. BR: biological replicate; TR: technical replicate.

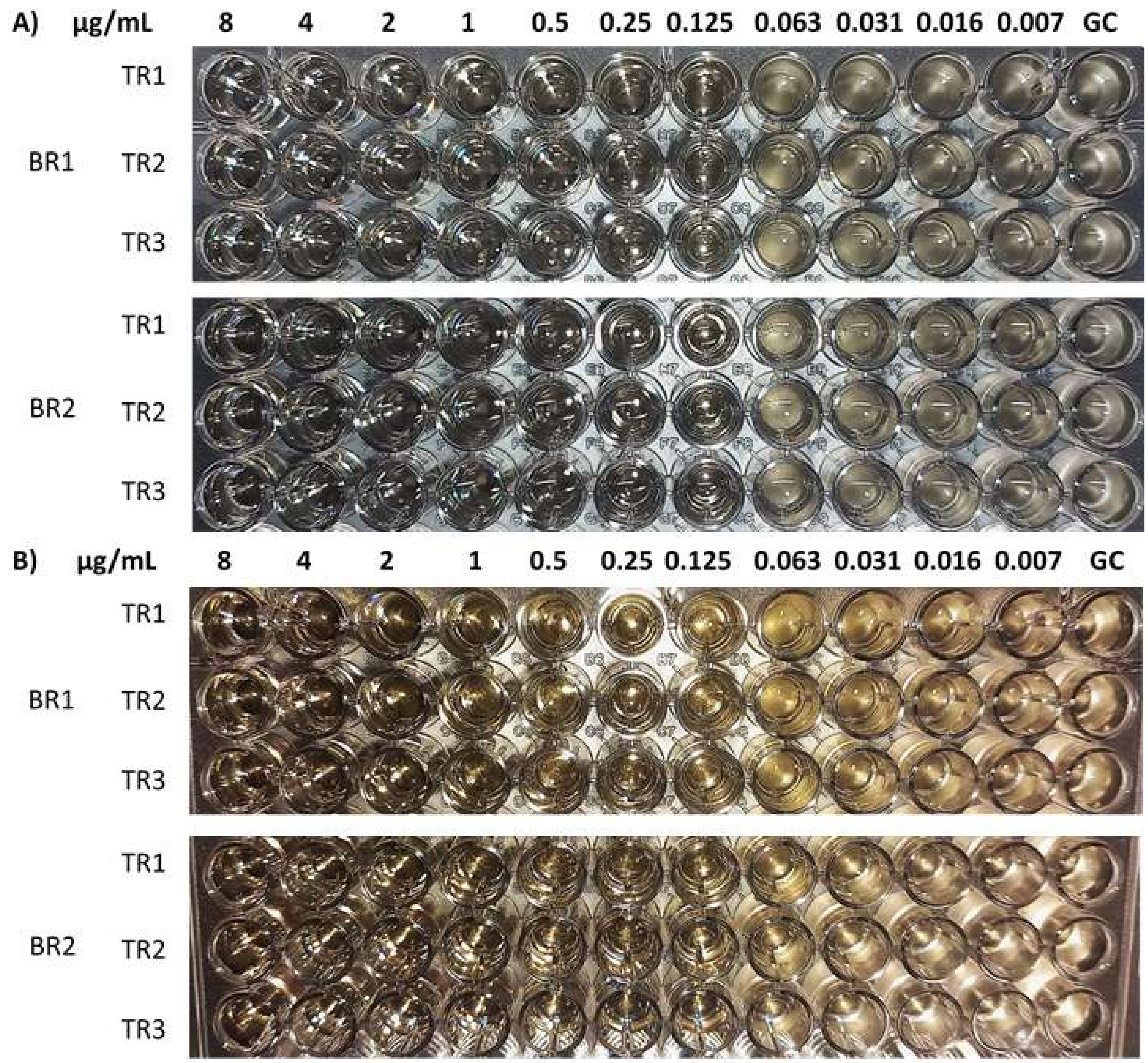

Fig R2 Photographic documentation of the microtiter plates from the micafungin susceptibility test of *Candida albicans* SC5314 stock from the Host-Pathogen Interaction Centre. The cultures were in RPMI-1640 + MOPS medium, incubated for 24 h (A) and 48 h (B) at 35°C following CLSI M27-A4 guidelines. BR: biological replicate; TR: technical replicate.

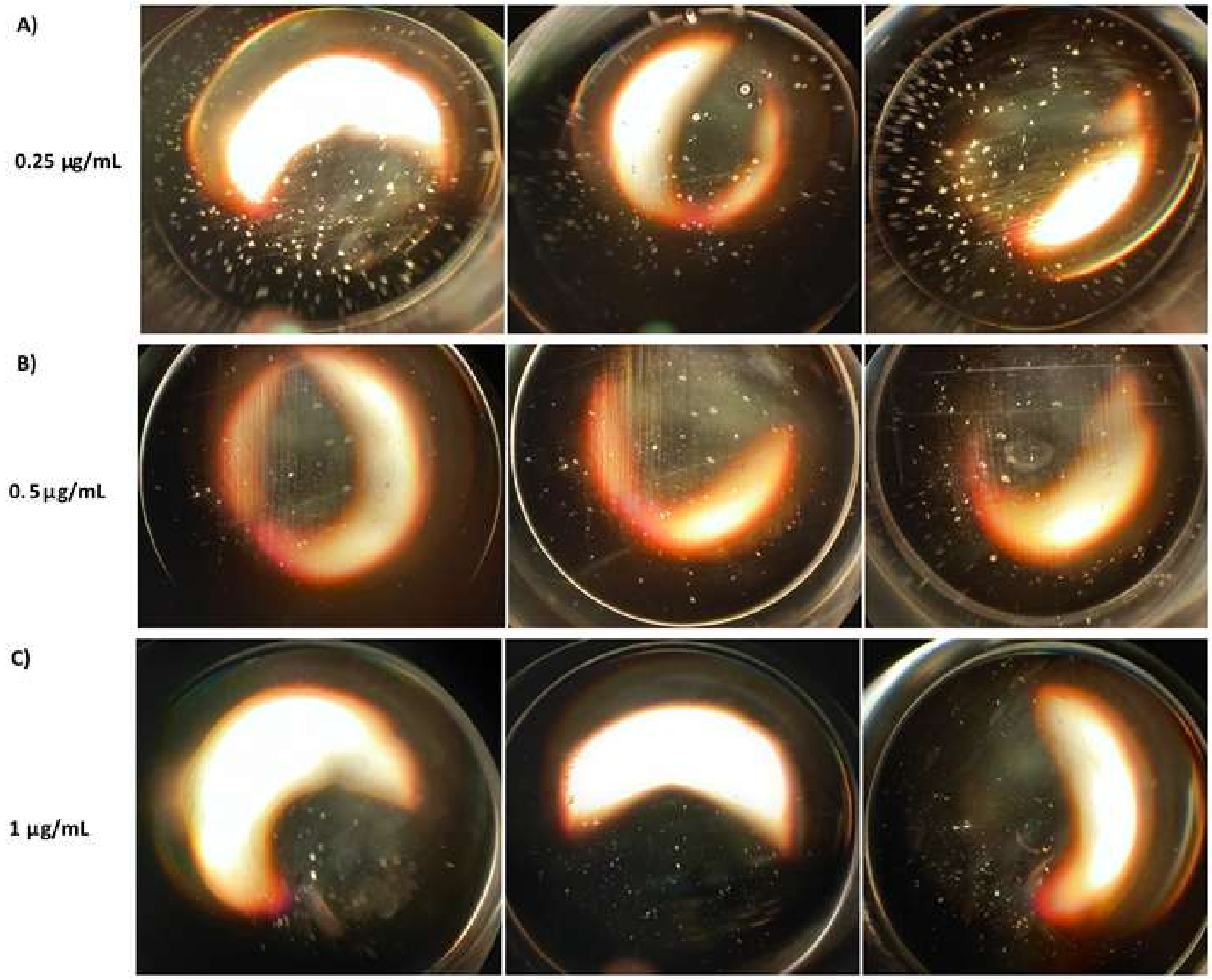

Fig R3 Microscopic examination of the microtiter plate wells after 48h incubation at 35°C (brightfield microscopy, 40× magnification). A: *Candida albican* SC5314 cell growth at 0.25 μg/mL micafungin concentration; B: *Candida albican* SC5314 cell growth at 0.5 μg/mL micafungin concentration; C: *Candida albicans.*SC5314 cell growth at 1 μg/mL micafungin concentration.

Re: Spectrum01310-26R2 (Exploiting Neosartorya (Aspergillus) fischeri antifungal protein 2-defined fungal targets for drug repurposing against Candida albicans)

Dear Dr. László Galgóczy:

Your manuscript has been accepted, and I am forwarding it to the ASM production staff for publication. Your paper will first be checked to make sure all elements meet the technical requirements. ASM staff will contact you if anything needs to be revised before copyediting and production can begin. Otherwise, you will be notified when your proofs are ready to be viewed.

Data Availability: ASM policy requires that data be available to the public upon online posting of the article, so please verify all links to sequence records, if present, and make sure that each number retrieves the full record of the data. If a new accession number is not linked or a link is broken, provide production staff with the correct URL for the record. If the accession numbers for new data are not publicly accessible before the expected online posting of the article, publication may be delayed; please contact ASM production staff immediately with the expected release date.

Publication Fees: For information on publication fees and which article types have charges, please visit our website. We have partnered with Copyright Clearance Center (CCC) to collect author charges. If fees apply to your paper, you will receive a message from no-reply@copyright.com with further instructions. For questions related to paying charges through RightsLink, please contact CCC at ASM_Support@copyright.com or toll free at +1-877-622-5543. CCC makes every attempt to respond to all emails within 24 hours.

ASM Membership: Corresponding authors may join or renew ASM membership to obtain discounts on publication fees. Need to upgrade your membership level? Please contact Customer Service at Service@asmusa.org.

Embargo Policy: A press release may be issued as soon as the manuscript is posted on the Spectrum Latest Articles webpage. The corresponding author will receive an email with the subject line "ASM Journals Author Services Notification" when the article is available online.

Cover Image Submissions: If you would like to submit a potential Cover Image, please email a file and a short legend to spectrum@asmusa.org. Please note that we can only consider images that (i) the authors created or own and (ii) have not been previously published. By submitting, you agree that the image can be used under the same terms as the published article. Image File requirements: TIF/EPS, 7.5 inches wide by 8.25 inches tall (at least 2,250 pixels wide by 2,475 pixels tall), minimum 300 dpi resolution (600 dpi preferred), RGB, and no figure elements, e.g., arrows or panel labels. The legend should be a short description of the image, 1-2 sentences recommended. Please use the ASM Journals Cover Image Sizing Tool (https://journals.asm.org/pb-assets/html/asm-cover-sizing-tool.html) to ensure that your proposed cover image meets our size requirements.

The ASM Journals program strives for constant improvement in our submission and publication process. Please tell us how we can improve your experience by taking this quick Author Survey.

Thank you for submitting your paper to Spectrum.

Sincerely,
Gregory Wiedman
Editor
Microbiology Spectrum

Reviewer #1 (Comments for the Author):

I don't have any concerns.

Reviewer #2 (Comments for the Author):

All concerns have been addressed.
