# Supplementary material for: Exploiting Neosartorya (Aspergillus) fischeri antifungal protein 2-defined fungal targets for drug repurposing against Candida albicans
Source: Microbiol Spectr. 2026 Oct 9;14(11):e01310-26. doi: 10.1128/spectrum.01310-26 (PMC13729217; doi:10.1128/spectrum.01310-26)
Supplement: Supplementary file 2 [file spectrum.01310-26-s0001.pdf]

**Supplemental Material**

# Exploiting *Neosartorya* (*Aspergillus*) *fischeri* antifungal protein 2-defined fungal targets for drug repurposing against *Candida albicans*

Kinga Dán,[a,b,c] Rebeka Papp,[a,c] LiliánaTóth,[a] Gábor Bende,[a,c,d] Lizett Miszlai,[a] László Galgóczy[a]

[a] Department of Biotechnology and Microbiology, Faculty of Science and Informatics, University of Szeged, Szeged, Hungary

[b] Center for Reproductive Medicine, Department of Obstetrics and Gynecology, Albert Szent-Györgyi Health Centre, University of Szeged, Szeged, Hungary

[c] Doctoral School of Biology, Faculty of Science and Informatics, University of Szeged, Szeged, Hungary

[d] Department of Theoretical Health Sciences and Health Management, Faculty of Health Sciences and Social Studies, University of Szeged, Szeged, Hungary

Address correspondence to László Galgóczy, galgoczi@bio.u-szeged.hu

**Table S1** Validation of the CLSI micafungin susceptibility of two independent *Candida albicans* SC5314 stocks determined under standard CLSI M27-A4 conditions (RPMI-1640 + MOPS, 35°C, 24 h).

**A**

24h $OD_{620}$

| µg/mL | | 8 | 4 | 2 | 1 | 0.5 | 0.25 | 0.125 | 0.063 | 0.031 | 0.016 | 0.008 | GC |
|---|---|---|---|---|---|---|---|---|---|---|---|---|---|
| BR 1 | TR1 | 0 | 0 | 0.001 | 0 | -0.005 | -0.001 | 0.018 | 0.294 | 0.276 | 0.272 | 0.296 | 0.301 |
| | TR2 | 0 | -0.001 | -0.002 | -0.002 | -0.002 | -0.001 | 0.015 | 0.26 | 0.247 | 0.257 | 0.238 | 0.236 |
| | TR3 | -0.001 | -0.001 | -0.002 | 0 | -0.002 | -0.003 | 0.022 | 0.289 | 0.309 | 0.324 | 0.314 | 0.281 |
| BR 2 | TR1 | -0.001 | 0.007 | -0.002 | -0.004 | 0.005 | 0 | 0.013 | 0.259 | 0.241 | 0.24 | 0.254 | 0.206 |
| | TR2 | 0 | 0.001 | 0 | -0.002 | 0.008 | -0.001 | 0.011 | 0.276 | 0.265 | 0.278 | 0.292 | 0.257 |
| | TR3 | -0.003 | -0.002 | 0 | 0 | 0 | -0.001 | 0.012 | 0.258 | 0.31 | 0.314 | 0.316 | 0.298 |

Growth rate %

| µg/mL | | 8 | 4 | 2 | 1 | 0.5 | 0.25 | 0.125 | 0.063 | 0.031 | 0.016 | 0.008 | GC |
|---|---|---|---|---|---|---|---|---|---|---|---|---|---|
| BR 1 | TR1 | 0% | 0% | 0% | 0% | -2% | 0% | 6% | 98% | 92% | 90% | 98% | 100% |
| | TR2 | 0% | 0% | -1% | -1% | -1% | 0% | 6% | 110% | 105% | 109% | 101% | 100% |
| | TR3 | 0% | 0% | -1% | 0% | -1% | -1% | 8% | 103% | 110% | 115% | 112% | 100% |
| BR 2 | TR1 | 0% | 3% | -1% | -2% | 2% | 0% | 6% | 126% | 117% | 117% | 123% | 100% |
| | TR2 | 0% | 0% | 0% | -1% | 3% | 0% | 4% | 107% | 103% | 108% | 114% | 100% |
| | TR3 | -1% | -1% | 0% | 0% | 0% | 0% | 4% | 87% | 104% | 105% | 106% | 100% |
| | Mean | 0% | 0% | 0% | -1% | 0% | 0% | 6% | 105% | 105% | 107% | 109% | 100% |
| | Standard deviation | 0% | 1% | 0% | 1% | 2% | 0% | 1% | 12% | 8% | 9% | 8% | 0% |

**B**

24h $OD_{620}$

| µg/mL | | 8 | 4 | 2 | 1 | 0.5 | 0.25 | 0.125 | 0.063 | 0.031 | 0.016 | 0.008 | GC |
|---|---|---|---|---|---|---|---|---|---|---|---|---|---|
| BR 1 | TR1 | 0.002 | 0.002 | -0.001 | 0 | 0 | -0.002 | 0.016 | 0.236 | 0.23 | 0.237 | 0.254 | 0.23 |
| | TR2 | -0.001 | -0.001 | -0.002 | 0.001 | -0.001 | -0.002 | 0.007 | 0.265 | 0.227 | 0.28 | 0.255 | 0.219 |
| | TR3 | -0.001 | -0.001 | -0.002 | 0.002 | 0.001 | 0.003 | 0.013 | 0.289 | 0.271 | 0.333 | 0.317 | 0.291 |
| BR 2 | TR1 | -0.002 | -0.003 | -0.002 | 0.001 | 0.004 | 0.01 | 0.006 | 0.168 | 0.193 | 0.21 | 0.253 | 0.238 |
| | TR2 | -0.002 | 0.001 | 0.001 | 0 | -0.001 | 0.001 | 0.005 | 0.208 | 0.226 | 0.21 | 0.204 | 0.234 |
| | TR3 | -0.001 | 0 | 0.001 | 0 | 0.001 | -0.001 | 0.007 | 0.239 | 0.258 | 0.238 | 0.24 | 0.269 |

Growth rate %

| µg/mL | | 8 | 4 | 2 | 1 | 0.5 | 0.25 | 0.125 | 0.063 | 0.031 | 0.016 | 0.008 | GC |
|---|---|---|---|---|---|---|---|---|---|---|---|---|---|
| BR 1 | TR1 | 1% | 1% | 0% | 0% | 0% | -1% | 7% | 103% | 100% | 103% | 110% | 100% |
| | TR2 | 0% | 0% | -1% | 0% | 0% | -1% | 3% | 121% | 104% | 128% | 116% | 100% |
| | TR3 | 0% | 0% | -1% | 1% | 0% | 1% | 4% | 99% | 93% | 114% | 109% | 100% |
| BR 2 | TR1 | -1% | -1% | -1% | 0% | 2% | 4% | 3% | 71% | 81% | 88% | 106% | 100% |
| | TR2 | -1% | 0% | 0% | 0% | 0% | 0% | 2% | 89% | 97% | 90% | 87% | 100% |
| | TR3 | 0% | 0% | 0% | 0% | 0% | 0% | 3% | 89% | 96% | 88% | 89% | 100% |
| | Mean | 0% | 0% | 0% | 0% | 0% | 1% | 4% | 95% | 95% | 102% | 103% | 100% |
| | Standard deviation | 1% | 1% | 1% | 0% | 1% | 2% | 2% | 15% | 7% | 15% | 11% | 0% |

This validation experiment was performed to confirm that the elevated micafungin concentrations observed under the modified assay conditions used in this study were not attributable to strain-specific differences. Panel A shows the *C. albicans* SC5314 stock maintained in our laboratory, whereas panel B shows an independent SC5314 stock obtained from the Host–Pathogen Interaction Centre (University of Szeged). Under standard CLSI M27-A4 conditions (35°C, 24 h, ≥50% growth inhibition endpoint), both independent SC5314 stocks exhibited identical micafungin MIC values of 0.125 µg/mL, confirming the expected CLSI micafungin-susceptible phenotype of the strain. Abbreviations: BR, biological replicate; GC, growth control; $OD_{620}$, optical density at 620 nm; TR, technical replicate.

**Table S2** Inhibitory activity and interaction analysis of dihydroergocristine mesylate (DHEC) and fluconazole (FLC), flucytosine (5-FC), and micafungin (MCF) combinations against *Candida albicans* SC5314.

| **DHEC→/FLC↓ (μg/mL)** | **128** | **64** | **32** | **16** | **8** | **4** | **2** | **1** | **0.5** | **0** |
|---|---|---|---|---|---|---|---|---|---|---|
| **0.5** | 37±29.4%<br>**0.50±0.34** | 44±26.4%<br>**0.61±0.28** | 45±19.7%<br>**0.63±0.16** | 52±15.4%<br>**0.75±0.10** | 63±13.7%<br>**0.93±0.12** | 65±15.7%<br>**0.94±0.09** | 66±14.3%<br>**0.97±0.03** | 67±11.9%<br>**0.99±0.10** | 62±10.8%<br>**0.92±0.12** | 68±14.8% |
| **0.25** | 23±19%<br>**0.34±0.26** | 17±13.8%<br>**0.25±0.18** | 24±20%<br>**0.33±0.25** | 42±23.1%<br>**0.64±0.20** | 50±17.8%<br>**0.80±0.08** | 54±19.1%<br>**0.85±0.06** | 59±18.3%<br>**0.95±0.05** | 57±15.1%<br>**0.92±0.04** | 59±12.9%<br>**0.96±0.10** | 62±18.6% |
| **0.125** | 14±14%<br>**0.26±0.22** | 20±17.4%<br>**0.36±0.25** | 14±8.4%<br>**0.31±0.14** | 30±16.8%<br>**0.68±0.09** | 37±27%<br>**0.75±0.20** | 42±31.8%<br>**0.84±0.26** | 40±29.2%<br>**0.81±0.24** | 38±27.5%<br>**0.78±0.25** | 39±26.8%<br>**0.81±0.12** | 44±26.8% |
| **0.0625** | 4±5.8%<br>**0.40±0.62** | 9±9.7%<br>**0.63±1.15** | 6±7%<br>**0.12±0.14** | 3±2.8%<br>**0.23±0.34** | 5±4.7%<br>**0.20±0.30** | 4±3.9%<br>**0.29±0.41** | 4±4.7%<br>**0.09±0.19** | 4±6.1%<br>**0.40±0.67** | 5±6.7%<br>**0.05±0.11** | 7±9.5% |
| **0.03125** | 2±2.6%<br>**0.10±0.20** | 8±11%<br>**0.78±1.56** | 2±1.9%<br>**0.29±0.42** | 4±7.6<br>**1.39±2.72** | 4±3.3%<br>**0.76±1.34** | 3±4.5%<br>**0.18±0.35** | 1±1.2%<br>**0.05±0.09** | 3±4.9%<br>**0.05±0.10** | 2±4.3%<br>**0.16±0.33** | 4±6.3% |
| **0.015** | 3±5.2%<br>**0.68±1** | 2±4%<br>**0.42±0.80** | 1±1%<br>**0.06±0.11** | 6±5.6%<br>**1.49±2.08** | 5±5.1<br>**1.29±2.02** | 3±6%<br>**0.32±0.67** | 2±4.1%<br>**0.23±0.46** | 2±4.5%<br>**0.43±0.87** | 2±3%<br>**0.24±0.33** | 4±3.4% |
| **0.0075** | 3±4.3%<br>**0.0±0** | 0±0%<br>**0.0±0** | 2±2.8%<br>**0.0±0** | 6±7.1%<br>**0.0±0** | 8±6.6%<br>**0.0±0** | 6±7.2<br>**0.0±0** | 2±3.5<br>**0.0±0** | 2±3.4<br>**0.0±0** | 4±3.4<br>**0.0±0** | 6±7% |
| **0** | 0±0% | 0±0% | 0±0% | 0±0% | 0±0% | 0±0% | 0±0% | 0±0% | 0±0% | 0±0% |

| **DHEC→/5-FC↓ (μg/mL)** | **128** | **64** | **32** | **16** | **8** | **4** | **2** | **1** | **0.5** | **0** |
|---|---|---|---|---|---|---|---|---|---|---|
| **0.125** | 100±0.57%<br>**1.01±0.03** | 91±9.93%<br>**0.93±0.12** | 94±6.59%<br>**0.96±0.08** | 91±9.98%<br>**0.92±0.12** | 94±7.29%<br>**0.95±0.09** | 81±13.69%<br>**0.82±0.13** | 87±14.57%<br>**0.88±0.13** | 88±11.02%<br>**0.89±0.09** | 91±9.79%<br>**0.92±0.08** | 98±2.47% |
| **0.0625** | 13±9.07%<br>**0.38±0.75** | 23±8.30%<br>**1.06±1.22** | 6±4.13%<br>**0.34±0.40** | 9±6.59%<br>**0.53±0.62** | 12±8.56%<br>**0.64±0.75** | 7±4.82%<br>**0.35±0.40** | 7±5.77%<br>**0.32±0.38** | 9±5.83%<br>**0.43±0.50** | 6±2.39%<br>**0.26±0.30** | 9±6.19 |
| **0.0315** | 1±0.96%<br>**0.13±0.25** | 11±2.42%<br>**2.92±3.38** | 4±4.13%<br>**1.35±1.67** | 5±4.35%<br>**1.75±2.18** | 6±4.64%<br>**1.94±2.35** | 4±5.16<br>**1.96±2.29** | 3±4.57%<br>**1.52±1.95** | 2±2.64%<br>**1.11±0.71** | 1±0.95%<br>**0.46±0.53** | 1+1.27% |

| | | | | | | | | | | |
|---|---|---|---|---|---|---|---|---|---|---|
| **0.015** | 2±2.08%<br>**0.00±0** | 1±1.50%<br>**0.00±0** | 4±4.07%<br>**0.00±0** | 5±5.48%<br>**0.00±0** | 6±4.53%<br>**0.00±0** | 2±3.30%<br>**0.00±0** | 1±1.50%<br>**0.00±0** | 1±1.00%<br>**0.00±0** | 1±1.00%<br>**0.00±0** | 0±0% |
| **0.0075** | 0±0.08%<br>**0.00±0** | 2±1.93%<br>**0.00±0** | 2±3.16%<br>**0.00±0** | 4±6.65%<br>**0.00±0** | 4±4.58%<br>**0.00±0** | 0±0%<br>**0.00±0** | 0±0%<br>**0.00±0** | 0±0%<br>**0.00±0** | 0±0%<br>**0.00±0** | 0±0% |
| **0.00375** | 0±0%<br>**0.00±0** | 0±0%<br>**0.00±0** | 0±0%<br>**0.00±0** | 0±0%<br>**0.00±0** | 0±0%<br>**0.00±0** | 0±0.10%<br>**0.00±0** | 0±0%<br>**0.00±0** | 0±0%<br>**0.00±0** | 0±0.20%<br>**0.00±0** | 0±0% |
| **0.00187** | 0±0%<br>**0.00±0** | 0±0%<br>**0.00±0** | 0±0%<br>**0.00±0** | 0±0%<br>**0.00±0** | 0±0%<br>**0.00±0** | 0±0%<br>**0.00±0** | 0±0%<br>**0.00±0** | 0±0%<br>**0.00±0** | 0±0%<br>**0.00±0** | 0±0.17% |
| **0** | 0±0% | 0±0% | 0±0% | 0±0% | 0±0% | 0±0% | 0±0% | 0±0% | 0±0% | 0±0% |

| **DHEC→/MCF↓ (μg/mL)** | **128** | **64** | **32** | **16** | **8** | **4** | **2** | **1** | **0.5** | **0** |
|---|---|---|---|---|---|---|---|---|---|---|
| **1** | 99±1.38%<br>**1.00±0.01** | 100±0.33%<br>**1.01±0.02** | 99±0.86%<br>**1.00±0.01** | 100±0.73%<br>**1.01±0.01** | 100±0.67%<br>**1.00±0.01** | 100±0.42%<br>**1.00±0.02** | 100±0.54%<br>**1.01±0.02** | 99±2.49%<br>**0.99±0.01** | 99±0.48%<br>**1.00±0.01** | 99±1.79% |
| **0.5** | 100±0.17%<br>**1.02±0.03** | 99±0.47%<br>**1.01±0.03** | 100±0.43%<br>**1.01±0.03** | 99±0.47%<br>**1.01±0.04** | 99±1.09%<br>**1.01±0.02** | 99±0.49%<br>**1.01±0.03** | 100±0.97%<br>**1.01±0.02** | 99±1.54%<br>**1.01±0.02** | 98±2.42%<br>**1.00±0.01** | 98±3.13% |
| **0.25** | 99±2.55%<br>**5.24±4.97** | 98±1.24%<br>**5.30±4.88** | 99±0.54%<br>**5.11±4.93** | 92±14.06%<br>**5.10±5.05** | 80±38.72%<br>**4.61±5.36** | 40±40.45%<br>**1.63±1.86** | 33±43.47%<br>**1.07±1.07** | 38±40.42%<br>**1.46±1.44** | 33±38.69%<br>**0.90±0.46** | 36±32.78% |
| **0.125** | 7±5.55%<br>**0.52±0.70** | 26±48.70%<br>**0.38±0.55** | 32±45.03%<br>**0.35±0.35** | 24±43.68%<br>**0.42±0.76** | 17±16.95%<br>**1.02±1.37** | 16±24.76%<br>**0.18±0.32** | 17±31.86%<br>**1.11±2.19** | 6±7.56%<br>**0.59±0.69** | 4±6.12<br>**0.30±0.45** | 21±33.60% |
| **0.0625** | 0±0%<br>**0.00±0** | 4±8.65%<br>**1.53±3.06** | 6±10.69%<br>**2.35±4.57** | 4±6.95%<br>**1.16±2.12** | 2±2.65%<br>**2.94±5.71** | 3±5.08%<br>**0.49±0.70** | 0±0%<br>**0.00±0** | 2±4.25%<br>**0.14±0.29** | 3±6.24%<br>**0.35±00.69** | 3±3.31% |
| **0.03125** | 0±0%<br>**0.00±0** | 1±2.17%<br>**0.10±0.20** | 2±3.73%<br>**0.16±0.32** | 0±0%<br>**0.00±0** | 8±14.50%<br>**0.41±0.64** | 5±6.71%<br>**0.60±0.70** | 4±5.34%<br>**1.42±2.60** | 1±2.08%<br>**0.06±0.12** | 3±5.98%<br>**0.24±0.48** | 4±5.04% |
| **0.0015** | 0±0%<br>**0.00±0** | 0±0%<br>**0.00±0** | 2±2.53%<br>**0.24±0.28** | 1±2.77%<br>**0.18±0.37** | 1±2.34%<br>**0.07±0.13** | 2±2.65%<br>**0.41±0.49** | 4±7.71%<br>**0.46±0.93** | 3±6.32<br>**0.23±0.47** | 6±9.07%<br>**0.79±1.21** | 2±2.89% |
| **0** | 0±0% | 0±0% | 2±2.59% | 1±1.04% | 4±6.12% | 0±0% | 1±1.38% | 2±4.16% | 2±2.95% | 0±0% |

DHEC concentrations are displayed in the columns and FLC, 5-FC, MCF concentrations in the rows. Inhibitory activity (IP, %) was determined following combinatorial treatment with DHEC and FLC, 5-FC, MCF and represents percentage growth inhibition rather than residual growth. Interaction ratios (IR) were calculated according to the Abbott formula and are indicated below the corresponding IP values. Green cells indicate synergistic interactions (IR > 1.5), orange cells indicate additive effects (IR = 0.5–1.5), and gray cells indicate no interaction. Synergistic interactions indicate greater-than-expected inhibitory effects relative to the individual treatments and do not necessarily correspond to complete growth inhibition. Cells outlined in black represent approx. 100% growth inhibition.

**Table S3** Inhibitory activity and interaction analysis of meclizine dihydrochloride (MECD and amphotericin B (AMB). fluconazole (FLC), flucytosine (5-FC), and micafungin (MCF) combinations against *Candida albicans* SC5314.

| **MECD→/AMB↓ (μg/mL)** | **256** | **128** | **64** | **32** | **16** | **8** | **4** | **2** | **1** | **0** |
|---|---|---|---|---|---|---|---|---|---|---|
| **0.5** | 100±0,19%<br>**1,24±0,26** | 100±0%<br>**1,20±0,22** | 100±0%<br>**1,23±0,25** | 99±1,07%<br>**1,23±0,25** | 100±0,19%<br>**1,24±0,26** | 100±0,19%<br>**1,24±0,26** | 100±0,11%<br>**1,24±0,26** | 92±9,91%<br>**1,18±0,33** | 82±19,78%<br>**0,98±0,05** | 84±17,62% |
| **0.25** | 73±31,39%<br>**1,54±0,59** | 75±29,26%<br>**1,45±0,75** | 76±27,60%<br>**1,56±0,59** | 75±28,92%<br>**1,63±0,71** | 74±30,63%<br>**1,57±0,65** | 70±27,91%<br>**1,53±0,73** | 74±30,46%<br>**1,61±0,77** | 71±33%<br>**1,46±0,54** | 66±39,35%<br>**1,25±0,41** | 59±41,21% |
| **0.125** | 72±32,52%<br>**9,75±11,34** | 73±31,29%<br>**9,11±14,52** | 73±31,61%<br>**4,97±3,32** | 69±35,43%<br>**8,22±8,61** | 66±39,87%<br>**6,94±6,78** | 66±39,44%<br>**7,21±7,38** | 67±38,76%<br>**7,65±8,31** | 64±42,22%<br>**5,39±3,46** | 52±52,76%<br>**1,82±1,22** | 22±21,54% |
| **0.0625** | 36±28,16%<br>**0,72±0,85** | 44±24,49%<br>**0,99±0,66** | 48±24,19%<br>**1,61±1,35** | 41±30,93%<br>**0,78±0,90** | 48±39,31%<br>**0,99±1,17** | 44±32,06%<br>**0,86±1,04** | 44±31,54%<br>**0,86±1,02** | 23±26,85%<br>**0,57±0,66** | 20±22,94%<br>**0,49±0,57** | 20±23,44% |
| **0.03125** | 26±16,74%<br>**1,44±1,67** | 30±24,85%<br>**1,25±1,18** | 30±23,34%<br>**1,58±1,61** | 26±25,21%<br>**1,35±1,56** | 27±19,58%<br>**1,60±1,85** | 19±19,29%<br>**1,31±1,51** | 21±21,31%<br>**1,47±1,71** | 15±17,73%<br>**1,15±1,34** | 17±19,32%<br>**1,25±1,45** | 7±7,94% |
| **0.015** | 10±10,37%<br>**3,43±4,78** | 20±15,15%<br>**1,73±2,07** | 22±17,19%<br>**17,90±22,64** | 17±17,96%<br>**4,79±5,73** | 19±21,93%<br>**22,29±26,64** | 12±13,58%<br>**13,66±15,95** | 17±17,05%<br>**20,61±21,42** | 6±8,30%<br>**5,87±11,75** | 3±5,42%<br>**2,71±5,42** | 0±0,48% |
| **0.0075** | 2±3,29%<br>**0,00±0** | 15±8,89%<br>**0,99±0,96** | 10±7,09%<br>**0,41±0,75** | 4±4,57%<br>**0,13±0,26** | 4±5,51%<br>**0,67±1,34** | 1±1,41%<br>**0,05±0,09** | 1±2,03%<br>**0,24±0,48** | 0±0,09%<br>**0,00±0** | 0±0%<br>**0,00±0** | 1±2,13% |
| **0** | 0±0% | 12±11,16% | 11±5,53% | 6±5,91% | 8±5,97% | 0±0% | 1±2,90% | 2±3,65% | 3±6% | 0±0% |
| **MECDK→/FLC↓ (μg/mL)** | **256** | **128** | **64** | **32** | **16** | **8** | **4** | **2** | **1** | **0** |
| **0.5** | 66±15,10%<br>**0,96±0,19** | 67±14,77%<br>**0,97±0,11** | 70±13,32%<br>**0,97±0,12** | 72±12,75%<br>**1,03±0,12** | 69±13,31%<br>**0,96±0,15** | 65±15,02%<br>**0,94±0,10** | 67±15,84%<br>**0,96±0,04** | 69±17,62%<br>**0,99±0,11** | 67±16,65%<br>**0,94±0,05** | 70±18,32% |
| **0.25** | 10±12,25%<br>**0,12±0,15** | 11±11,8%<br>**0,16±0,14** | 17±13,95%<br>**0,22±0,17** | 18±17,22%<br>**0,23±0,20** | 18±13,62%<br>**0,25±0,16** | 17±20,97%<br>**0,23±0,25** | 35±41,09%<br>**0,42±0,50** | 49±31,55%<br>**0,67±0,28** | 60±20,09%<br>**0,89±0,16** | 65±19,28% |
| **0.125** | 5±10,44%<br>**0,07±0,14** | 3±6,87%<br>**0,05±0,09** | 18±13,53%<br>**0,36±0,35** | 7±8,77%<br>**0,09±0,14** | 9±10,14%<br>**0,18±0,20** | 1±1,55%<br>**0,03±0,03** | 0±0%<br>**0,00±0** | 6±11,1%<br>**0,07±0,14** | 34±37,06%<br>**0,54±0,55** | 38±34,44% |

| | | | | | | | | | | |
|---|---|---|---|---|---|---|---|---|---|---|
| **0.0625** | 1±1,36%<br>**0,00±0** | 6±11,47%<br>**0,47±0,94** | 13±11,45%<br>**0,98±1,03** | 5±4,40%<br>**0,78±0,67** | 4±3,69%<br>**0,23±0,24** | 2±2,72%<br>**0,04±0,09** | 0±0,90%<br>**0,02±0,04** | 1±1,15%<br>**0,02±0,05** | 0±0,49%<br>**0,03±0,07** | 2±3,33% |
| **0.03125** | 1±1,72%<br>**0,10±0,21** | 4±6,08%<br>**0,15±0,31** | 12±8,77%<br>**0,83±0,70** | 6±7,98%<br>**0,64±0,76** | 9±12,14%<br>**0,38±0,53** | 3±6,63%<br>**0,20±0,41** | 2±3,44%<br>**0,09±0,18** | 2±3,36%<br>**0,08±0,16** | 2±3,11%<br>**0,09±0,17** | 4±3,27% |
| **0.015** | 0±0,93%<br>**0,09±0,29** | 2±3,05%<br>**0,02±0,45** | 18±9,79%<br>**1,29±0,65** | 6±8,39%<br>**0,64±0,75** | 10±8,91%<br>**0,48±0,35** | 2±4,12%<br>**0,11±0,22** | 2±4,42%<br>**0,10±0,20** | 2±4,75%<br>**0,10±0,20** | 0±0,48%<br>**0,02±0,04** | 3±3,25% |
| **0.0075** | 0±0%<br>**0,00±0** | 2±4,91%<br>**0,41±0,81** | 13±9,43%<br>**1,00±0,91** | 8±5,41%<br>**1,00±0,58** | 11±11,89%<br>**0,53±0,52** | 3±5,95%<br>**0,26±0,39** | 3±6,79%<br>**0,19±0,37** | 5±4,22%<br>**0,21±0,24** | 3±6,96%<br>**0,21±0,43** | 3±2,84% |
| **0** | 2±3,27% | 1±2,95% | 12±12,30% | 5±5,28% | 15±10,71% | 4±7,28% | 5±9% | 5±9,57% | 3±6,67% | 0±0% |
| **MECD→/5-FC↓ (µg/mL)** | **256** | **128** | **64** | **32** | **16** | **8** | **4** | **2** | **1** | **0** |

| | | | | | | | | | | |
|---|---|---|---|---|---|---|---|---|---|---|
| **0.125** | 99±0,79%<br>**1,00±0,01** | 97±4,29%<br>**0,97±0,04** | 100±0%<br>**1,00±0,01** | 95±6,37%<br>**0,95±0,06** | 98±2,18%<br>**0,99±0,02** | 97±4,40%<br>**0,97±0,05** | 98±1,61%<br>**0,98±0,02** | 96±2,72%<br>**0,97±0,03** | 97±2,38%<br>**0,97±0,02** | 100±0,77% |
| **0.0625** | 12±3,15%<br>**0,55±0,13** | 14±8,00%<br>**0,76±0,48** | 15±2,23%<br>**0,77±0,05** | 13±2,67%<br>**0,63±0,14** | 16±5,42%<br>**0,82±0,23** | 12±3,94%<br>**0,62±0,17** | 11±3,76%<br>**0,53±0,14** | 9±0,89%<br>**0,40±0,06** | 12±5,47%<br>**0,64±0,27** | 19±1,86% |
| **0.0315** | 4±5,56%<br>**0,43±0,52** | 15±12,36%<br>**1,60±1,22** | 4±2,91%<br>**0,46±0,33** | 8±4,76%<br>**1,00±0,71** | 16±7,42%<br>**2,00±1,26** | 9±7,23%<br>**1,14±0,95** | 9±2,78%<br>**0,99±0,51** | 8±3,41%<br>**0,77±0,51** | 4±2,24%<br>**0,46±0,30** | 9±2,44% |
| **0.015** | 4±3,18%<br>**0,78±1,18** | 12±16,42%<br>**0,00±0** | 7±5,85%<br>**2,09±2,64** | 3±5,07%<br>**0,56±1,13** | 3±3,51%<br>**0,82±0,95** | 6±5,97%<br>**1,66±1,92** | 2±2,14%<br>**0,41±0,50** | 0±0,50<br>**0,00±0** | 1±1,41<br>**0,88±1,44** | 2±2,13% |
| **0.0075** | 1±1,08%<br>**0,07±0,14** | 0±0%<br>**0,00±0** | 2±3,74%<br>**0,00±0** | 0±1,00%<br>**0,00±0** | 4±4,82%<br>**0,00±0** | 3±5,07%<br>**0,00±0** | 2±3,42%<br>**0,24±0,49** | 0±0%<br>**0,00±0** | 0±0,17%<br>**0,00±0** | 0±0% |
| **0.00375** | 2±4,02%<br>**0,30±0,50** | 2±3,50%<br>**0,00±0** | 9±9,92%<br>**0,00±0** | 4±7,96%<br>**0,04±0,08** | 10±11,90%<br>**0,00±0** | 5±10,98%<br>**0,00±0** | 2±2,82%<br>**0,19±0,38** | 0±0%<br>**0,00±0** | 1±1,33%<br>**0,67±1,33** | 0±0% |
| **0.00187** | 4±4,55%<br>**0,47±0,58** | 5±9,42%<br>**0,00±0** | 9±10,21%<br>**0,00±0** | 3±2,94%<br>**0,33±0,67** | 7±10,43%<br>**0,00±0** | 2±3,41%<br>**0,00±0** | 3±4,32%<br>**0,33±0,65** | 1±0,87%<br>**0,14±0,22** | 0±0%<br>**0,00±0** | 0±0% |
| **0** | 4±4,35% | 0±0,50% | 0±0% | 1±2,00% | 0±0% | 0±0% | 2±3,50% | 3±3,83% | 0±0,50% | 0±0% |
| **MECD→/MCF↓ (µg/mL)** | **256** | **128** | **64** | **32** | **16** | **8** | **4** | **2** | **1** | **0** |

| | | | | | | | | | | |
|---|---|---|---|---|---|---|---|---|---|---|
| **1** | 100±0%<br>**1,01±0** | 98±2,21%<br>**0,99±0,02** | 100±0,31%<br>**1,00±0** | 100±0%<br>**1,01±0,01** | 100±0%<br>**1,01±0** | 98±2,28%<br>**0,99±0,02** | 97±4,58%<br>**0,98±0,04** | 95±7,47%<br>**0,95±0,07** | 98±2,03%<br>**0,99±0,02** | 99±0,48% |
| **0.5** | 100±1,17%<br>**1,00±0,01** | 99±1,61%<br>**1,00±0,02** | 100±0,54%<br>**1,00±0,01** | 100±0%<br>**1,00±0** | 100±0,09%<br>**1,00±0** | 98±2,17%<br>**0,99±0,02** | 98±3,60%<br>**0,98±0,03** | 97±4,27%<br>**0,97±0,04** | 98±1,65%<br>**0,98±0,02** | 100±0,18% |
| **0.25** | 73±30,0%<br>**0,83±0,40** | 81±35,08%<br>**0,91±0,42** | 83±19,65%<br>**0,93±0,30** | 96±4,86%<br>**1,07±0,15** | 99±1,09%<br>**1,11±0,15** | 97±0,80%<br>**1,09±0,14** | 93±8,37%<br>**1,03±0,04** | 92±6,39%<br>**1,01±0,07** | 92±11,33%<br>**1,02±0,05** | 90±10,91% |
| **0.125** | 7±11,03%<br>**0,94±1,63** | 9±12,16%<br>**0,78±0,94** | 26±42,94%<br>**1,36±2,21** | 4±5,04%<br>**0,20±0,30** | 21±35,19%<br>**1,52±2,60** | 0±0%<br>**0,00±0** | 5±4,97%<br>**0,37±0,67** | 22±40,46%<br>**0,20±0,40** | 18±34,77%<br>**0,59±1,11** | 4±7,88% |
| **0.0625** | 2±3,48%<br>**0,56±1,11** | 3±3,61%<br>**0,20±0,24** | 4±7,83%<br>**0,14±0,28** | 4±4,23%<br>**0,20±0,24** | 8±9,51%<br>**1,40±2,32** | 3±6,05%<br>**0,25±0,50** | 2±2,63%<br>**0,48±0,87** | 0±0%<br>**0,00±0** | 0±0,62%<br>**0,03±0,05** | 4±5,72% |
| **0.03125** | 1±1,25%<br>**0,05±0,10** | 4±7,30%<br>**0,23±0,46** | 5±7,61%<br>**0,26±0,30** | 2±2,06%<br>**0,08±0,09** | 6±7,51%<br>**0,69±0,99** | 2±4,63%<br>**0,19±0,38** | 2±4,72%<br>**0,14±0,25** | 1±1,78%<br>**0,11±0,16** | 2±3,02%<br>**0,12±0,25** | 4±5,79% |
| **0.0015** | 1±2%<br>**0,07±0,15** | 3±6,45%<br>**0,18±0,36** | 6±8,57%<br>**0,29±0,36** | 0±0,35%<br>**0,01±0,01** | 1±2,67%<br>**0,07±0,13** | 1±2,22%<br>**0,14±0,28** | 2±3,56%<br>**0,14±0,28** | 2±1,83%<br>**0,09±0,11** | 1±1,05%<br>**0,04±0,08** | 5±6,57% |
| **0** | 0±0% | 2±2,69% | 6±8,62% | 7±8,15% | 3±6,41% | 0±0% | 1±2,49% | 4±4,33% | 0±0,17% | 0±0% |

MECD concentrations are displayed in the columns and AMB, FLC, 5-FC, MCF concentrations in the rows. Inhibitory activity (IP, %) was determined following combinatorial treatment with DHEC and AMB, FLC, 5-FC, MCF and represents percentage growth inhibition rather than residual growth. Interaction ratios (IR) were calculated according to the Abbott formula and are indicated below the corresponding IP values. Green cells indicate synergistic interactions (IR > 1.5), orange cells indicate additive effects (IR = 0.5–1.5), and gray cells indicate no interaction. Synergistic interactions indicate greater-than-expected inhibitory effects relative to the individual treatments and do not necessarily correspond to complete growth inhibition. Cells outlined in black represent approx. 100% growth inhibition.

**Table S4** Statistical analysis of toxicity data for amphotericin B (AMB), dihydroergocristine mesylate (DHEC), and their combination using GraphPad Prism 8.

| | IPS vs. AMB | IPS vs. DHEC | IPS vs. AMB+DHEC |
|---|---|---|---|
| **Log–rank (Mantel–Cox) test** | | | |
| p–value | 0,7122 | 0,2002 | 0,1594 |
| Are the survival curves sig different? | no | no | no |
| **Gehan–Breslow–Wilcoxon test** | | | |
| p–value | 0,6409 | 0,1812 | 0,1567 |
| Are the survival curves sig different? | no | no | no |
| | **Untreated vs. AMB** | **Untreated vs. DHEC** | **Untreated vs. AMB+DHEC** |
| **Log–rank (Mantel–Cox) test** | | | |
| p–value | 0,0334 | 0,0037 | 0,5299 |
| Are the survival curves sig different? | yes | yes | no |
| **Gehan–Breslow–Wilcoxon test** | | | |
| p–value | 0,0296 | 0,0033 | 0,5299 |
| Are the survival curves sig different? | yes | yes | no |
| | **AMB vs. AMB+DHEC** | **DHEC vs. AMB+DHEC** | **Untreated vs. IPS** |
| **Log–rank (Mantel–Cox) test** | | | |
| p–value | 0,0573 | 0,0038 | 0,0775 |
| Are the survival curves sig different? | no | yes | no |
| **Gehan–Breslow–Wilcoxon test** | | | |
| p–value | 0,0466 | 0,0032 | 0,0749 |
| Are the survival curves sig different? | yes | yes | no |

IPS: insect physiological saline

**Table S5** Statistical analysis of the survival of *Galleria mellonella* larvae infected with *Candida albicans* SC5314 following treatment with amphotericin B (AMB), dihydroergocristine mesylate (DHEC), and their combination, performed using GraphPad Prism 8.

| | **IPS vs. *C. albicans*** | **IPS vs. *C. albicans*+AMB** |
|---|---|---|
| **Log–rank (Mantel–Cox) test** | | |
| p–value | 0,0183 | 0,0247 |
| Are the survival curves sig different? | yes | yes |
| **Gehan–Breslow–Wilcoxon test** | | |
| p–value | 0,0295 | 0,0273 |
| Are the survival curves sig different? | yes | yes |
| | **IPS vs. *C. albicans*+DHEC** | **IPS vs. *C. albicans*+AMB–DHEC** |
| **Log–rank (Mantel–Cox) test** | | |
| p–value | 0,0027 | 0,4311 |
| Are the survival curves sig different? | yes | no |
| **Gehan–Breslow–Wilcoxon test** | | |
| p–value | 0,0040 | 0,5038 |
| Are the survival curves sig different? | yes | no |
| | ***C. albicans* vs. *C. albicans*+AMB** | ***C. albicans* vs. *C. albicans*+DHEC** |
| **Log–rank (Mantel–Cox) test** | | |
| p–value | 0,9975 | 0,4962 |
| Are the survival curves sig different? | no | no |
| **Gehan–Breslow–Wilcoxon test** | | |
| p–value | 0,7482 | 0,3812 |
| Are the survival curves sig different? | no | no |
| | ***C. albicans* vs. *C. albicans*+AMB–DHEC** | ***C. albicans*+AMB vs. *C. albicans*+DHEC** |
| **Log–rank (Mantel–Cox) test** | | |
| p–value | 0,1130 | 0,4918 |
| Are the survival curves sig different? | no | no |
| **Gehan–Breslow–Wilcoxon test** | | |
| p–value | 0,1266 | 0,5837 |
| Are the survival curves sig different? | no | no |
| | ***C. albicans*+AMB vs. *C. albicans*+AMB–DHEC** | ***C. albicans*+DHEC vs. *C. albicans*+AMB–DHEC** |
| **Log–rank (Mantel–Cox) test** | | |
| p–value | 0,1150 | 0,0220 |
| Are the survival curves sig different? | no | yes |
| **Gehan–Breslow–Wilcoxon test** | | |
| p–value | 0,0714 | 0,0158 |
| Are the survival curves sig different? | no | yes |

IPS: insect physiological saline

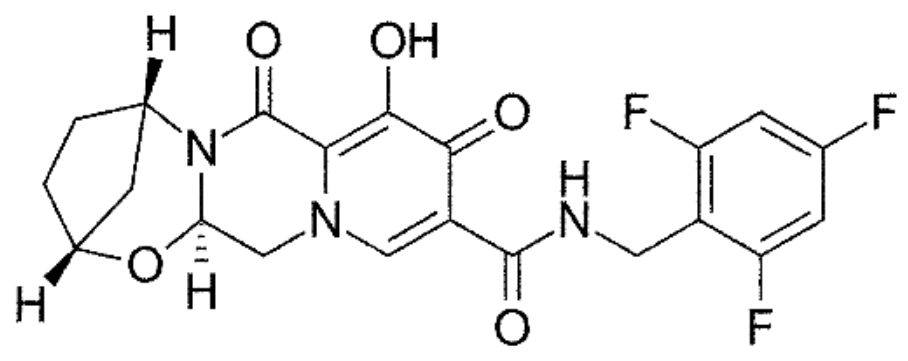

Bictegravir

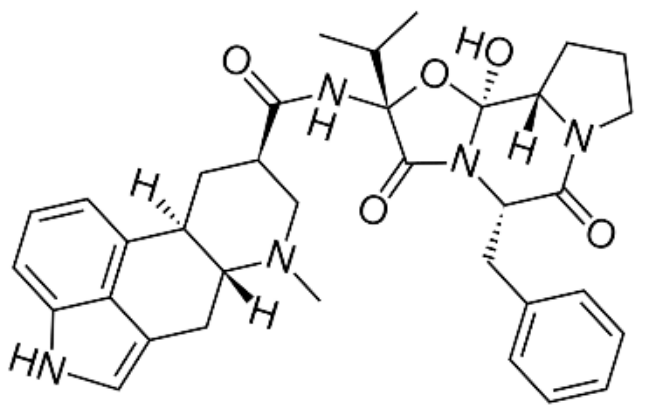

Dihydroergocristine mesylate

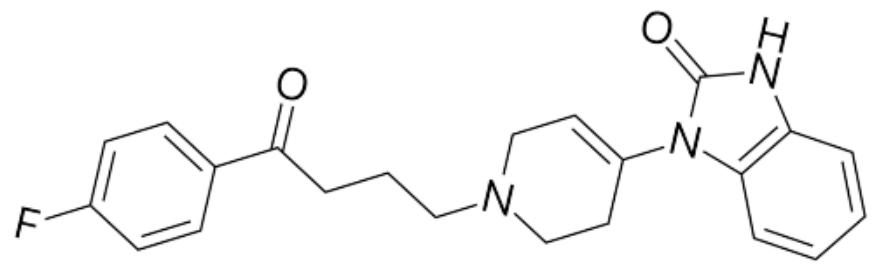

Droperidol

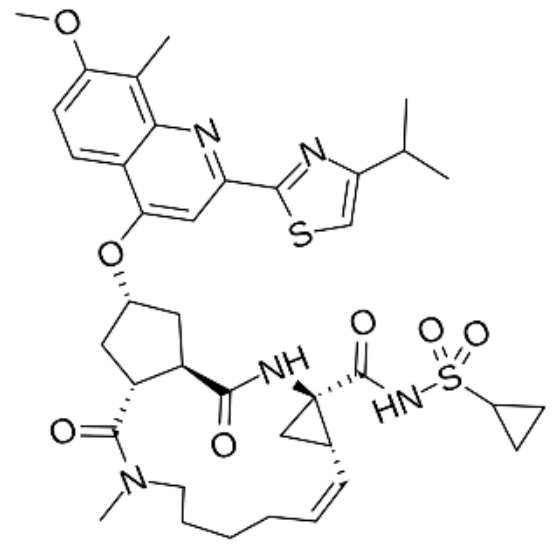

Simeprevir

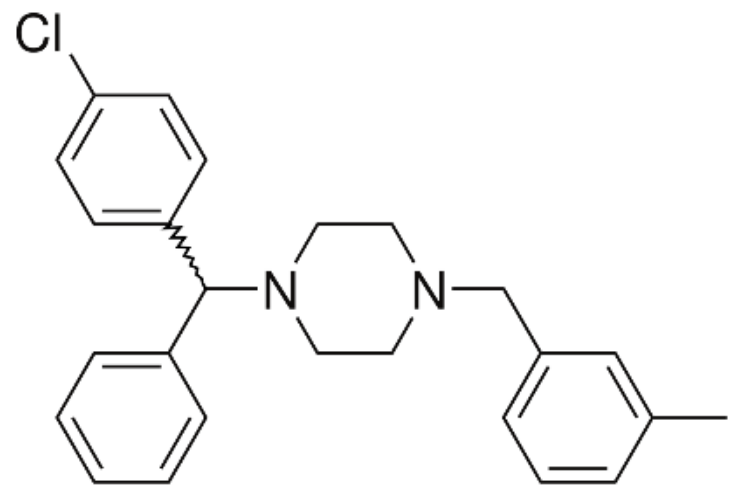

Meclizine dihydrochloride

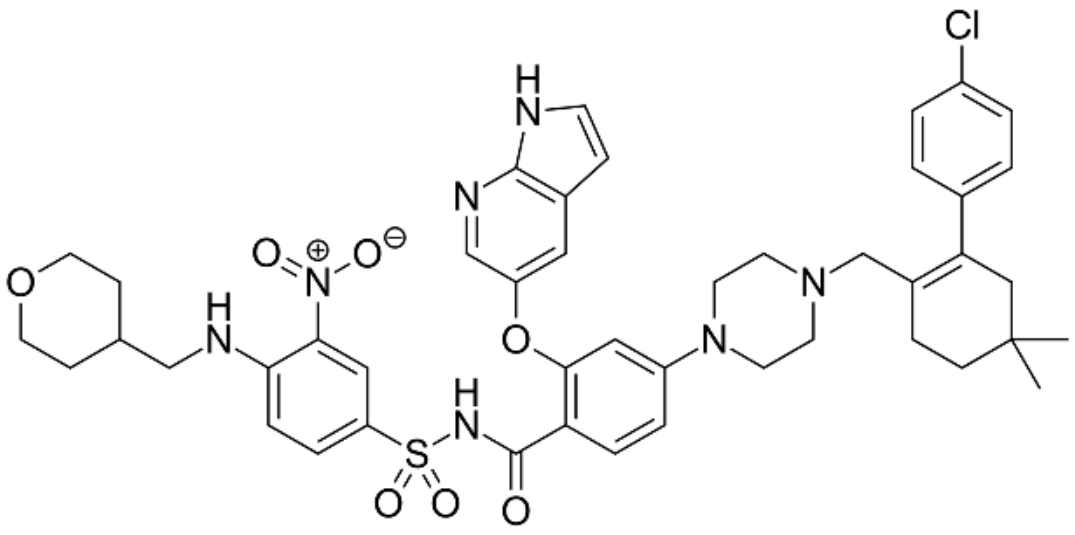

Venetoclax

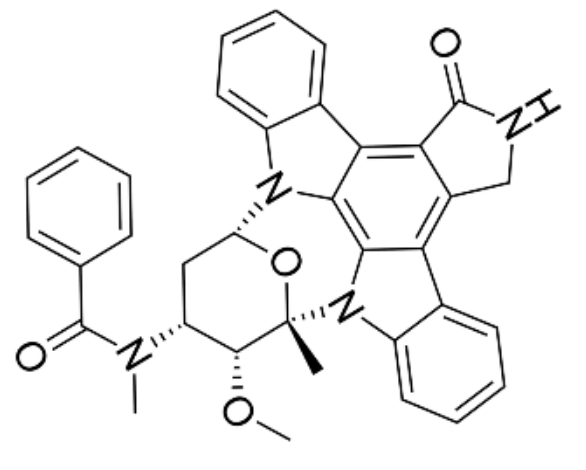

Midostaurin

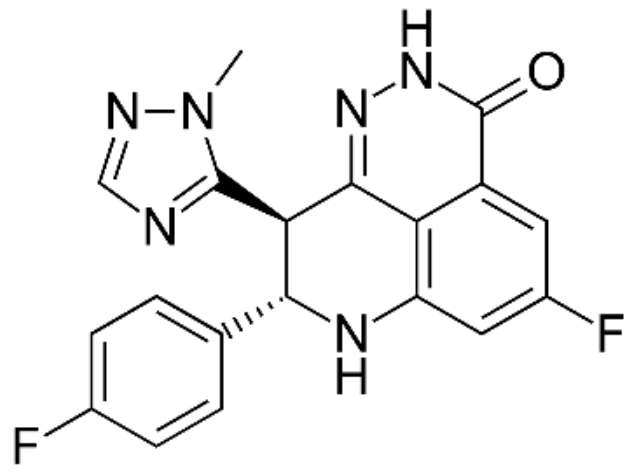

Talazoparib

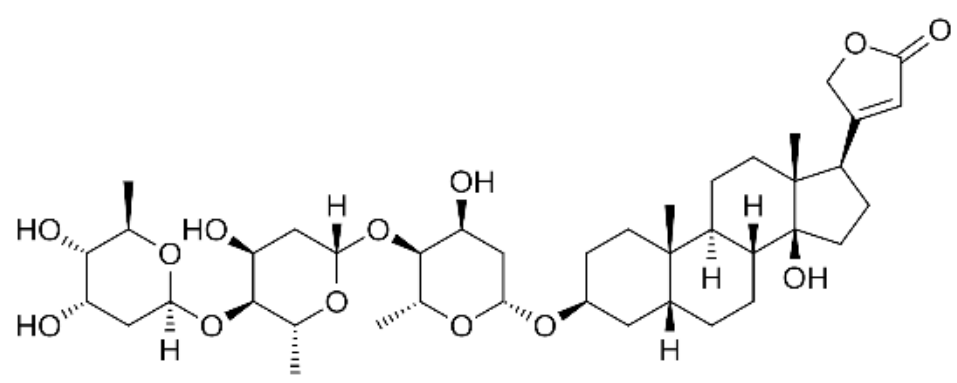

Digitoxin

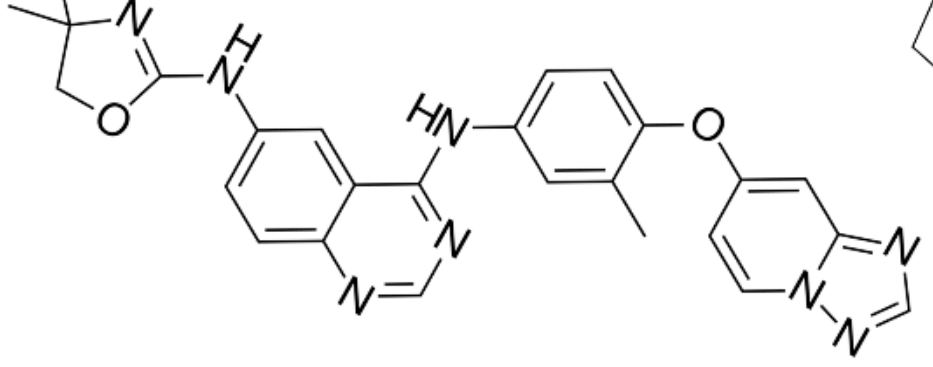

Tucatinib

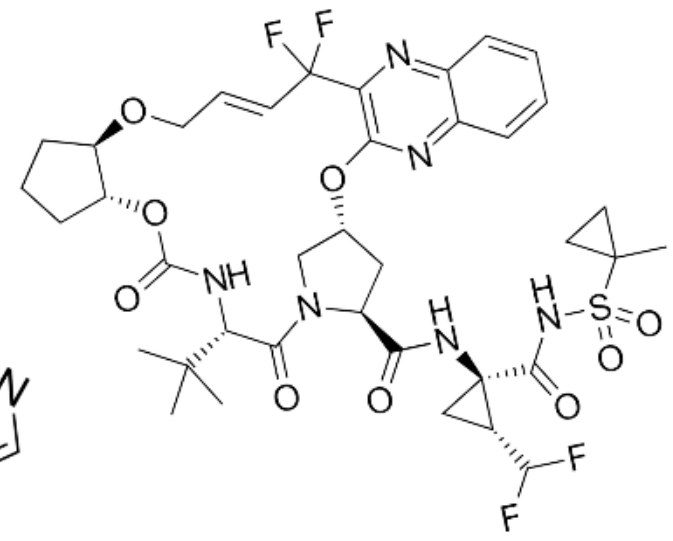

Glecaprevir

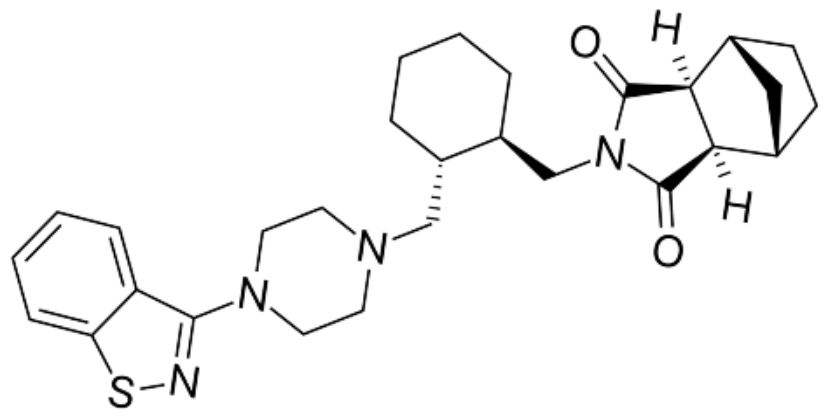

Lurasidone

**Fig S1** Chemical structures of candidate compounds with predicted affinity for the binding pockets of NFAP2.

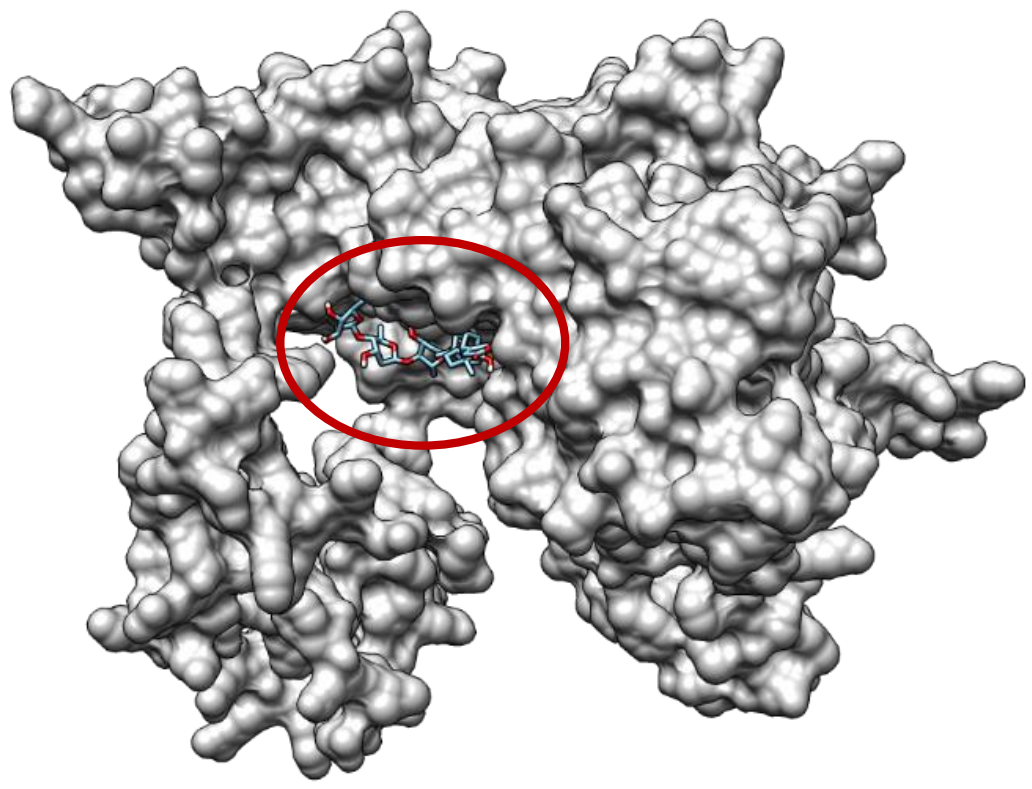

Gad1p – Digitoxin

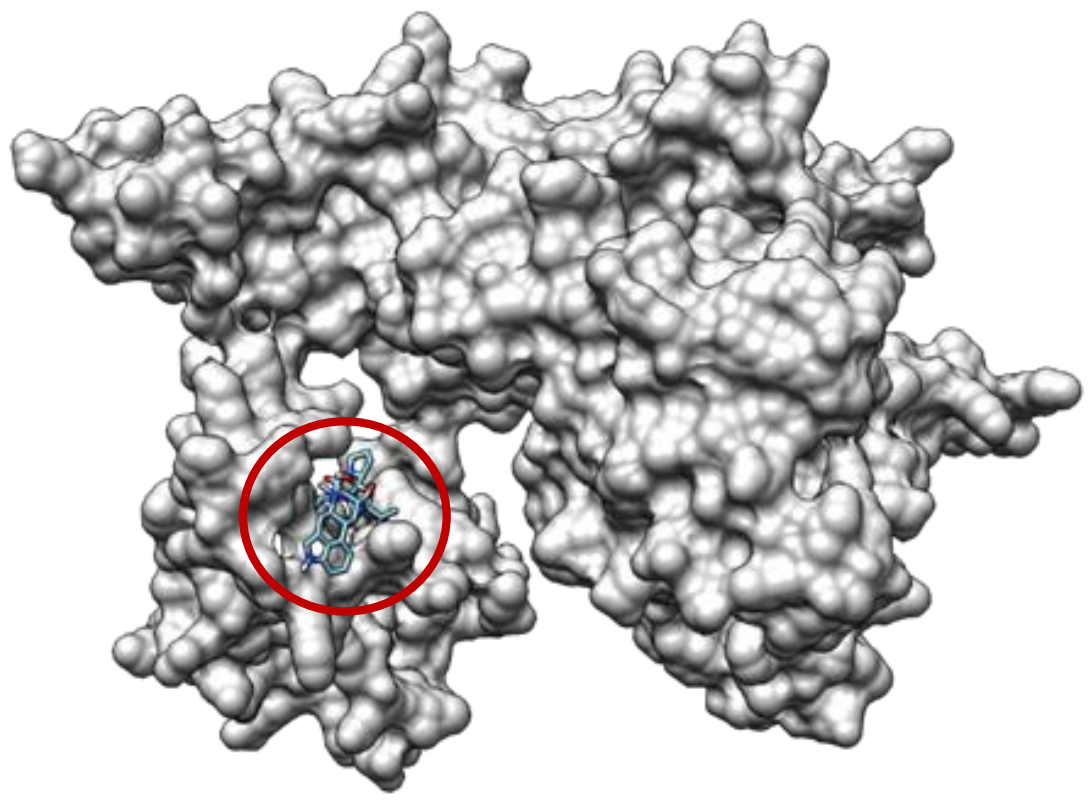

Gad1p – Dihydroergocristine mesylate

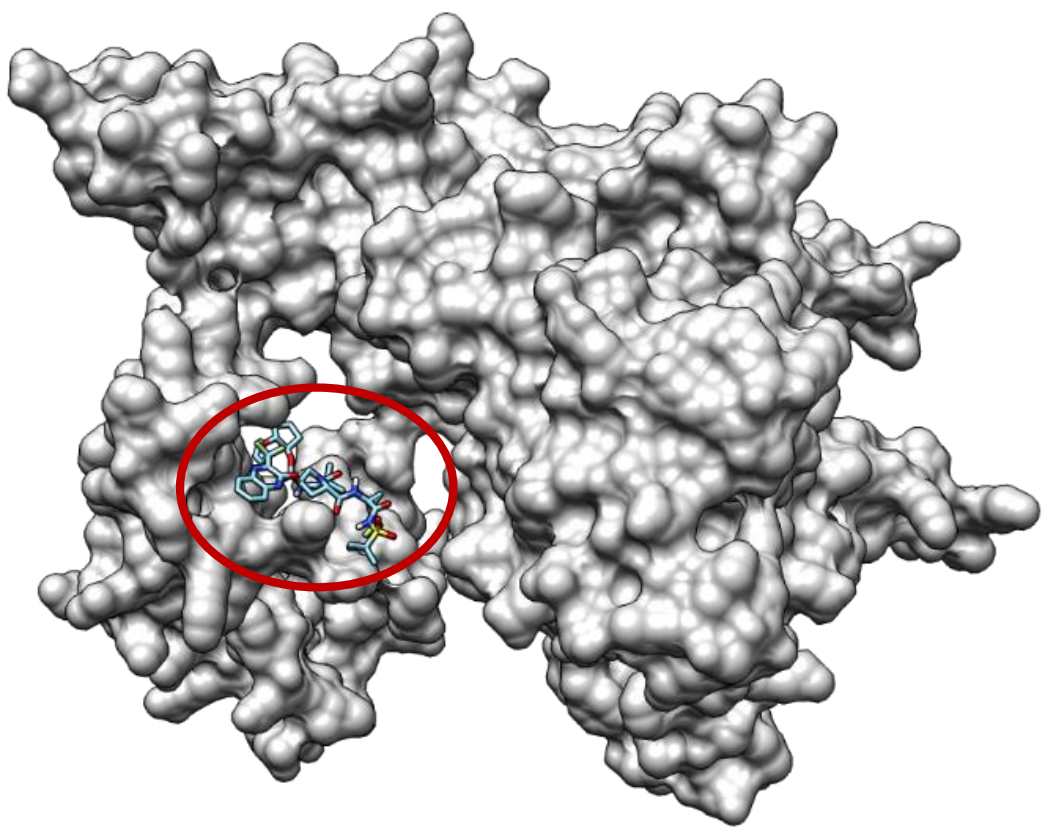

Gad1p – Glecaprevir

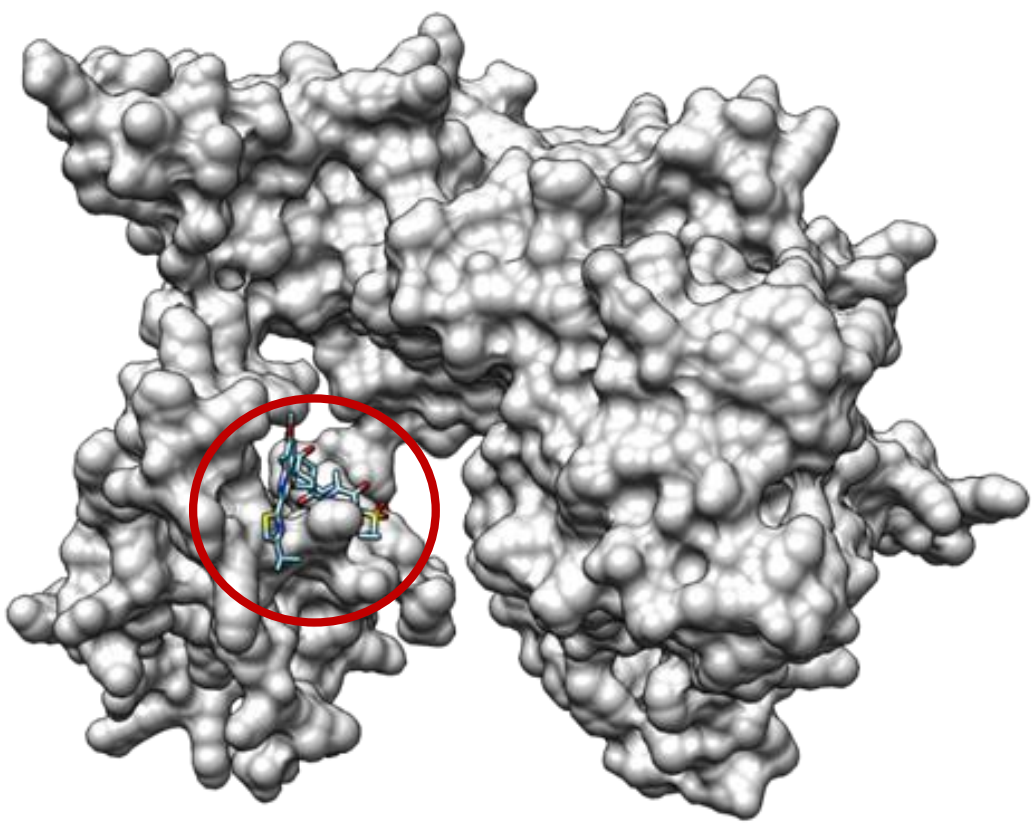

Gad1p – Simeprevir

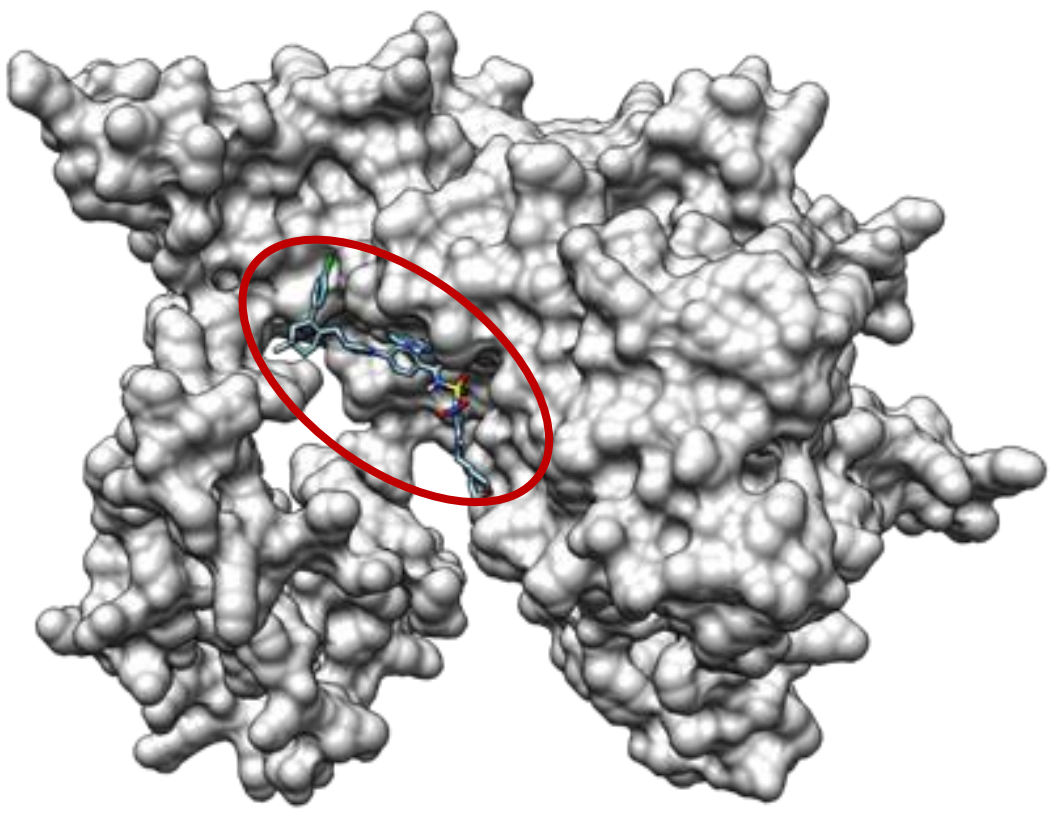

Gad1p – Venetoclax

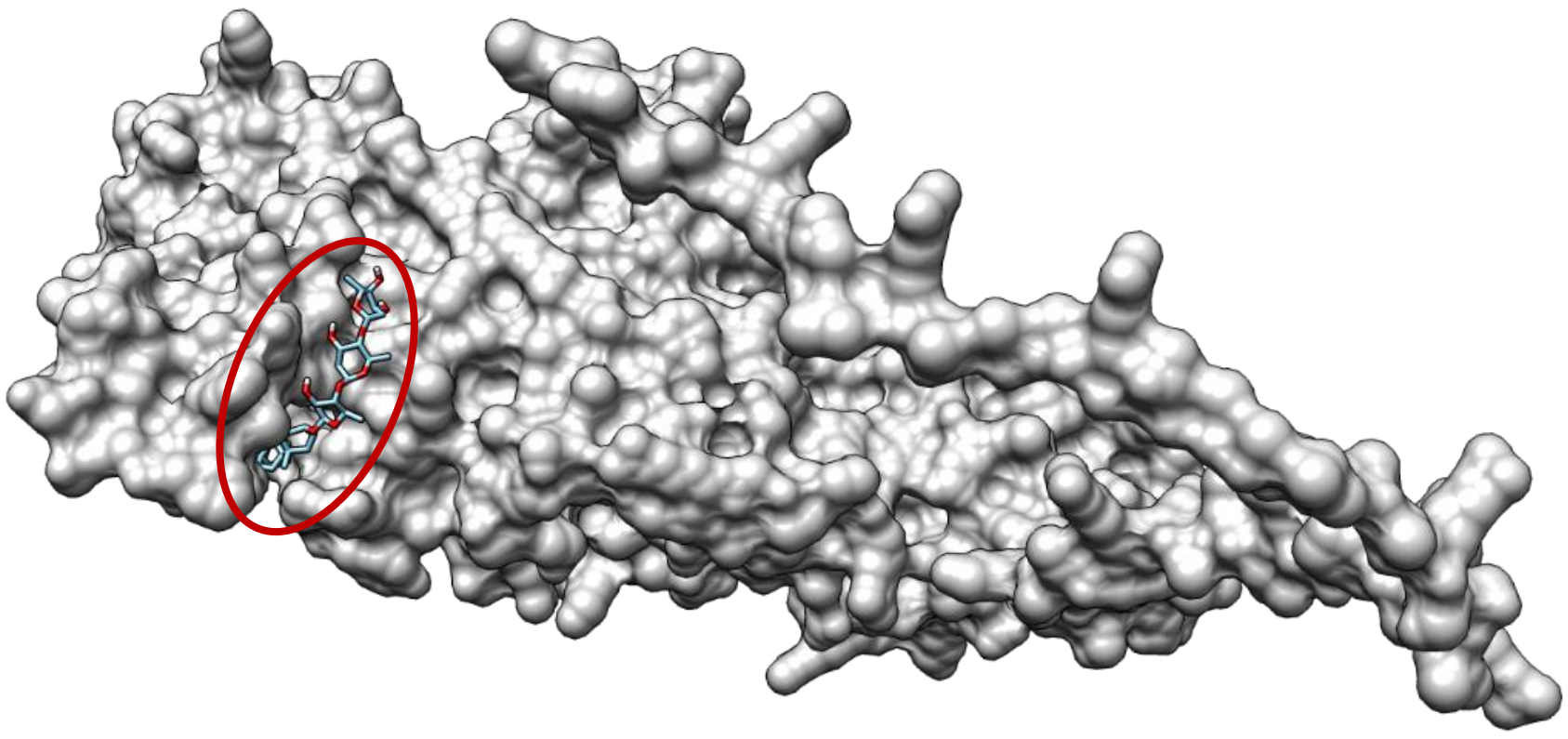

Atp1p – Digitoxin

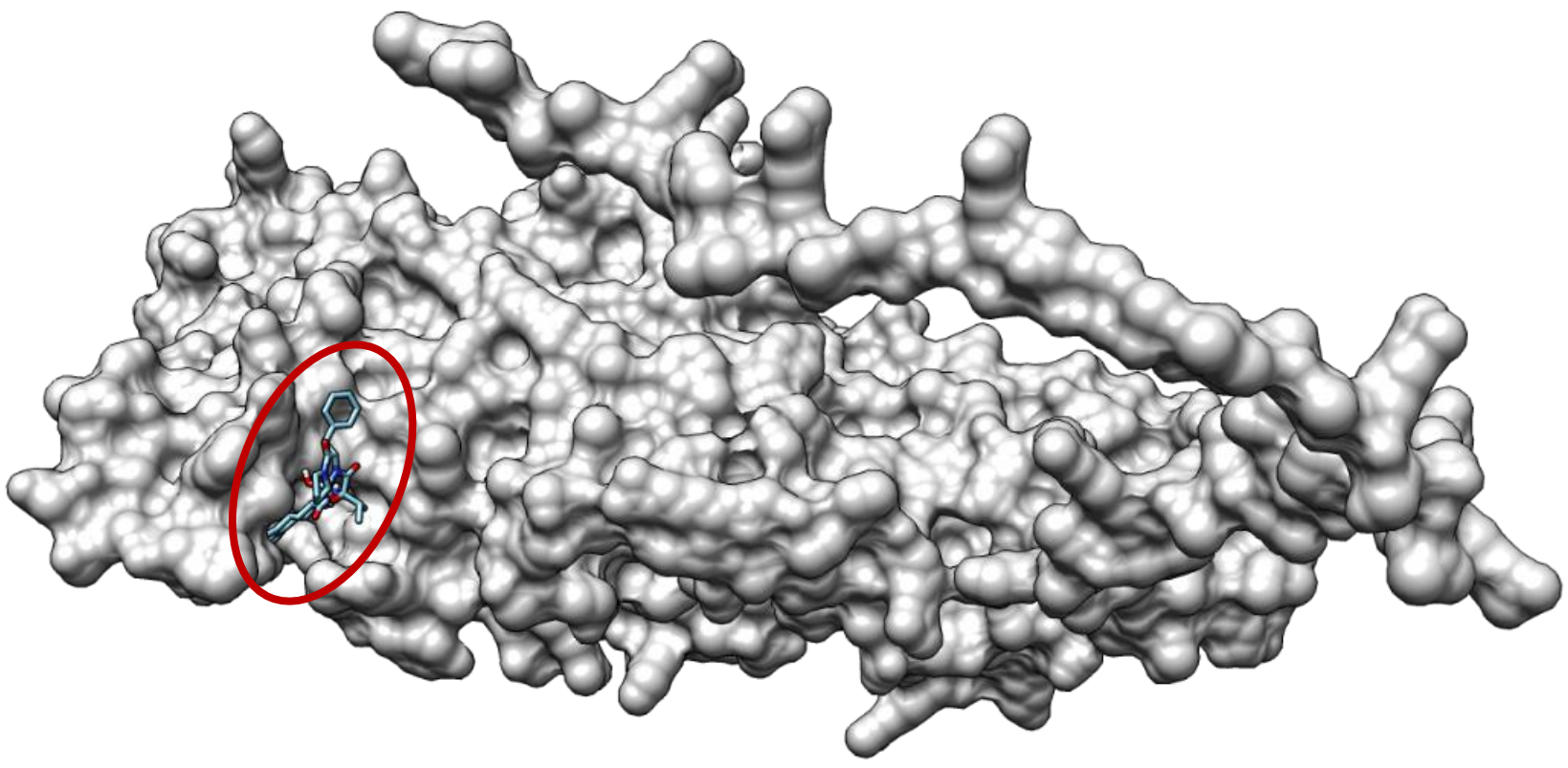

Atp1p – Dihydroergocristine mesylate

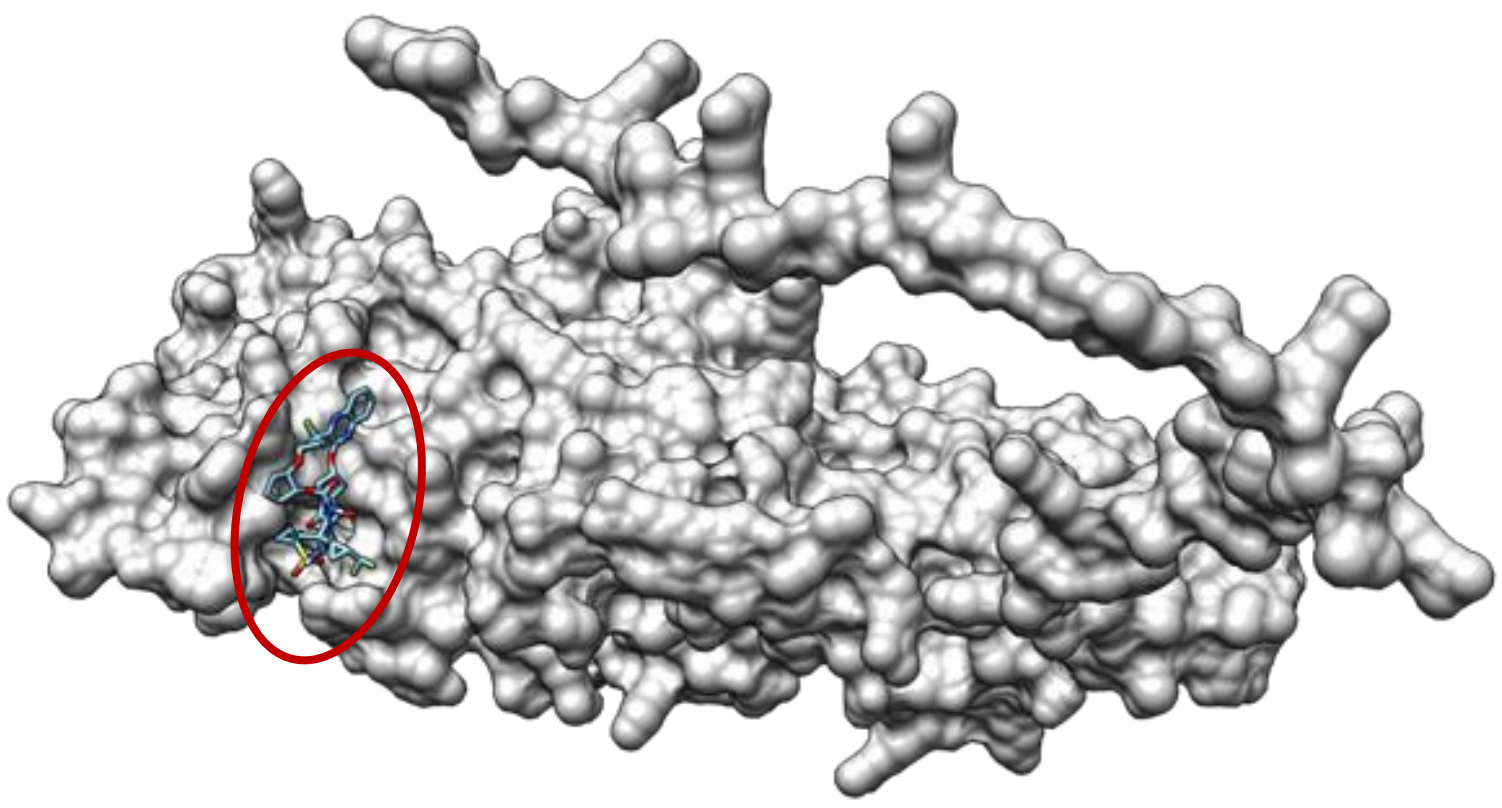

Atp1p – Glecaprevir

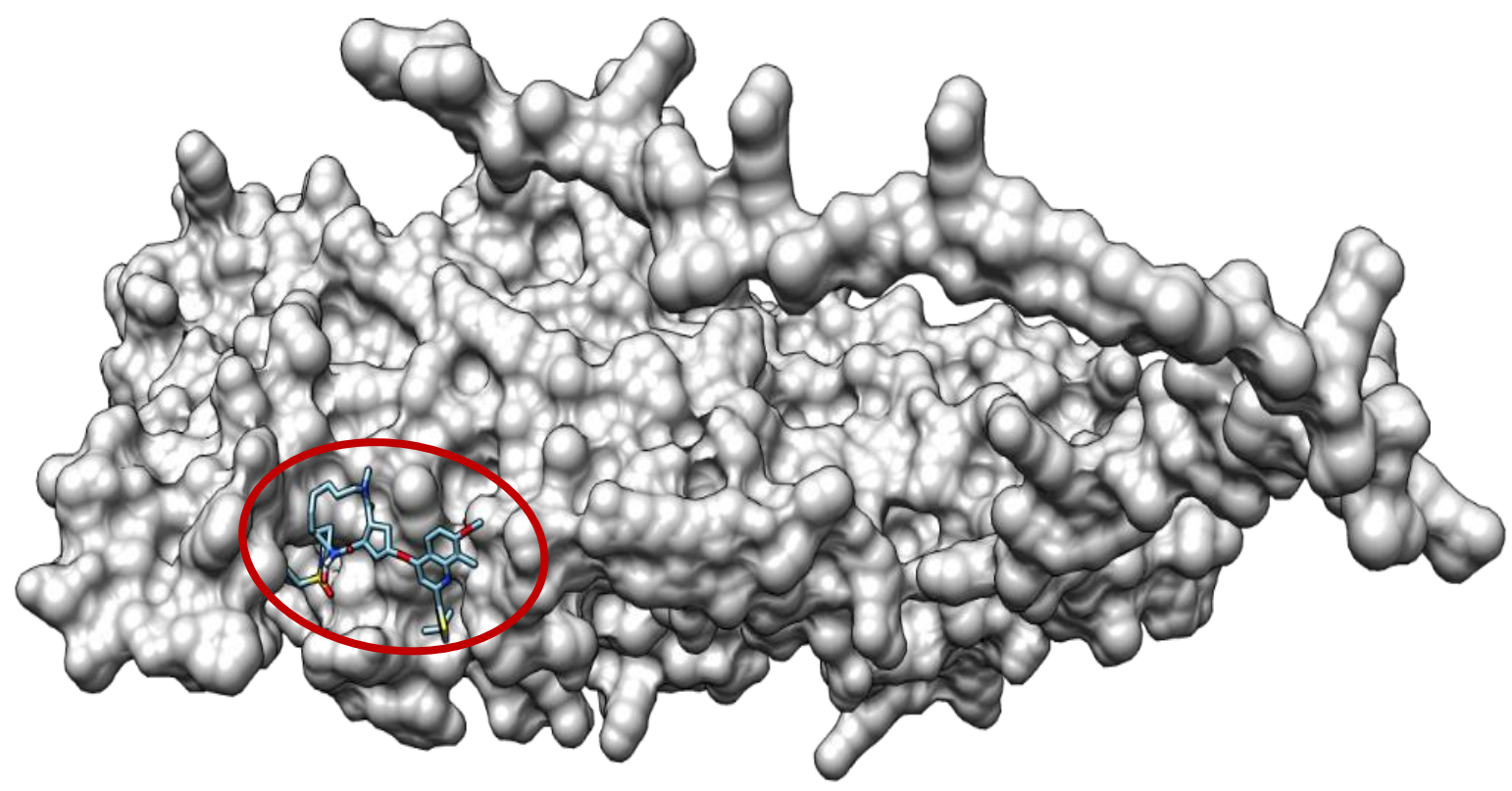

Atp1p – Simeprevir

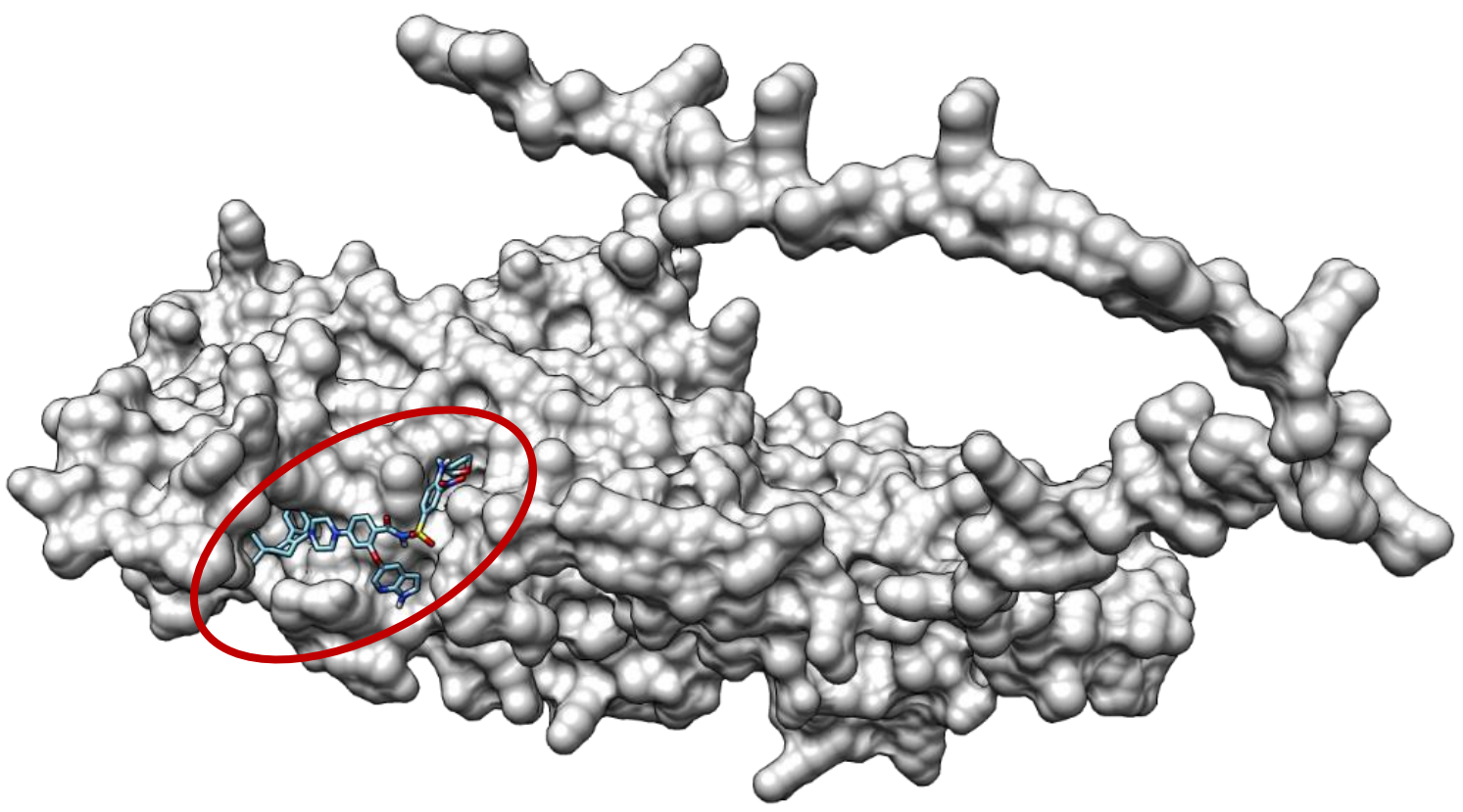

Atp1p – Venetoclax

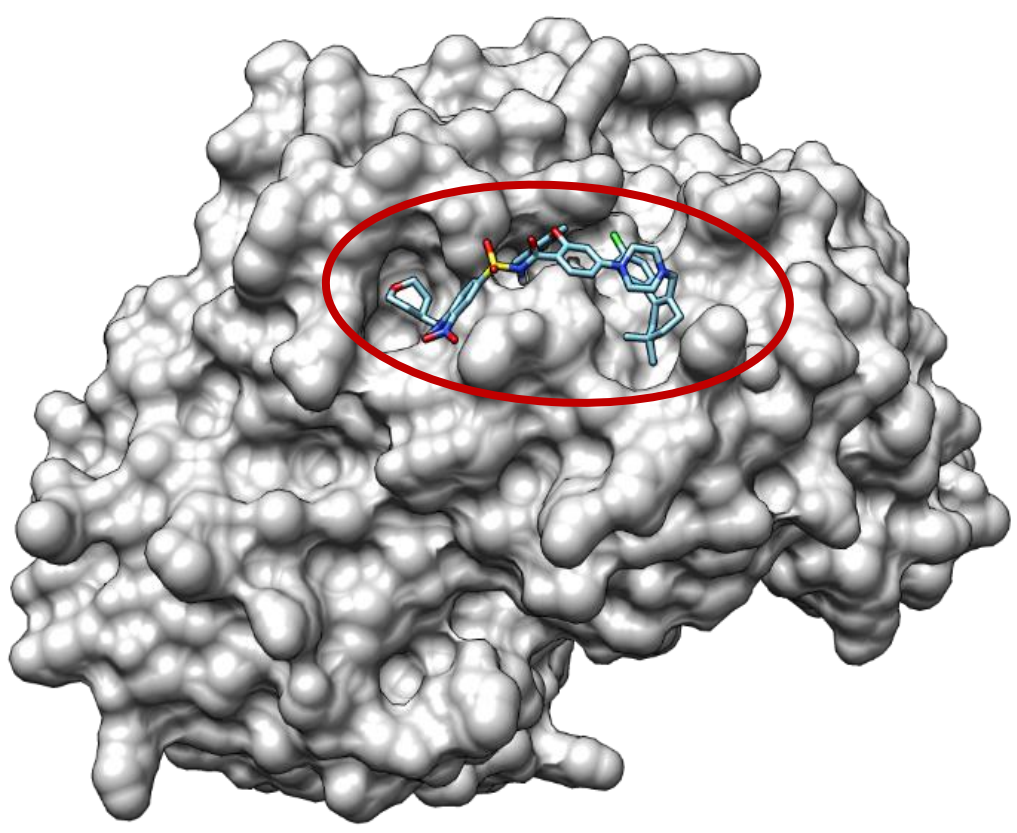

Eno1p – Venetoclax

**Fig S2** Overview of compounds exhibiting strong predicted interactions with NFAP2-associated binding pockets in *Candida albicans*. Compounds identified by structure-based virtual screening using the DrugRep server that met predefined binding energy thresholds for strong interaction with NFAP2-associated targets are shown. Strong interactions were defined as binding energies < −7 kcal/mol for glutamate decarboxylase (Gad1p), < −8 kcal/mol for ATP

synthase subunit alpha (Atp1p), and < −7.5 kcal/mol for enolase (Eno1p). Based on these criteria, digitoxin, dihydroergocristine mesylate, glecaprevir, simeprevir, and venetoclax and were identified as top candidates exhibiting strong predicted interactions with one or more NFAP2-associated binding pockets. Binding energies were calculated as part of the structure-based virtual screening workflow (Table 1). The binding site of the repurposed compound is highlighted with a red circle.
